# Prenatal BPA exposure perturbs RNA-binding protein-mediated splicing regulation and synaptogenesis in the developing cerebellum in a sex-dependent manner

**DOI:** 10.1186/s13293-026-00941-6

**Published:** 2026-06-22

**Authors:** Thanawin Jantheang, Songphon Kanlayaprasit, Kwanjira Songsritaya, Pawinee Panjabud, Pattanachat Lertpeerapan, Kasidit Kasitipradit, Surangrat Thongkorn, Depicha Jindatip, Valerie W. Hu, Thanit Saeliw, Tewarit Sarachana

**Affiliations:** 1 The Ph.D. Program in Clinical Biochemistry and Molecular Medicine, Department of Clinical Chemistry, Faculty of Allied Health Sciences, Chulalongkorn University, Bangkok, 10330 Thailand; 2 Chulalongkorn Autism Research and Innovation Center of Excellence (ChulaACE), Department of Clinical Chemistry, Faculty of Allied Health Sciences, Chulalongkorn University, Bangkok, 10330 Thailand; 3 Excellence Center for Genomics and Precision Medicine, King Chulalongkorn Memorial Hospital, The Thai Red Cross Society, Bangkok, 10330 Thailand; 4 The M.Sc. Program in Clinical Biochemistry and Molecular Medicine, Department of Clinical Chemistry, Faculty of Allied Health Sciences, Chulalongkorn University, Bangkok, 10330 Thailand; 5 Research Unit of Immunomodulation of Natural Products, Department of Transfusion Medicine and Clinical Microbiology, Faculty of Allied Health Sciences, Chulalongkorn University, Bangkok, 10330 Thailand; 6 Department of Biotechnology and Biomedicine (DTU Bioengineering), Technical University of Denmark, 2800 Kongens Lyngby, Denmark; 7 Department of Anatomy, Faculty of Medicine, Chulalongkorn University, Bangkok, 10330 Thailand; 8 Department of Biochemistry and Molecular Medicine, School of Medicine and Health Sciences, The George Washington University, Washington, DC 20037 USA

**Keywords:** Sex difference, Autism spectrum disorder, Endocrine-disrupting chemical, Bisphenol A, Cerebellum, Alternative splicing, RNA-binding proteins

## Abstract

**Background:**

Autism spectrum disorder (ASD) is a pervasive neurodevelopmental condition characterized by social communication deficits, exhibiting a male bias in prevalence. Emerging evidence suggests that prenatal exposure to bisphenol A (BPA) may perturb neurodevelopmental trajectories relevant to ASD. While the cerebellum is increasingly recognized as a brain region implicated in ASD pathophysiology, the impact of gestational BPA exposure on its post-transcriptional alternative splicing machinery remains fundamentally undefined.

**Methods:**

Here, we investigated sex-dependent effects of prenatal BPA exposure on the alternative splicing landscape of the neonatal rat cerebellum. We utilized RNA-seq to profile differential alternative splicing (DAS) events. Ingenuity Pathway Analysis (IPA) was used to predict biological functions and canonical pathways, and to construct the interactome network of DAS genes. To explore candidate upstream regulatory mechanisms, we performed in silico molecular docking and used high-resolution melting (HRM) qRT-PCR to validate selected splicing events. Furthermore, we assessed in vitro cellular phenotypes in primary cerebellar neurons by measuring MTS-based viability and Syn1/Psd95 puncta colocalization.

**Results:**

Prenatal BPA exposure was associated with widespread DAS in genes enriched for ASD-relevant pathways in the neonatal rat cerebellum. To our knowledge, this study is the first to report molecular docking analyses predicting favorable interactions between BPA and several candidate RNA-binding proteins (RBPs), including CPEB1, RALYL, HNRNPDL, and ACO1. Our findings support a model in which BPA may perturb RBP-associated splicing regulation, including altered splicing of chromatin regulators such as *Ccar1* in males. These molecular and cellular findings were accompanied by sex-stratified differences in neuronal viability and synaptic puncta measurements. BPA exposure was associated with an increased MTS viability signal in male primary cerebellar neurons, together with significant reductions in Psd95 and Syn1 puncta density, whereas female neurons showed significantly increased synaptic puncta colocalization together with reduced viability.

**Conclusions:**

In this study, we propose that prenatal BPA may be relevant to ASD-related neurodevelopmental pathways through sex-dependent changes in RBP-associated alternative splicing, including altered splicing of *Ccar1* in males, together with distinct cellular outcomes. Together, these findings identify the developing cerebellum as a sensitive target of prenatal BPA exposure and highlight alternative splicing as a candidate pathway relevant to ASD biology.

**Supplementary Information:**

The online version contains supplementary material available at https://doi.org/10.1186/s13293-026-00941-6.

## Abstract (plain language summary)

Autism spectrum disorder (ASD) is a neurodevelopmental condition influenced by genetic and environmental factors, including bisphenol A (BPA), a ubiquitous chemical found in daily life. Previous studies have linked prenatal BPA exposure to an increased risk of ASD, especially in the hippocampus and prefrontal cortex. However, the exact molecular mechanisms of how BPA contributes to ASD pathogenesis in the cerebellum remain unclear. In this study, we investigated whether prenatal BPA exposure disrupts the regulatory mechanisms of alternative splicing in the developing rat cerebellum. This study provides evidence that prenatal BPA exposure is associated with altered alternative splicing of chromatin regulator genes, including *Ccar1*, in the cerebellum of offspring. Moreover, these differentially spliced genes were associated with crucial neurological functions and disorders, including synaptic development and motor dysfunction related to ASD. Furthermore, this study provides evidence that prenatal BPA exposure is associated with sex-dependent alterations in alternative splicing in the cerebellum, and computational analyses suggest that BPA may interact with candidate RBPs involved in splicing regulation. In male offspring, altered splicing of the exon encoding the SAP domain of *Ccar1* was identified in the cerebellum, accompanied by sex-dependent differences in neuronal viability-related and synaptic puncta measurements in primary cerebellar neurons, whereas female neurons showed increased synaptic puncta colocalization together with reduced viability. These findings suggest that prenatal BPA exposure may affect critical gene splicing through RBP-associated mechanisms and may contribute to sex-dependent neurodevelopmental processes in the cerebellum.

Prenatal BPA exposure was associated with altered alternative splicing in the cerebellum, including mis-splicing of the exon encoding the SAP domain of *Ccar1* in males.

Prenatal BPA exposure was associated with widespread differential alternative splicing in the developing cerebellum, including genes enriched in ASD-relevant pathways.

Computational analyses identified candidate RNA-binding proteins that may participate in BPA-associated splicing dysregulation, including altered exon skipping of *Ccar1* in the male cerebellum.

**Supplementary Information:**

The online version contains supplementary material available at https://doi.org/10.1186/s13293-026-00941-6.

## Highlights

Prenatal BPA exposure was associated with altered alternative splicing in the cerebellum, including mis-splicing of the exon encoding the SAP domain of *Ccar1* in males.

Prenatal BPA exposure was associated with widespread differential alternative splicing in the developing cerebellum, including genes enriched in ASD-relevant pathways.

Computational analyses identified candidate RNA-binding proteins that may participate in BPA-associated splicing dysregulation, including altered exon skipping of *Ccar1* in the male cerebellum.

**Supplementary Information:**

The online version contains supplementary material available at https://doi.org/10.1186/s13293-026-00941-6.

## Introduction

Autism Spectrum Disorder (ASD) is a complex neurodevelopmental disorder characterized by two main core symptoms: deficits in social communication and interaction, along with restricted or repetitive behaviors, interests, or activities [1]. These features often appear before age three and include difficulties with eye contact, speech abnormalities, and stereotypic movements. With a steadily increasing prevalence, currently estimated at 1 in 31 children under the age of eight in the United States [2], ASD poses a significant public health challenge. Notably, ASD has a sex ratio of approximately 3–4:1, with males being diagnosed more frequently than females [3, 4]. While ASD is primarily defined by social and behavioral deficits, affected individuals often display a wider range of impairments, including significant deficits in executive functioning [5] and highly comorbid motor dysfunctions [6, 7]. Historically viewed solely as a motor control center, the cerebellum is now increasingly recognized for its essential role in coordinating both motor functions and higher-order cognitive processes [8, 9]. Emerging clinical evidence suggests that early cerebellar and motor deficits are associated with later difficulties in social interaction and communication [10, 11]. Consistent with this motor-to-social connection, post-mortem studies have identified cerebellar abnormalities, particularly the significant loss of Purkinje cells, as one of the most robust anatomical hallmarks of the brain of individuals with ASD [12]. Although the etiology of ASD remains incompletely understood, genetic, epigenetic, and environmental factors have all been reported as potential risk factors for ASD. Genetic studies highlight the roles of de novo mutations [13], chromosomal abnormalities [14], and copy number variations [15, 16], while epigenetic alterations—including abnormal DNA methylation [17, 18], histone modifications [19], non-coding RNAs (such as microRNAs and LncRNAs) [20–23] and disrupted RNA splicing—further modulate the risk of ASD [20, 24–27]. Our previous integrated methylation and transcriptome profiling analyses suggested that LINE-1 and Alu methylation are involved in epigenetic regulatory networks associated with ASD [28, 29]. This susceptibility is further modulated by environmental risk factors, particularly maternal exposure to endocrine-disrupting chemicals (EDCs) during critical windows of fetal brain development [3, 30–32]. 

Bisphenol A (BPA), a pervasive synthetic EDC, is widely utilized in polycarbonate plastics and epoxy resin products [33]. BPA can enter the body through ingestion [34, 35], inhalation [36, 37], or dermal absorption [25, 38–40] and is capable of crossing both the placenta and blood–brain barrier [41, 42]. Historically, the U.S. FDA cited 5,000 µg/kg/day as a no-observed-adverse-effect level (NOAEL) for BPA [43]; however, emerging evidence suggests that maternal exposures at or below this dose may still be associated with transcriptomic and behavioral alterations in offspring [44, 45]. Previous studies from our laboratory have shown that prenatal BPA exposure disrupts gene expression and is associated with behavioral abnormalities in rodent models. Notably, prenatal BPA exposure leads to dysregulation of key ASD candidate genes such as *Auts2*, *Foxp2*, *Sema5a*, and *Slc9a9*, with distinct patterns observed between male and female offspring [46–48]. The expression of transcription factors (TFs) such as AR, ESR1, and RORA is dysregulated, influencing the transcriptional landscape of ASD-related genes [48, 49]. Furthermore, prenatal BPA exposure significantly alters transcriptomic profiles in hippocampal neural stem cells and disrupts genes associated with cortical development, changes that may contribute to sex-dependent ASD-like phenotypes [50, 51]. In addition, behavioral studies reveal that these transcriptomic changes correlate with deficits in social behaviors and learning, particularly in males, highlighting the functional consequences of BPA-induced gene dysregulation [48]. Furthermore, early toxicological evidence suggests that BPA exposure can reduce Purkinje cell populations and alter synapse formation in cerebellar neurons, positioning the cerebellum as a highly vulnerable yet underexplored target of EDC neurotoxicity [52–54].

Beyond overall gene expression levels, alternative splicing (AS) is a critical post-transcriptional process that generates the essential protein diversity required for highly complex cellular functions in the developing brain [55, 56]. Splicing events are exquisitely orchestrated by RNA-binding proteins (RBPs), which dictate the inclusion or skipping of specific exons [57, 58]. Notably, widespread dysregulation of this alternative splicing machinery is recognized as a molecular hallmark of ASD [26, 59, 60]. In addition, our previous studies demonstrated that prenatal BPA exposure dysregulates the alternative splicing pattern of ASD-related genes in the hippocampus and prefrontal cortex of neonatal rats, leading to aberrant social behaviors related to ASD [61, 62]. Notably, BPA is well documented to act as a xenoestrogen that interacts with classical nuclear receptors (e.g., ESR1, YY1) [48, 49, 61, 62] to indirectly alter gene expression networks. To our knowledge, no prior studies have investigated the direct binding interactions between BPA and RBPs. We hypothesize that BPA may act not only through classical receptor-mediated pathways but also by perturbing RBP-associated alternative splicing networks, leading to dysregulated alternative splicing and functional deficits in the cerebellum. 

**Fig. 1:**
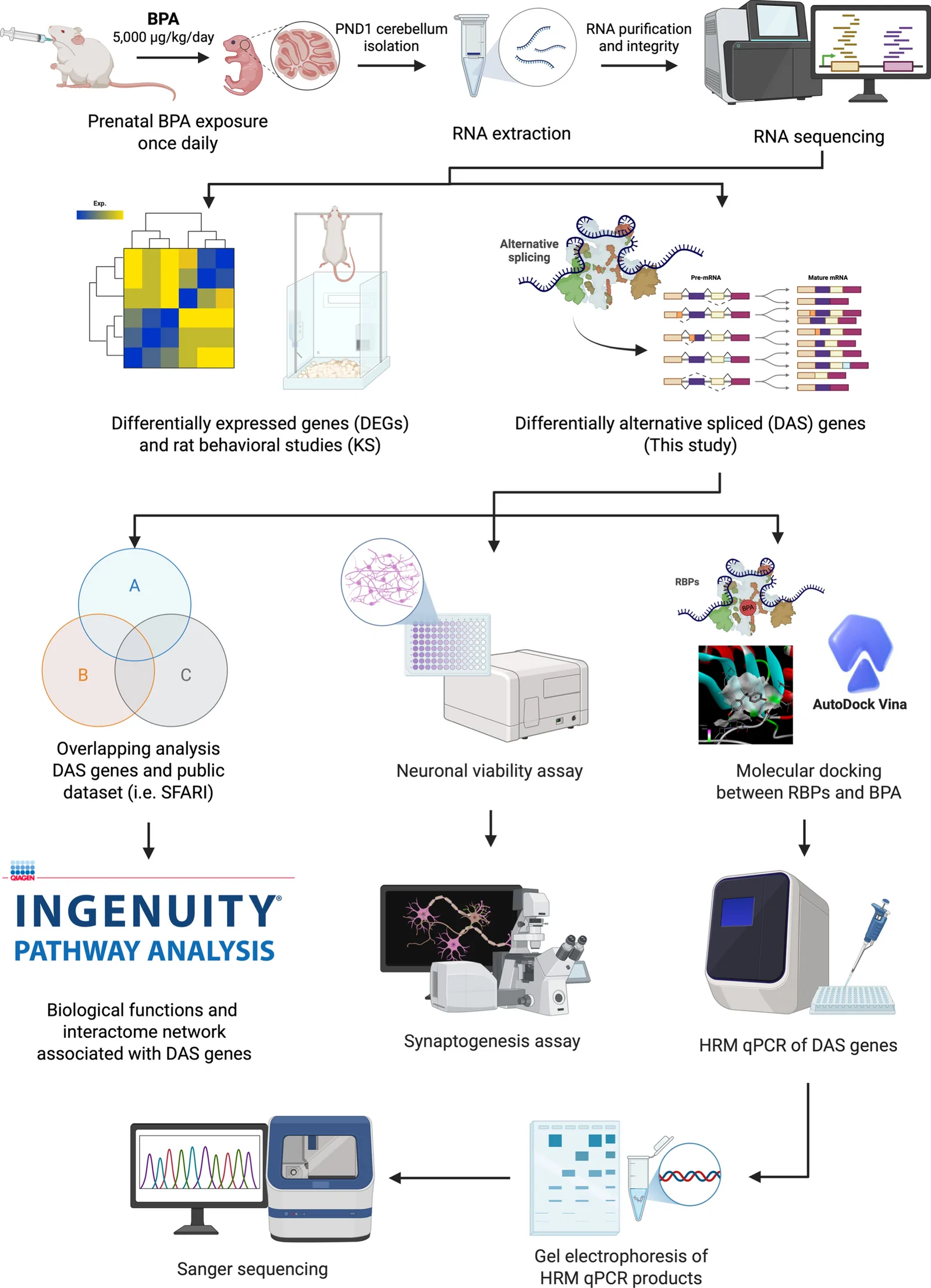
Overview of the study design. Pregnant Wistar Furth rats received BPA (5,000 µg/kg/day) or vehicle control by oral gavage daily from gestational day 1 until parturition. Cerebella were isolated from neonatal rat pups at postnatal day 1 (PND1), and RNA was extracted and assessed for quality and integrity prior to RNA sequencing. Differentially alternative spliced (DAS) genes identified from RNA-seq were subjected to three parallel downstream analyses: (i) pathway enrichment analysis using Ingenuity Pathway Analysis (IPA) to predict biological functions and interactome networks associated with DAS genes; (ii) primary cerebellar neuron assays including neuronal viability and synaptogenesis assessments; and (iii) in silico RBP motif enrichment analysis and molecular docking simulations using AutoDock Vina to identify candidate RNA-binding proteins (RBPs) and predict BPA–RBP interactions. Candidate DAS genes identified from RNA-seq were validated at the transcript level by high-resolution melting (HRM) qRT-PCR, with amplicon identity confirmed by gel electrophoresis and Sanger sequencing. Figure created in https://BioRender.com

To address this critical gap, this study investigates the impact of prenatal BPA exposure on alternative splicing patterns of the developing neonatal rat cerebellum. Utilizing a Wistar Furth rat model, we conducted a comprehensive RNA-sequencing analysis to identify differentially alternatively spliced (DAS) genes. We further integrated these findings with Ingenuity Pathway Analysis (IPA) [60] to map BPA-responsive DAS genes onto established ASD-related neurodevelopmental and motor pathways. Importantly, we combined in silico molecular docking analysis of BPA with candidate regulatory RBPs and high-resolution melting (HRM) qRT-PCR to examine predicted BPA–RBP interactions and validate dysregulated alternative splicing events in candidate DAS genes. Finally, we examined whether these molecular findings were accompanied by cellular phenotypes using primary cerebellar neuron cultures, explicitly assessing neuronal viability and synaptogenesis to clarify the distinct, sex-specific trajectories of prenatal BPA exposure in the cerebellum, as shown in Fig. 1.

## Methods

### Animal husbandry

All animal experimental procedures were reviewed and approved by the Institutional Animal Care and Use Committee (IACUC) of Chulalongkorn University (Animal Use Protocol no. 1673007, 1773011, 2073011, and 2273007). Eight-week-old male and female Wistar Furth rats were obtained from the National Laboratory Animal Center (NLAC), Thailand, and were quarantined in sex-separated cages for 1 week prior to any procedures. All Wistar Furth rats were housed at the Chulalongkorn University Laboratory Animal Center (CULAC) under standard conditions (21 ± 1℃) and humidity (30–70%) in a 12-h light/dark cycle. Standard rodent chow and tap water were provided ad libitum. Female rats were mated with males and then assigned to either the BPA treatment group or the control group. For the BPA group, female rats received BPA via oral gavage at a concentration of 5,000 μg/kg body weight daily, from gestational day 1 (GD1) until parturition. Control dams received the same vehicle and vehicle volume used for BPA administration. 

### Cerebellum isolation, RNA extraction, and purification

The cerebellum was isolated from neonatal rat pups at postnatal day 1 (PND1) in both the BPA-treated and control groups. Rat pups were deeply anesthetized using Isoflurane and euthanized by hypothermia. Decapitation was performed to confirm the death, and the whole brain was harvested into a 60 mm dish containing 15 mL of pre-chilled dissection solution A (1X HBSS containing 30 mM glucose, 2 mM HEPES, and 26 mM NaHCO_3_). Meninges were completely removed, and the cerebellum was aseptically and gently separated from the midbrain and brainstem. The cerebellum was transferred into RNAlater (Thermo Fisher Scientific, Waltham, MA, USA) to stabilize RNA and stored at -80 °C. RNA extraction was performed using the mirVana™ miRNA Isolation Kit (Invitrogen, Thermo Fisher Scientific, Waltham, MA, USA). RNA quantity and purity were assessed using a NanoDrop spectrophotometer (Thermo Fisher Scientific, Waltham, MA, USA).

### RNA sequencing and differential alternative splicing analyses

RNA extracted from the postnatal day 1 (PND1) rat pup cerebellum of both BPA-treated and vehicle control groups (n = 3 biological replicates per sex per treatment group, each representing one individual pup derived from an independent litter; 1 technical sequencing replicate per sample) was subjected to quality assessment prior to sequencing. Only samples meeting the following criteria were included: total RNA ≥ 200 ng, A260/280 ≥ 2.0 and A260/230 ≥ 2.0, RNA concentration ≥ 20 ng/µl, RNA integrity number (RIN) ≥ 7.0, and 28S/18S ≥ 1.0. Qualifying RNA samples were submitted to BGI Genomics (Shenzhen, China) for polyA-selected library preparation and paired-end 100 bp sequencing (PE100) on the DNBSEQ platform. Raw reads were filtered using SOAPnuke (BGI internal pipeline) to remove reads containing adapter sequences, reads with more than 10% unknown bases (N), and low-quality reads (base quality score < 5 in more than 50% of bases). Clean reads were aligned to the Rattus norvegicus reference genome (NCBI, GCF_000001895.5, Rnor_6.0) using HISAT2 with default parameters. Alternative splicing events were detected and quantified using rMATS (replicate Multivariate Analysis of Transcript Splicing) with default parameters, applied separately to the female-only (Ctrl-F vs. BPA-F), male-only (Ctrl-M vs. BPA-M), and combined-sexes (Control vs. BPA-treatment) comparisons. Five categories of splicing events were assessed: skipped exons (SE), alternative 5’ splice sites (A5SS), alternative 3’ splice sites (A3SS), mutually exclusive exons (MXE), and retained introns (RI). Differential alternative splicing events were defined by FDR < 0.05. The ΔPSI (change in Percent Spliced In) was calculated as PSI(BPA) − PSI(Control), where PSI = inclusion counts / (inclusion counts + skipping counts).

### Prediction of biological functions, canonical pathways, and interactome networks associated with DAS genes

We performed pathway enrichment analysis by uploading DAS gene lists to Ingenuity Pathway Analysis (IPA) software (QIAGEN Inc., Redwood City, CA, USA; https://digitalinsights.qiagen.com/IPA). The gene lists were analyzed against the Ingenuity Knowledge Base. Diseases and functions, disorders, canonical pathways, and interactome networks were predicted from the uploaded lists of the DAS genes with associated molecules, and the relationships within the network were identified, divided according to the sex of the RNA-seq sample, including male, female, and combined-sexes analyses. Statistical significance for pathway enrichment was determined using Fisher’s exact test, with a P-value < 0.05 established as the threshold for significance. To complement the primary IPA analysis with threshold-free and threshold-relaxed approaches, two additional enrichment analyses were performed. First, supplementary over-representation analysis was conducted using EnrichR against the GO Biological Process 2026, GO Cellular Component 2026, GO Molecular Function 2026, SynGO 2024, and Disease Perturbations from GEO databases, applied to male and female skipped exon (SE)-type DAS gene sets at a relaxed threshold of FDR < 0.10. Second, threshold-free gene set enrichment analysis was performed using the fgsea R package (v1.26.0) with detectable exon-skipping splicing events ranked by signed ΔPSI (positive to negative, reflecting exon inclusion to skipping) for directional enrichment and by |ΔPSI| for undirected enrichment, separately for male and female analyses. Gene sets were derived from MSigDB collections including GO Biological Process, GO Cellular Component, GO Molecular Function, and Human Phenotype Ontology terms (minimum 15, maximum 500 genes per set), and significance was defined as padj < 0.05 after Benjamini–Hochberg correction. Complete results from both analyses are provided in Additional Files 9 and 10.

### In-silico RBP motif enrichment and regulatory mapping

To identify upstream regulators of the aberrant alternative splicing of the candidate genes, local genomic sequences were acquired utilizing the UCSC Genome Browser API [63]. For each target alternative splicing event, the precise exon coordinates were mapped to the Rattus norvegicus reference genome (Rn6). To comprehensively capture splice-site regulatory elements, the target exon sequences were obtained along with a 300-base pair flanking region spanning both upstream and downstream intronic sequences. A high-confidence, comprehensive RNA-binding protein (RBP) motif database was constructed using a dual-source approach. First, empirically validated Position Weight Matrices (PWMs) were downloaded from the CISBP-RNA (Catalog of Inferred Sequence Preferences of RNA-Binding Proteins) database [64], then converted to consensus RNA binding motifs. To ensure biological relevance, the database was rigorously filtered to retain only functionally annotated RBPs from *Rattus norvegicus*, *Mus musculus,* and *Homo sapiens*, excluding uncharacterized or redundant loci. This library was subsequently supplemented with a manually curated matrix of canonical neurodevelopmental and ASD-associated RBPs (e.g., ELAVL, MBNL, and NOVA families), with binding motifs sourced from the ATtRACT database, a curated repository of experimentally validated RNA-binding protein motifs derived from CLIP-seq and in vitro binding studies by Giudice et al. [65], to ensure comprehensive regulatory coverage. A Python-based regular expression (regex) scanning algorithm was deployed to interrogate these localized pre-mRNA sequences against the comprehensive RBP motif library. The algorithm quantified the absolute frequency of exact sequence matches for each consensus motif within the defined regulatory windows to establish a binding site density metric for each candidate RBP.

### Molecular docking analysis and competitive binding assay

To estimate the relative docking preference of BPA versus a simplified RNA proxy at candidate RBP interfaces, the experimentally validated consensus RNA-binding motif for each candidate RBP was used as the competing ligand obtained from the ATtRACT database. For each RBP, the first ribonucleoside of its consensus sequence was prepared as a representative competing ligand using OpenBabel (v3.1.1) with MMFF94 force field 3D coordinate generation. This approach provides a simplified estimate of competitive binding potential at the RNA-recognition interface, consistent with the broader framework of small molecule–RBP interaction studies [66]. BPA and the consensus ribonucleoside were independently docked against each AlphaFold protein structure using blind docking (full-protein grid box, exhaustiveness = 16, n = 3 triplicate runs).

### Target protein structure acquisition and preparation

The three-dimensional structures of the candidate RBPs identified from the RNA-seq analysis were acquired from the AlphaFold Protein Structure Database [67, 68]. Target selection was prioritized using UniProt exact gene matching for the experimental species (Rattus norvegicus), defaulting to orthologous mammalian structures (*Mus musculus* or *Homo sapiens*) when rat-specific high-confidence structures were unavailable. The AlphaFold structure identifiers for all RBPs used in the docking simulations are provided in Additional File 7. The raw PDB files were converted to PDBQT format using OpenBabel (v.3.1.0) [69], during which Gasteiger partial charges were calculated and assigned to the receptor atoms.

### Molecular docking

Molecular docking simulations were executed using the Python bindings of AutoDock Vina (v.1.2.0) [70, 71]. To ensure an unbiased assessment of ligand–protein interactions without prior assumptions of the binding pockets, a global blind docking approach was employed. For each target RBP, the center of the grid box was dynamically calculated as the mean geometric coordinate of all protein atoms. The grid box dimensions were defined by the maximum distance between protein atoms across all axes, augmented with a 20.0 Å to ensure the entire protein surface and internal cavities were encompassed within the search space. To ensure reproducibility, each ligand-receptor pair underwent triplicate independent docking simulations (n = 3). The lowest binding energy (expressed as ΔG in kcal/mol) from the generated poses was recorded for each replicate, and the average binding affinity was calculated. To estimate relative docking preference, the predicted binding affinity of BPA was compared with that of the first ribonucleoside from each RBP consensus motif used as a simplified RNA proxy.

### Primary cerebellar neuron culture

Primary cerebellar neuron culture was adapted and conducted according to the culture protocol described by Li J. et al., 2010 [72], and Guo et al., 2012 [73]. Briefly, neonatal rat pups at postnatal day 1 (PND1) were deeply anesthetized using isoflurane, then euthanized by hypothermia, and sacrificed. The whole brain was removed and transferred into a 60 mm dish containing 15 mL of pre-chilled dissection solution (1X HBSS; Invitrogen, Thermo Fisher Scientific, Waltham, MA, USA). The whole brain was dissected under a Nikon SMZ18 Stereo Microscope (Nikon, Tokyo, Japan). The meninges were removed, and the cerebellum was aseptically and gently separated from the midbrain and brainstem. The cerebellum was cut into 5 small pieces using a sterile blade and transferred into a 15 ml pooling tube containing 10% FBS-containing Neurobasal (NB) media (Gibco, Thermo Fisher Scientific, Waltham, MA, USA). The cerebellar tissue was then digested by digestion solution containing 1X HBSS, 0.25% trypsin (Thermo Fisher Scientific), and 0.05% DNase I (Sigma-Aldrich, St. Louis, MO, USA) for 3–5 min at room temperature. After tissue digestion, serum-containing NB medium (Neurobasal medium, 0.5% glucose, 2 mM glutamine, 1% penicillin–streptomycin, and 10% FBS serum) was added to stop the enzymatic digestion and was centrifuged at 1,500 rpm for 10 min at 4 °C. The tissue was resuspended in a trituration solution containing 0.05% DNase I and FBS serum-containing NB medium. Tissue was gently triturated into dissociated cells by using a sterile fine glass pipette pre-burnt with flame. After trituration, the dissociated cells were strained through a 40-μm nylon mesh filter (Falcon, Corning Inc., Corning, NY, USA) in a 15 ml centrifuge tube containing 10% FBS in NB media to maintain the dissociated cells, then centrifuged, and resuspended in serum-free NB medium supplemented with insulin, transferrin, and sodium selenite. (Neurobasal medium, 0.5% glucose, 2 mM glutamine, 1% penicillin–streptomycin, 20 μg/ml insulin, 20 μg/ml transferrin, 20 ng/ml sodium selenite, and 2% B27 supplement; Gibco, Thermo Fisher Scientific). After cell resuspension, the cells were counted and seeded at the desired density according to each experiment. The primary cerebellar neuron culture was maintained at 37 °C with 5% CO_2_ in serum-free NB medium supplemented with insulin, transferrin, and sodium selenite for 14 days (DIV14), and the culture medium was replaced every 2 days by replacing half of the medium with freshly prepared medium.

### Primary cerebellar neuron viability assay

To assess the direct effects of BPA on cerebellar neuron viability, primary cerebellar neurons were isolated from neonatal rat pups at postnatal day 1 (PND1), prenatally treated with vehicle control only. Cells were maintained in the supplemented Neurobasal medium as described in the Primary Cerebellar Neuron Culture section, and the primary cerebellar neurons were isolated from 3 independent litters per sex per condition, with each litter constituting one biological replicate. Cells from each litter were seeded at 40,000 cells per well in a poly-L-lysine-coated 96-well plate, with 3 technical replicate wells per litter per BPA concentration. The well plate was incubated at 37 °C with 5% CO2 for 48 h. Half of the medium was then discarded and replaced with BPA-containing Neurobasal medium to achieve final concentrations of 0.01, 0.1, 1.0, or 10 ng/ml, or a vehicle control, and culture was continued for 24, 48, and 72 h. After each incubation period, 30 μl of CellTiter 96® Aqueous Reagent (Promega, Madison, WI, USA) was added to each well to measure cell viability. Absorbance at 490 nm was recorded at 0, 1, 2, 3, and 4 h post-reagent addition using a microplate reader (PerkinElmer, Waltham, MA, USA).

### Synaptogenesis assay

To assess the functional consequences of prenatal BPA exposure on synaptogenesis, primary cerebellar neurons were isolated at postnatal day 1 (PND1) from neonatal rat pups that had undergone prenatal in utero BPA exposure (5,000 µg/kg/day by maternal oral gavage from GD1 to parturition) or from vehicle control pups (male n = 3 litters per treatment group; female n = 3 litters per treatment group), yielding four experimental groups: male BPA, male control, female BPA, and female control. No additional BPA or vehicle was supplemented to the culture medium. The dissociated primary cells were counted and seeded at a density of 75,000 cells onto sterile, acid-washed 22 × 22 mm poly-L-lysine-coated coverslips and maintained as described in the Primary Cerebellar Neuron Culture section. The primary cells were maintained in Neurobasal medium as described in the method for primary cerebellar neuron culture for 14 days and fed every 2 days by removing half of the medium and replacing it with fresh medium. After DIV14 of culture, the media was discarded, and the coverslip was washed with cold sterile PBS. The cells were fixed with 4% PFA (Sigma-Aldrich, St. Louis, MO, USA) and incubated at 4 °C for 30 min, then washed three times with sterile PBS for 5 min each. After that, 0.1% Triton-X 100 in PBST solution was added to permeabilize the fixed cells at room temperature for 10 min. The coverslip was washed with filter PBS three times for 5 min each. 5% normal goat serum in PBST was used as a blocking solution for 30 min at room temperature. The blocking solution was removed, and the fixed cells were incubated with primary antibodies diluted in blocking solution, including 1:500 mouse anti-synapsin 1 antibody (ab8, Abcam, UK), 1:500 rabbit anti-Psd95 antibody (ab2723, Abcam, UK), and 1:1000 chicken anti-Map2 antibody (ab5392, Abcam, UK) at 4 °C overnight. On the next day, the fixed cells were washed with sterile PBS and incubated with 1:1000 Alexa Fluor-conjugated secondary antibodies including Donkey Anti-Rabbit IgG H&L (Alexa Fluor® 647, ab150063, Abcam, UK) preabsorbed, Donkey Anti-Mouse IgG H&L (Alexa Fluor® 488, ab150109, Abcam, UK) preabsorbed, Goat Anti-Chicken IgG H&L (Alexa Fluor® 555, ab150158, Abcam, UK) and 1:2000 Bisbenzimide H 33342 (Hoechst 33342, Invitrogen, Thermo Fisher Scientific) for 1 h, room temperature in the dark. The cell was washed three times with sterile PBS. The coverslip was mounted to the glass slide using ProLong™ Diamond Antifade Mountant (Invitrogen, Thermo Fisher Scientific), and the fluorescence signal for each protein marker was examined using an LSM800 confocal laser scanning microscope (Carl Zeiss, Oberkochen, Germany). Syn1 and Psd95 colocalization puncta on mature Map2-positive neurons were identified as synaptic puncta using Imaris software (Bitplane, Oxford Instruments, Zurich, Switzerland). Puncta density was expressed as the number of qualifying puncta per 10 µm of neurite length. 

### Validation of DAS transcripts by high resolution melting qRT-PCR analysis

The RNA sample was converted to cDNA using reverse transcription (Thermo Fisher Scientific, Waltham, MA, USA). Firstly, RNA was quantified using a NanoDrop spectrophotometer to measure concentration and purity by assessing the 260/280 absorbance ratio, and then diluted according to the synthesis kit instructions. After that, RNA was converted into cDNA using the Reverse transcription reaction using RevertAid First Strand cDNA Synthesis Kit (Thermo Fisher Scientific, USA). To validate the alternative splicing pattern of DAS transcripts, high-resolution melting (HRM) qRT-PCR analysis was performed. Primers were designed to span the alternatively spliced exons of *Ccar1* (exon 16) and *Jmjd1c* (exon 5), with primer binding sites mapped to the flanking constitutive exons on the pre-mRNA nucleotide sequence (exons 15–16–17 for *Ccar1*; exons 4–5–6 for *Jmjd1c*). Primers of each PCR reaction were designed using Ensembl (Rnor_6.0 rat reference genome; https://asia.ensembl.org) and Primer3 software (http://primer3.ut.ee/). The predicted primers were validated by in silico PCR using UCSC In Silico PCR (https://genome.ucsc.edu/cgi-bin/hgPcr). The cDNA sample was diluted to yield 1 μg/μl, and HRM qRT-PCR was performed using Precision Melt Supermix (Bio-Rad Laboratories, Hercules, CA, USA) and QuantStudio 6 Real-Time PCR System (Applied Biosystems, Thermo Fisher Scientific, Waltham, MA, USA). Alternative splicing patterns and quantification of each transcript product were calculated and analyzed by Applied Biosystems™ High-Resolution Melting Software v3.0.2 (Applied Biosystems, Thermo Fisher Scientific). Isoform abundance was quantified using the percentage peak height derived from the HRM derivative melt curve, which reflects the relative fluorescence contribution of each amplicon at its characteristic melting temperature (Tm). The inclusion-to-exclusion count ratio (IC/EC ratio) was then calculated as the percentage peak height of the inclusion isoform divided by that of the exclusion isoform, providing an internally normalized measure of splicing balance for each sample. 

### Statistical analyses

Statistical analyses were performed using SPSS version 29.0 (SPSS Inc., Chicago, IL, United States). A two-tailed Student’s t-test was used to determine the difference between the means of two groups. For the synaptogenesis assay, statistical inference was performed at the neurite level (n = 72 neurites per condition), with litter means (n = 3 litters per condition) displayed descriptively. Cohen’s d values were calculated at the neurite level. Because formal sex × treatment interaction analyses were not performed, sex-related conclusions are presented as sex-dependent or sex-stratified rather than interaction-based effects. Differential alternative splicing was identified using rMATS (v4.1.2) with a false discovery rate (FDR) < 0.05 and |ΔPSI|≥ 0.1 as significance thresholds. FDR correction was applied using the Benjamini–Hochberg procedure. Gene set enrichment analysis was performed using fGSEA with 1,000 permutations and a significance threshold of padj < 0.05. Over-representation analysis was conducted using EnrichR against multiple gene set databases, with FDR < 0.10 as the significance threshold. RBP binding site enrichment at differentially alternatively spliced exons was assessed by a one-tailed permutation test (n = 10,000 permutations) comparing observed binding site counts at the DAS exon against a null distribution derived from non-DAS skipped exons of the same gene; statistical significance was defined as p < 0.001.

## Results

### Prenatal BPA exposure alters alternative splicing patterns in the rat offspring cerebellum in a sex-dependent manner

**Fig. 2:**
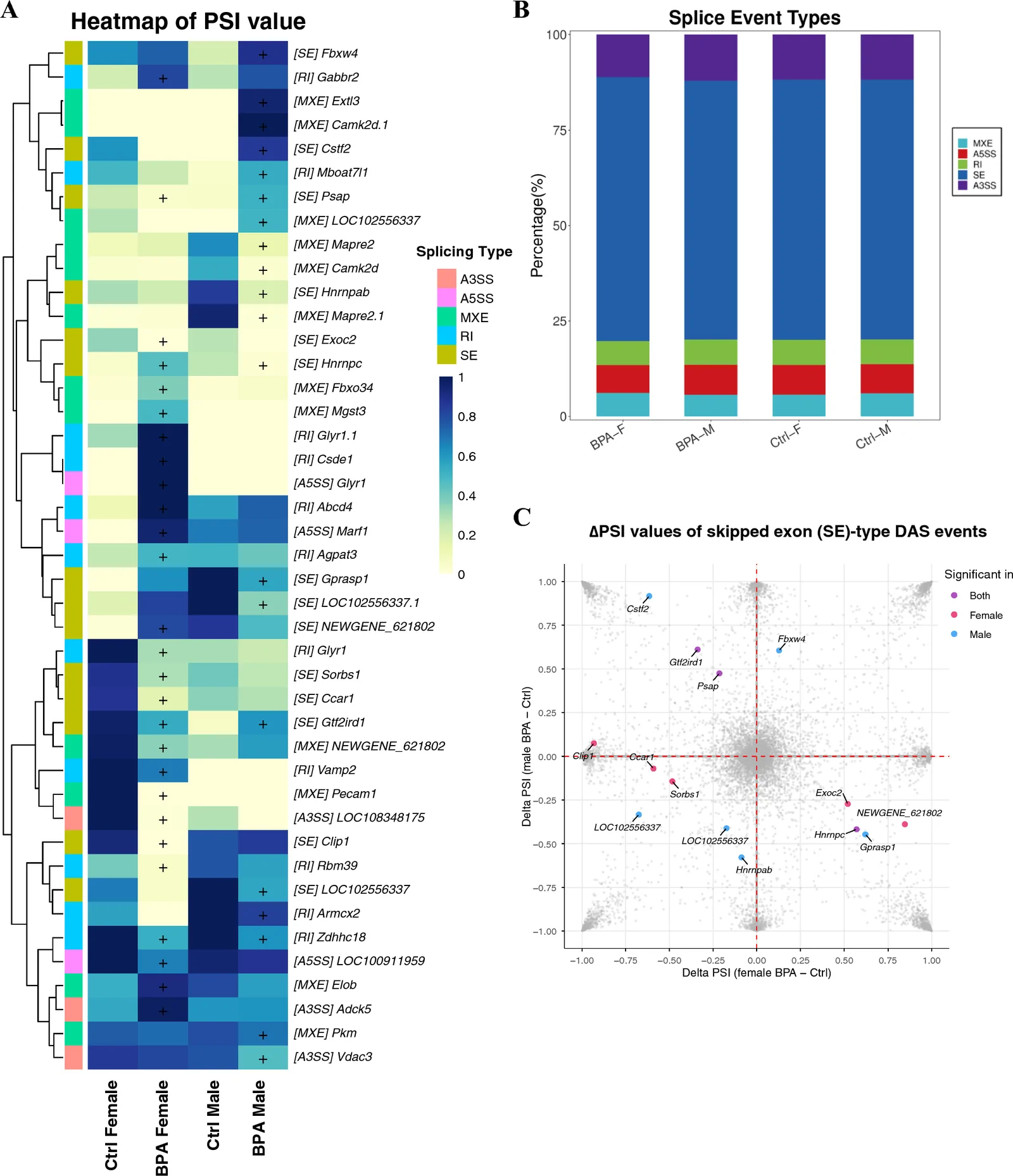

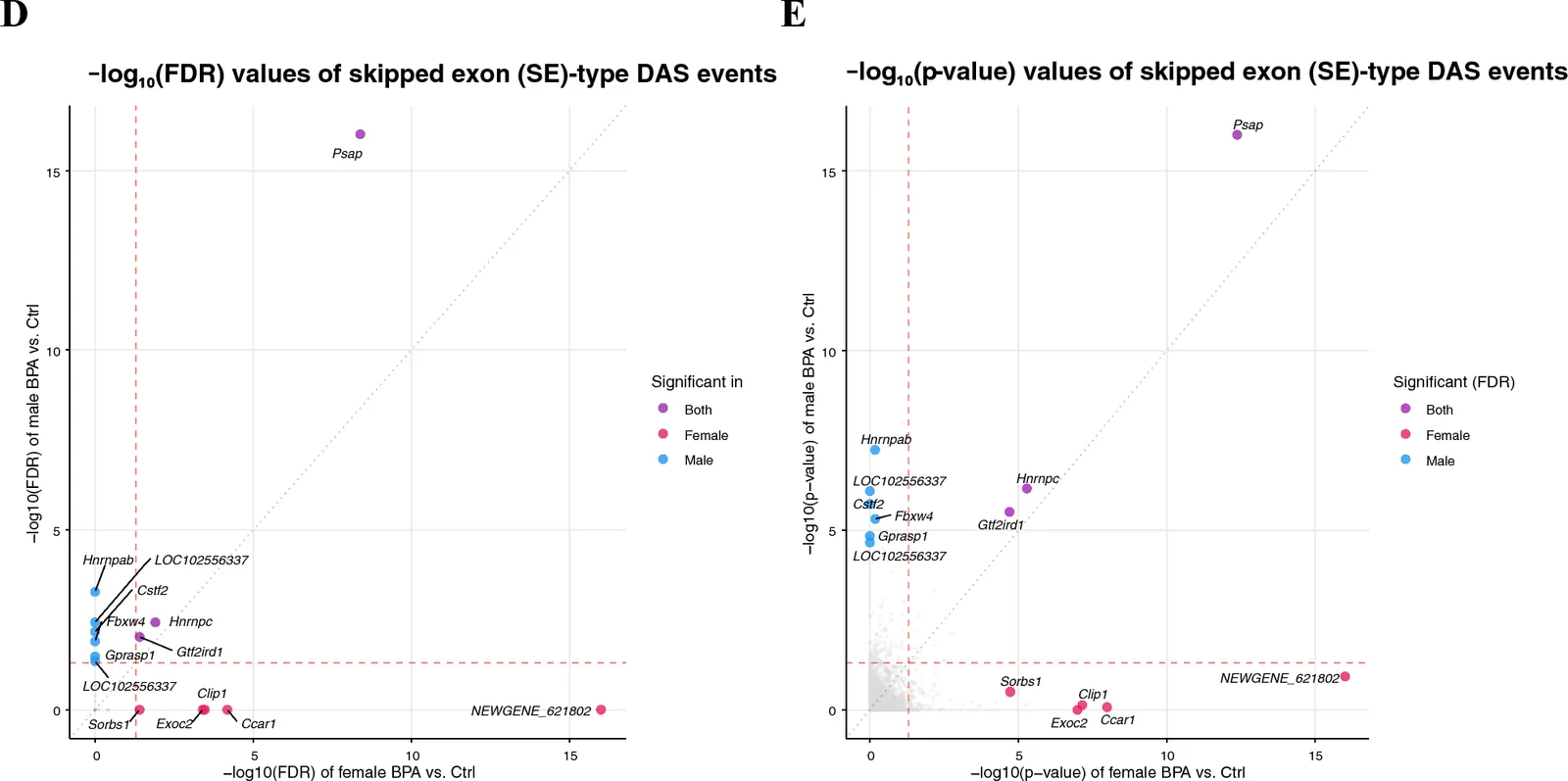
Prenatal BPA exposure is associated with sex-specific differential alternative splicing in the neonatal rat cerebellum. (**A**) Heatmap of percent spliced-in (PSI) values for all significant DAS events (FDR < 0.05) across four experimental groups (Control Female, BPA Female, Control Male, BPA Male; n = 3 per group). Each row represents one splicing event annotated by event type (SE, MXE, RI, A5SS, A3SS; color-coded sidebar). Hierarchical clustering was applied to rows. (+) symbols indicate PSI values that passed the significance threshold. Color scale: 0 (yellow) to 1 (dark blue). (**B**) Stacked bar chart showing the proportion of each alternative splicing event type (SE, MXE, RI, A5SS, A3SS) detected among DAS events in each experimental group. (**C**) Scatter plot of ΔPSI values (PSD BPA − PSI Control) for skipped exon (SE)-type DAS events, comparing male (y-axis) versus female (x-axis) offspring. Colored points indicate genes significant in males only (blue), females only (pink), or both sexes (purple). Gray points represent non-significant events. Labeled genes represent sex-specific DAS candidates discussed in the text. (**D**) Scatter plot of − log₁₀(FDR) values for SE-type events, comparing male versus female analyses. Labeled genes and color coding as in panel C. Genes classified as sex-specific show low statistical signal in the non-designated sex, confirming biological divergence rather than a power artifact. (**E**) Scatter plot of − log₁₀(raw p-value) for SE-type events, male versus female, as a complementary visualization to panel D. Color coding as in panels C–D

To investigate and compare the effects of prenatal bisphenol A exposure on alternative splicing events in the cerebellum of male and female rat offspring, RNA-seq analysis of the postnatal day 1 rat cerebellum was performed. The cerebellum was isolated from both male and female rat pups that were prenatally exposed to BPA at a concentration of 5,000 µg/kg·maternal body weight, which corresponds to a historical no-observed-adverse-effect level (NOAEL) used in earlier regulatory contexts [74] or vehicle control daily by oral gavage from the gestational day (GD) 1 until the parturition day. The selected dose was based on prior pharmacokinetic evidence and its historical use as a regulatory NOAEL in developmental BPA studies [75, 76]. To investigate sex differences in the effects of prenatal BPA exposure, RNA-seq data from male and female pups were analyzed independently. RNA-seq analysis detected the alternative splicing events in neonatal rat cerebellum: 20,154 events in male control, 19,496 events in male BPA, 19,616 events in female control, and 20,692 events in female BPA, respectively. In addition, we observed distinct splicing patterns across the treatment groups, quantified by the difference in percent spliced-in (ΔPSI, ΔΨ). This metric reflects changes in the inclusion read ratio, with positive values indicating increased exon inclusion in BPA-treated samples relative to controls. We found 105 transcripts that exhibited significant differential alternative splicing in BPA-treated offspring compared to controls, representing 15 and 25 DAS genes identified in males and females, respectively, along with 75 genes detected in the combined dataset (p-value < 0.05, FDR < 0.05, |ΔPSI|≥ 0.10) in the cerebellum, as shown in Table 1. The PSI values for all significant DAS events across the four experimental groups are shown in Fig. 2A, which reveals distinct clustering patterns between the BPA-exposed and control groups in both sexes. Among the five categories of splicing events detected, skipped exon (SE) events constituted the largest proportion of DAS events in all groups, followed by mutually exclusive exon (MXE) and retained intron (RI) events (Fig. 2B).

**Table 1:** Differentially alternative spliced (DAS) genes in cerebellum tissue of rat offspring

Male					
Splicing type	Gene	Gene name	ΔΨ	p-value	FDR
A3SS	*Vdac3*	Voltage-dependent anion channel 3	-0.31	6.97E-11	1.84E-07
MXE	*Mapre2*	Microtubule-associated protein, RP/EB family, member 2	-0.93	< 1.00E-17	< 1.00E-17
	*Camk2d*	Calcium/calmodulin-dependent protein kinase II delta	-0.53	2.93E-11	3.00E-08
	*Mapre2*	Microtubule-associated protein, RP/EB family, member 2	-0.50	2.99E-09	2.04E-06
	*Camk2d*	Calcium/calmodulin-dependent protein kinase II delta	1.00	1.56E-07	8.00E-05
	*LOC102556337*	Mitochondrial fission factor-like	-0.33	9.28E-07	3.80E-04
	*Camk2d*	Calcium/calmodulin-dependent protein kinase II delta	1.00	3.30E-06	1.12E-03
	*Extl3*	Exostosin-like glycosyltransferase 3	0.95	7.60E-06	2.22E-03
RI	*Mboat7l1*	Membrane bound O-acyltransferase domain containing 7-like 1	0.49	2.77E-08	3.94E-05
	*Zdhhc18*	Zinc finger DHHC-type palmitoyltransferase 18	-0.39	1.76E-07	1.26E-04
	*Armcx2*	Armadillo repeat containing, X-linked 2	-0.16	2.20E-05	1.05E-02
SE	*Psap*	Prosaposin	0.47	< 1.00E-17	< 1.00E-17
	*Hnrnpab*	Heterogeneous nuclear ribonucleoprotein A/B	-0.58	5.77E-08	5.35E-04
	*Hnrnpc*	Heterogeneous nuclear ribonucleoprotein C	-0.42	6.91E-07	3.78E-03
	*LOC102556337*	Mitochondrial fission factor-like	-0.33	8.15E-07	3.78E-03
	*Cstf2*	Cleavage stimulation factor subunit 2	0.92	1.87E-06	6.91E-03
	*Gtf2ird1*	GTF2I repeat domain containing 1	0.61	3.10E-06	9.57E-03
	*Fbxw4*	F-box and WD repeat domain containing 4	0.61	4.86E-06	1.29E-02
	*Gprasp1*	G protein-coupled receptor associated sorting protein 1	-0.45	1.46E-05	3.38E-02
	*LOC102556337*	Mitochondrial fission factor-like	-0.41	2.22E-05	4.57E-02

Female					
splicing type	Gene	Gene name	ΔΨ	p-value	FDR
A3SS	*Adck5*	aarF domain containing kinase 5	0.41	3.03E-05	4.52E-02
	*LOC108348175*	Protein quaking-like	-0.98	3.44E-05	4.52E-02
A5SS	*Glyr1*	Glyoxylate reductase 1 homolog	1.00	< 1.00E-17	< 1.00E-17
	*LOC100911959*	Ubiquitin carboxyl-terminal hydrolase 5-like	-0.23	1.66E-05	1.47E-02
	*Marf1*	Meiosis regulator and mRNA stability factor 1	0.96	2.83E-05	1.66E-02
MXE	*NEWGENE_621802*	Calcium/calmodulin-dependent protein kinase II gamma	-0.67	< 1.00E-17	< 1.00E-17
	*Mgst3*	Microsomal glutathione S-transferase 3	-0.37	5.74E-12	6.05E-09
	*Pecam1*	Platelet and endothelial cell adhesion molecule 1	-1.00	2.71E-11	1.91E-08
	*Elob*	Elongin B	0.39	3.52E-06	1.86E-03
	*Fbxo34*	F-box protein 34	0.27	3.28E-05	1.39E-02
RI	*Glyr1*	Glyoxylate reductase 1 homolog	0.63	7.77E-16	5.60E-13
	*Glyr1*	Glyoxylate reductase 1 homolog	-0.64	4.44E-16	5.60E-13
	*Zdhhc18*	Zinc finger DHHC-type palmitoyltransferase 18	-0.47	3.76E-12	1.80E-09
	*Vamp2*	Vesicle-associated membrane protein 2	-0.28	2.67E-09	9.61E-07
	*Abcd4*	ATP binding cassette subfamily D member 4	0.79	6.96E-06	2.01E-03
	*Rbm39*	RNA binding motif protein 39	-0.42	1.76E-05	4.24E-03
	*Gabbr2*	Gamma-aminobutyric acid type B receptor subunit 2	0.59	5.89E-05	1.21E-02
	*Csde1*	Cold shock domain containing E1	0.92	1.28E-04	2.30E-02
	*Agpat3*	1-acylglycerol-3-phosphate O-acyltransferase 3	0.25	2.94E-04	4.71E-02
SE	*NEWGENE_621802*	Calcium/calmodulin-dependent protein kinase II gamma	0.85	< 1.00E-17	< 1.00E-17
	*Psap*	Prosaposin	-0.21	4.26E-13	4.12E-09
	*Ccar1*	Cell division cycle and apoptosis regulator 1	-0.59	1.04E-08	6.67E-05
	*Clip1*	CAP-GLY domain containing linker protein 1	-0.93	7.15E-08	3.45E-04
	*Exoc2*	Exocyst complex component 2	0.52	1.04E-07	4.00E-04
	*LOC102556337*	Mitochondrial fission factor-like	-0.86	8.74E-07	2.81E-03
	*LOC102556337*	Mitochondrial fission factor-like	-0.91	3.33E-06	9.19E-03
	*Hnrnpc*	Heterogeneous nuclear ribonucleoprotein C	0.57	5.26E-06	1.27E-02
	*Gtf2ird1*	GTF2I repeat domain containing 1	-0.34	2.02E-05	3.89E-02
	*Sorbs1*	Sorbin and SH3 domain containing 1	-0.48	1.92E-05	3.89E-02

Both Sexes					
Splicing type	Gene	Gene name	ΔΨ	p-value	FDR
A3SS	*Arhgap17*	Rho GTPase activating protein 17	0.07	4.96E-10	1.53E-06
	*Tnnt1*	Troponin T1, slow skeletal type	1.00	1.42E-08	2.18E-05
	*Tcf12*	Transcription factor 12	1.00	2.75E-08	2.82E-05
	*Dixdc1*	DIX domain containing 1	-0.75	3.26E-07	2.51E-04
	*Stard13*	StAR-related lipid transfer domain containing 13	-0.67	5.32E-06	3.27E-03
	*Csf3r*	Colony stimulating factor 3 receptor	0.75	1.23E-05	6.30E-03
	*Zfp94*	Zinc finger protein 94	0.37	2.49E-05	1.10E-02
	*Cxcl12*	C-X-C motif chemokine ligand 12	0.02	2.96E-05	1.14E-02
	*Hgs*	Hepatocyte growth factor-regulated tyrosine kinase substrate	0.59	3.74E-05	1.28E-02
A5SS	*Pot1b*	Protection of telomeres 1B	0.36	7.39E-11	1.60E-07
	*Pot1b*	Protection of telomeres 1B	0.71	2.13E-10	2.31E-07
	*Rpap1*	RNA polymerase II associated protein 1	0.32	5.48E-10	3.95E-07
	*Fra10ac1*	FRA10A associated CGG repeat 1	-1.00	4.34E-09	2.35E-06
	*Mtr*	5-methyltetrahydrofolate-homocysteine methyltransferase	-0.42	5.38E-08	2.33E-05
	*LOC100361018*	rCG22048-like	-0.17	1.39E-07	4.31E-05
	*LOC100361018*	rCG22048-like	-0.67	1.26E-07	4.31E-05
	*Kmt2d*	Lysine methyltransferase 2D	0.77	4.49E-07	1.22E-04
	*Ikbkg*	Inhibitor of nuclear factor kappa B kinase regulatory subunit gamma	0.17	1.64E-06	3.93E-04
	*Iqck*	IQ motif containing K	0.62	2.05E-06	4.44E-04
	*Abhd13*	Abhydrolase domain containing 13	-0.61	7.43E-06	1.46E-03
	*Ddx5*	DEAD-box helicase 5	0.32	1.32E-05	2.20E-03
	*Pigx*	Phosphatidylinositol glycan anchor biosynthesis, class X	0.55	1.32E-05	2.20E-03
	*Naa60*	N(alpha)-acetyltransferase 60, NatF catalytic subunit	-0.50	3.93E-05	5.67E-03
	*LOC100911959*	Ubiquitin carboxyl-terminal hydrolase 5-like	-0.18	3.73E-05	5.67E-03
	*Prom1*	Prominin 1	0.29	6.33E-05	8.57E-03
	*Strada*	STE20 related adaptor alpha	-0.39	1.05E-04	1.33E-02
	*Arhgef2*	Rho/Rac guanine nucleotide exchange factor 2	0.03	1.34E-04	1.62E-02
	*Tymp*	Thymidine phosphorylase	0.50	2.56E-04	2.77E-02
	*Cnot7*	CCR4-NOT transcription complex, subunit 7	0.55	2.48E-04	2.77E-02
	*Hexim2*	HEXIM P-TEFb complex subunit 2	0.53	4.79E-04	4.94E-02
MXE	*Tnn*	Tenascin N	0.83	6.88E-07	1.35E-03
	*Cd36*	CD36 molecule	0.59	7.20E-07	1.35E-03
	*Ntng2*	Netrin G2	0.67	1.83E-06	2.29E-03
	*Pcbp3*	Poly(rC) binding protein 3	0.74	9.37E-06	8.44E-03
	*LOC103690020*	Platelet glycoprotein 4-like	0.67	1.12E-05	8.44E-03
	*Dnah9*	Dynein, axonemal, heavy chain 9	0.37	4.81E-05	3.01E-02
RI	*Zdhhc18*	Zinc finger DHHC-type palmitoyltransferase 18	-0.44	< 1.00E-17	< 1.00E-17
	*Arhgap17*	Rho GTPase activating protein 17	0.05	1.70E-10	1.39E-07
	*Fundc2*	FUN14 domain containing 2	0.65	3.76E-09	2.05E-06
	*Gabbr2*	Gamma-aminobutyric acid type B receptor subunit 2	0.55	3.24E-08	1.32E-05
	*Ormdl1*	ORMDL sphingolipid biosynthesis regulator 1	0.02	1.40E-06	4.57E-04
	*Zscan30*	Zinc finger and SCAN domain containing 30	0.11	2.29E-06	5.35E-04
	*Ikbkg*	Inhibitor of nuclear factor kappa B kinase regulatory subunit gamma	0.15	1.98E-06	5.35E-04
	*Odf2l*	Outer dense fiber of sperm tails 2-like	-0.04	4.88E-06	9.97E-04
	*Fam120b*	Family with sequence similarity 120B	0.12	1.16E-04	2.11E-02
	*Cplane1*	Ciliogenesis and planar polarity effector 1	0.65	2.62E-04	4.28E-02
	*Smarca2*	SWI/SNF related, matrix associated, actin dependent regulator of chromatin, subfamily a, member 2	-0.48	3.38E-04	4.60E-02
	*Slc16a11*	Solute carrier family 16, member 11	-0.18	3.22E-04	4.60E-02
SE	*Mbtd1*	Mbt domain containing 1	-0.41	1.11E-16	3.02E-12
	*LOC100909664*	Centrosomal protein of 170 kDa-like	-0.89	1.90E-14	2.58E-10
	*Exoc2*	Exocyst complex component 2	0.55	1.44E-12	1.30E-08
	*Vash2*	Vasohibin 2	-0.83	2.61E-10	1.78E-06
	*Trim68*	Tripartite motif containing 68	-0.77	1.58E-09	8.58E-06
	*Snip1*	Smad nuclear interacting protein 1	0.75	5.16E-09	2.34E-05
	*Jmjd1c*	Jumonji domain containing 1C	0.67	7.34E-09	2.85E-05
	*Mov10*	Mov10 RISC complex RNA helicase	-0.40	6.62E-08	2.25E-04
	*Rnf41*	Ring finger protein 41	0.15	1.49E-07	4.51E-04
	*Dnaaf4*	Dynein axonemal assembly factor 4	0.67	2.47E-07	6.73E-04
	*Mbtd1*	Mbt domain containing 1	-0.64	3.48E-07	8.61E-04
	*Knop1*	Lysine-rich nucleolar protein 1	0.80	1.03E-06	2.33E-03
	*Mok*	MOK protein kinase	-0.47	1.50E-06	2.92E-03
	*Sorbs1*	Sorbin and SH3 domain containing 1	-0.71	1.46E-06	2.92E-03
	*Mbtd1*	Mbt domain containing 1	-0.70	2.35E-06	4.26E-03
	*Rpap1*	RNA polymerase II associated protein 1	0.23	3.21E-06	5.47E-03
	*Adgrg2*	Adhesion G protein-coupled receptor G2	-0.63	3.96E-06	5.73E-03
	*Gls2*	Glutaminase 2	0.68	3.69E-06	5.73E-03
	*Ptpn13*	Protein tyrosine phosphatase, non-receptor type 13	0.15	4.00E-06	5.73E-03
	*Ppcdc*	Phosphopantothenoylcysteine decarboxylase	-0.67	5.85E-06	7.96E-03
	*Pih1d2*	PIH1 domain containing 2	-0.57	8.93E-06	1.16E-02
	*Arvcf*	ARVCF, delta catenin family member	0.63	9.62E-06	1.19E-02
	*Nrcam*	Neuronal cell adhesion molecule	0.22	1.05E-05	1.24E-02
	*Calcrl*	Calcitonin receptor like receptor	-0.34	1.46E-05	1.65E-02
	*Dysf*	Dysferlin	0.52	1.61E-05	1.68E-02
	*Dab1*	DAB adaptor protein 1	0.87	1.55E-05	1.68E-02
	*LOC103690031*	Sterile alpha motif domain-containing protein 9-like	0.58	1.90E-05	1.92E-02
	*Sestd1*	SEC14 and spectrin domain containing 1	-0.33	2.06E-05	2.00E-02
	*Dnaaf4*	Dynein axonemal assembly factor 4	0.40	2.37E-05	2.23E-02
	*Mta1*	Metastasis associated 1	-0.48	2.69E-05	2.44E-02
	*Ubxn2a*	UBX domain protein 2A	-0.27	2.93E-05	2.57E-02
	*Gsn*	Gelsolin	-0.67	3.56E-05	3.02E-02
	*LOC103689941*	Type II inositol 1,4,5-trisphosphate 5-phosphatase-like	-0.83	3.69E-05	3.04E-02
	*Dgkg*	Diacylglycerol kinase, gamma	0.59	5.51E-05	4.41E-02
	*Tgds*	TDP-glucose 4,6-dehydratase	-0.14	6.06E-05	4.71E-02

Alternative splicing event types are abbreviated as follows: A3SS: alternative 3’ splice site, A5SS: alternative 5’ splice site, MXE: mutually exclusive exons, RI: retained intron, SE: skipped exon

The change in the percent spliced in value (ΔPSI, ΔΨ) was calculated as the shift in the ratio of inclusion reads between groups. Positive ΔPSI values represent increased exon inclusion in the BPA-treated samples relative to controls, whereas negative values indicate increased exon skipping

The figures in this table were created in https://BioRender.com

To assess whether the sex-stratified DAS findings reflected divergent effect patterns rather than only differences in statistical significance, Male ΔPSI and − log₁₀(FDR) values were plotted against their female counterparts for all detectable SE events (Fig. 2C and D). Female-specific DAS genes showed ΔPSI values near zero in males, alongside low − log₁₀(FDR) values, confirming the absence of splicing change in males rather than an equivalent trend below the significance threshold. The results indicate that genes classified as female-biased DAS candidates (*Ccar1*, *Sorbs1*, *Clip1*, NEWGENE 621802, *Exoc2*) fall near the y = 0 axis in the ΔPSI plot, indicating minimal splicing change in males, and near the x-axis in the − log₁₀(FDR) plot. Similarly, male-specific DAS genes (*Fbxw4*, *Hnrnpab*, *Cstf2*, *Gprasp1*) showed near-zero ΔPSI and were not significant in females. Moreover, genes significant in the combined-sexes analysis displayed sex-divergent ΔPSI directionality in several cases. For example, *Psap* showed exon skipping in females (ΔPSI =  − 0.21) but exon inclusion in males (ΔPSI =  + 0.47). Additionally, similar patterns were observed in the − log₁₀(p-value) of Male ΔPSI versus Female ΔPSI (Fig. 2E), suggesting that BPA may induce sex-opposite splicing regulation at DAS events. Together, these data demonstrate that the sex-specific DAS classifications in the cerebellum following BPA exposure reflect biological sex divergence rather than differential statistical power between groups.

### Prenatal BPA exposure altered the alternative splicing pattern of the *Ccar1* gene in male neonatal rat cerebellum

**Fig. 3:**
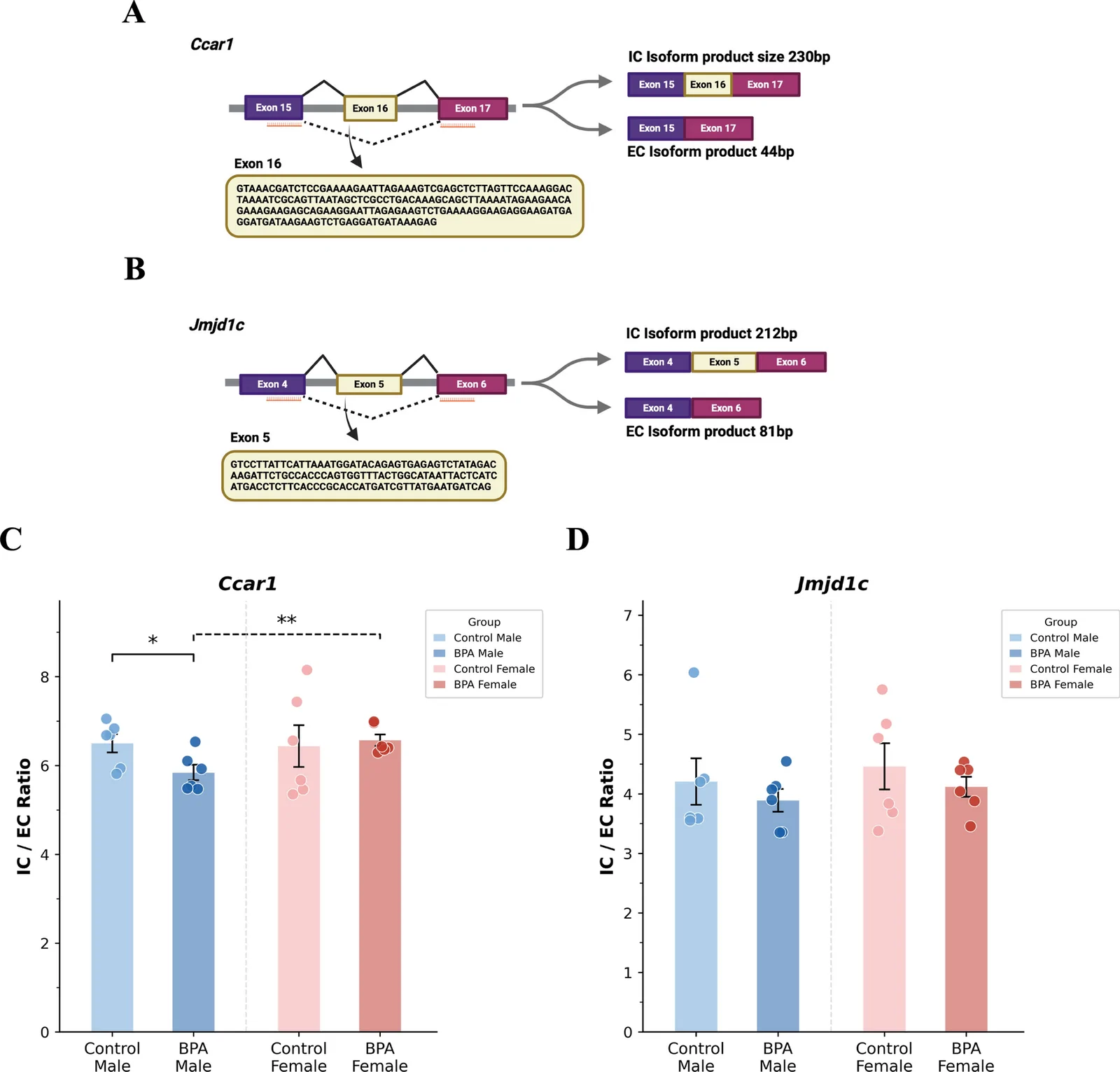
Validation of BPA-associated alternative splicing of *Ccar1* and *Jmjd1c* in the neonatal rat cerebellum by high-resolution melting (HRM) qRT-PCR. Schematic illustrations of the HRM-qRT-PCR primer design for the detection of alternative splicing of *Ccar1* exon 16 (**A**) and *Jmjd1c* exon 5 (**B**), created in https://BioRender.com. Boxes represent exons; connecting lines represent introns. Forward (F) and reverse (R) primers are mapped to the flanking constitutive exons on the pre-mRNA nucleotide sequence. Primer pairs flanking the alternative exon produce an inclusion isoform and an exon-skipped exclusion isoform, with respective product sizes as indicated (230 bp and 44 bp for *Ccar1*; 212 bp and 81 bp for *Jmjd1c*). Relative isoform abundance of *Ccar1* (**C**) and *Jmjd1c* (**D**) expressed as the inclusion-to-exclusion count ratio (IC/EC ratio) from HRM peak-height analysis. Groups are shown for the combined-sexes analysis (Control and BPA, regardless of sex), followed by sex-stratified comparisons (Control Male, BPA Male, Control Female, BPA Female). Data are presented as mean ± SEM with individual data points (n = 6 biological replicates per group; each replicate represents one individual pup from an independent litter). * p < 0.05; ** p < 0.01 (Student’s t-test). Dashed bracket indicates between-sex comparison within the same treatment condition. IC, inclusion count; EC, exclusion count

To further validate BPA effects on alternative splicing events in the neonatal rat cerebellum, the splicing events of selected BPA-responsive DAS genes from RNA-seq analysis (p-value < 0.05 and FDR < 0.05) were validated using high-resolution melting (HRM) qRT-PCR analysis with RNA samples from cerebellum tissue (Fig. 3), including *Sorbs1* (Sorbin and SH3 domain containing 1), *Jmjd1c* (Jumonji Domain Containing 1C), *Hnrnpab* (Heterogeneous Nuclear Ribonucleoprotein A/B), *Gtf2ird1* (GTF2I repeat domain containing 1), *Dnaaf4* (Dynein Axonemal Assembly Factor 4), and *Ccar1* (Cell division cycle and apoptosis regulator 1). These genes were selected because they are either ASD candidate genes or involved in developmental disorders [77, 78]. Among these selected DAS genes, *Jmjd1c* has been identified as an autism candidate gene in the SFARI database, with a ranking score of 2. Primers were designed to span the alternatively spliced exon in each target gene, with binding sites mapped to the flanking constitutive exons on the pre-mRNA nucleotide sequence and were validated using in silico PCR in the UCSC Genome Browser.

The exon-skipping events for *Ccar1* (exon 16) and *Jmjd1c* (exon 5) are illustrated schematically in Fig. 3A and Fig. 3B, respectively, with primer binding sites indicated on the pre-mRNA nucleotide sequence. From HRM qRT-PCR analysis, the analysis of *Ccar1* revealed two distinct melting temperature (Tm) peaks, including Tm = 69 °C (exclusion transcript) and Tm = 79 °C (inclusion transcript). To quantify relative isoform abundance, isoform expression was expressed as the inclusion-to-exclusion count ratio (IC/EC ratio) from HRM peak height analysis (Fig. 3C and D). The IC/EC ratio was significantly lower in BPA-exposed males compared to control males (Control Male: 6.50 ± 0.51; BPA Male: 5.84 ± 0.43; p-value = 0.035), indicating a relative increase in the exon-skipped transcript in the male BPA group compared to the vehicle control. No significant difference in the IC/EC ratio was observed in the combined-sexes analysis (p-value = 0.373) or in the female-only comparison (p-value = 0.791), further supporting a male-biased or sex-dependent effect of BPA on *Ccar1* exon 16 splicing. The HRM qRT-PCR analysis of *Jmjd1c* also revealed two distinct Tm products, with Tm values of 77 °C (exclusion transcript) and 80 °C (inclusion transcript). The IC/EC ratio of *Jmjd1c* showed no significant differences in the combined-sexes (p-value = 0.275), male-only (p-value = 0.485), or female-only (p-value = 0.438) comparisons (Fig. 3D). An increasing trend in the exclusion transcript product of *Jmjd1c* was observed in the BPA-treated male and combined-sexes analysis compared to the vehicle control, though this did not reach statistical significance. We further investigated whether the observed DAS genes were associated with changes in overall gene abundance using our RNA-seq data. Notably, the total mRNA expression levels of *Ccar1* and *Jmjd1c* did not show significant differences between the BPA-exposed and control groups in either male or female offspring (all FDR > 0.05; Additional file 4). These data are consistent with the interpretation that prenatal BPA affects these targets primarily at the level of alternative splicing rather than total transcript abundance.

The alternative splicing products of *Ccar1* and *Jmjd1c* were confirmed in the neonatal rat cerebellum using gel electrophoresis. PCR products of HRM qRT-PCR of *Ccar1* revealed two distinct bands at 230 bp and 44 bp. Sanger sequencing was performed to further validate the 230 bp *Ccar1* product and to confirm its nucleotide sequence as a *Ccar1* isoform in the Rnor6.0 reference genome; the 44 bp product was too short to be sequenced by Sanger sequencing (Supplementary Fig. 2A–E). The spliced exon (Exon 16) of *Ccar1* was further identified using the InterPro Scan bioinformatic tool (Supplementary Fig. 1 K), which revealed that this exonic region encodes the SAP domain, a DNA-binding motif involved in chromatin organization and transcriptional regulation [79]. The gel electrophoresis of *Jmjd1c* revealed two distinct PCR products at 212 bp and 81 bp, validated by Sanger sequencing as *Jmjd1c* isoforms (Supplementary Fig. 2F–J). The specificity of HRM melt peaks for cDNA-containing samples was confirmed by the NTC, which produced no detectable melt curve (Supplementary Fig. 2B and G). These findings suggest that prenatal BPA exposure is associated with altered splicing of *Ccar1*, specifically at the spliced exon that encodes the SAP domain, in the neonatal rat cerebellum, particularly in males.

### Integration of motif prediction and molecular docking analysis identifies candidate RBPs and predicts favorable BPA–RBP interactions

**Fig. 4:**
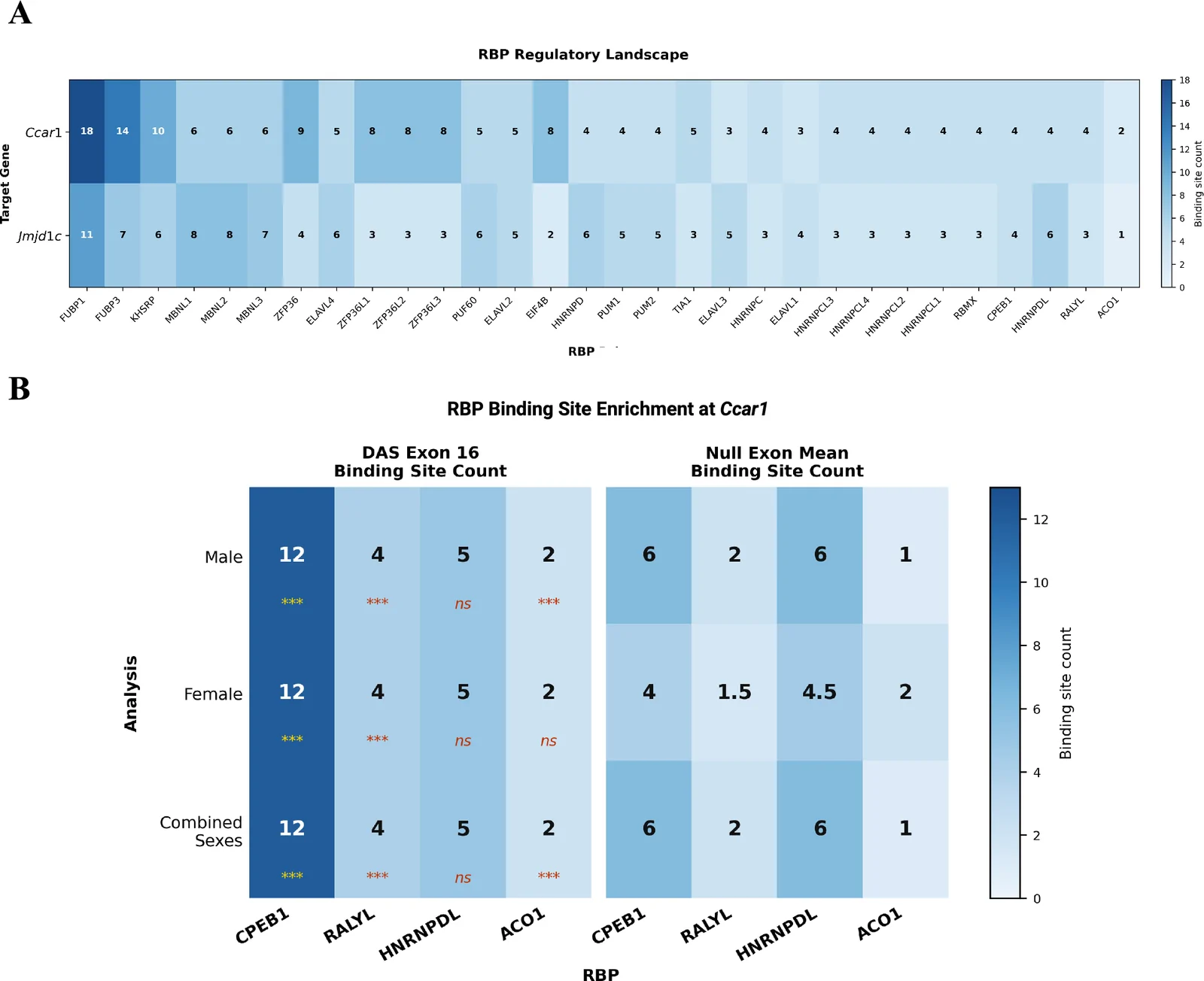
In silico RBP motif enrichment analysis identifies candidate RNA-binding proteins at BPA-responsive alternatively spliced exons. (**A**) Heatmap of predicted RNA-binding protein (RBP) binding site counts within ± 300 bp flanking regions of *Ccar1* exon 16 and *Jmjd1c* exon 5, based on motif scanning against the CisBP-RNA and ATtRACT databases. Color intensity reflects the number of predicted binding sites per RBP per target gene. (**B**) Within-gene permutation analysis of binding site enrichment for four candidate RBPs (CPEB1, RALYL, HNRNPDL, ACO1) at *Ccar1* exon 16 (DAS exon; left panel) compared to null exon windows (non-DAS *Ccar1* skipped exons with FDR > 0.50; right panel), shown separately for male, female, and combined-sexes rMATS analyses. Numbers indicate binding site counts. Statistical significance was assessed by one-tailed permutation test; ***p < 0.001, ns not significant

To investigate whether prenatal BPA exposure could dysregulate alternative splicing of the DAS gene in the neonatal rat offspring, we performed a targeted RNA-binding protein (RBP) motif enrichment analysis using a Python-based motif scanning. From our RNA-seq data, the alternatively spliced exons of *Ccar1* (exon 16) and *Jmjd1c* (exon 5) were selected for in-depth regulatory mapping. We extracted the genomic sequences of these exons along with 300 bp of the upstream and downstream intronic flanking regions to predict the RNA-binding landscapes using the CisBP-RNA database. The motif scanning analysis identified predicted binding sites for splicing factors, including CPEB1, RALYL, HNRNPDL, and ACO1, within the ± 300 bp flanking regions of *Ccar1* exon 16 and Jmjd1c exon 5 (Fig. 4A). To statistically evaluate whether these binding sites were enriched at the DAS exon relative to other *Ccar1* exons detectable in the same experimental context, we performed within-gene permutation testing using all *Ccar1* skipped exon events with FDR > 0.50 in each sex-stratified rMATS analysis as null windows (male: n = 1; female: n = 2; combined-sex: n = 1 null exon), applying the identical ± 300 bp motif scanning algorithm to each. CPEB1 and RALYL binding site densities at *Ccar1* exon 16 were significantly greater than the null exon windows across all three analyses (all p < 0.001), while ACO1 showed significant enrichment in the male and combined-sexes analyses (Fig. 4B). HNRNPDL was not significantly enriched in any analysis, consistent with its site count being comparable to the null exon in each group (Additional file 6). These findings provide statistical support for the selective enrichment of CPEB1 and RALYL regulatory elements at the BPA-responsive splicing event, though direct experimental validation via CLIP-seq would be required to establish binding specificity in the developing rat cerebellum.

**Fig. 5:**
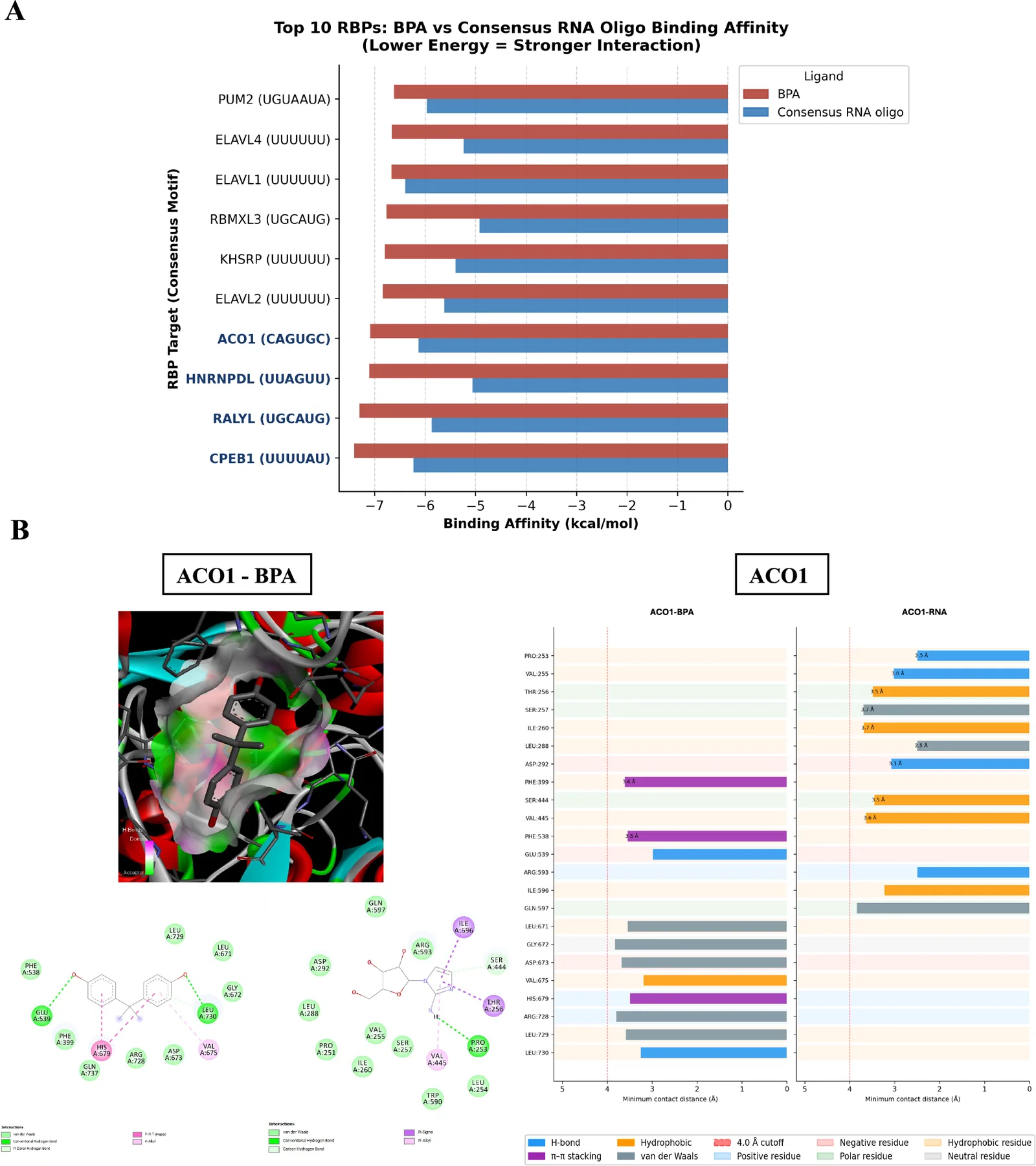

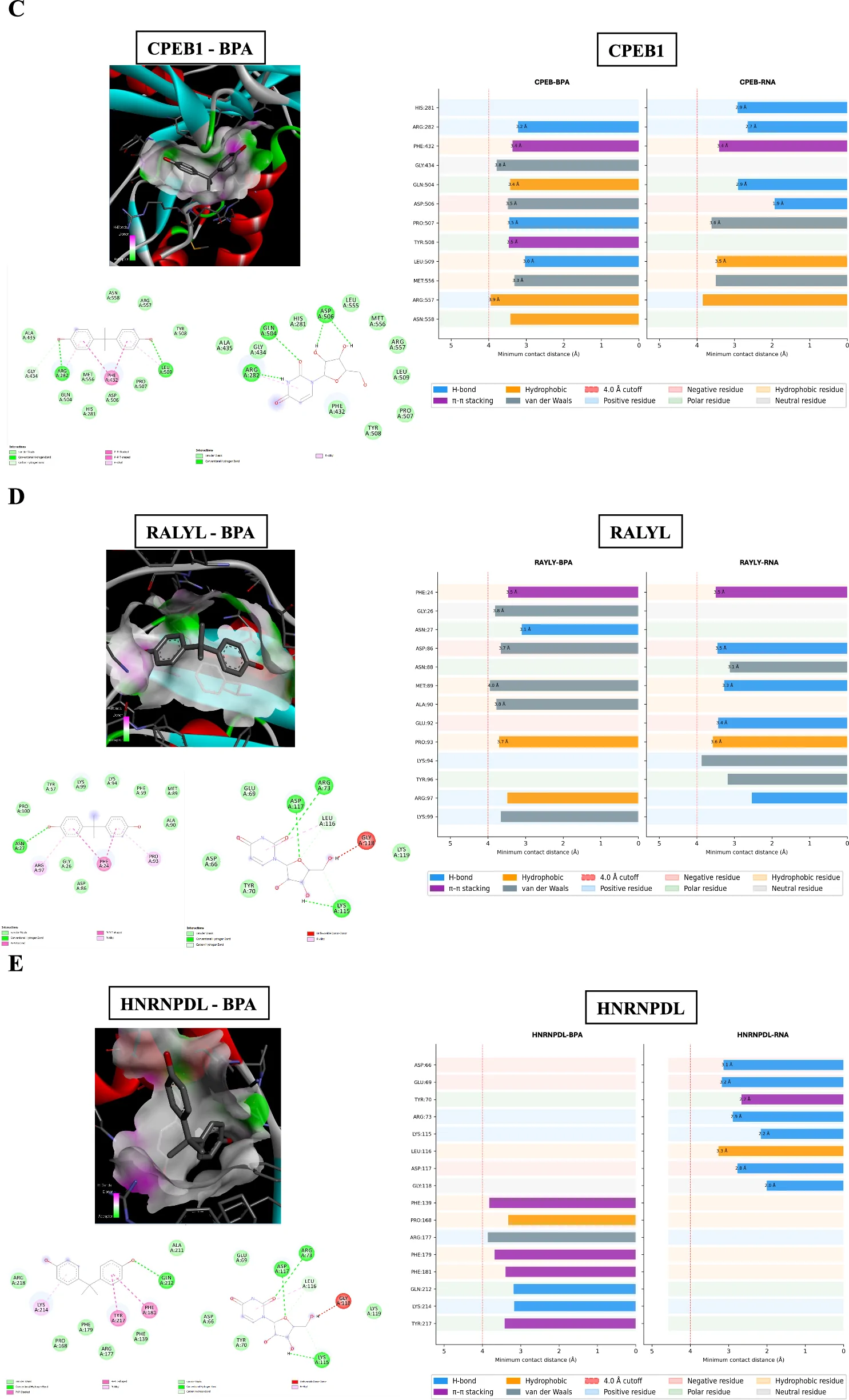
Molecular docking analysis predicts favorable BPA binding at the RNA-binding interfaces of candidate regulatory RBPs. (**A**) Predicted mean binding affinities (ΔG, kcal/mol) of BPA (red) and the first ribonucleoside of each RBP’s experimentally validated consensus RNA motif (blue; sourced from the ATtRACT database) docked against the top 10 shared RBPs. Lower (more negative) values indicate stronger predicted interactions. BPA demonstrated more favorable predicted binding than the representative RNA nucleoside proxy at the majority of RBPs examined. (**B**–**E**) Detailed docking results for the four candidate RBPs identified from motif enrichment analysis: (**B**) ACO1, (**C**) CPEB1, (**D**) RALYL, and (**E**) HNRNPDL. For each RBP, panels show: (left) the three-dimensional docking pose of BPA within the RBP binding cavity; (center) the two-dimensional protein–ligand interaction diagram for the BPA-docked complex; (right) residue-level minimum contact distance plots comparing the BPA-docked pose (left subplot) and the RNA nucleoside-docked pose (right subplot), with interaction types color-coded as indicated in the legend. The RNA ligand used in competitive docking represents the first ribonucleoside of each RBP’s consensus motif as a proxy for the native RNA substrate; full oligonucleotide docking is not supported by AutoDock Vina

To further investigate whether the observed splicing alterations might involve favorable predicted BPA–protein interactions, we performed molecular docking simulations using AutoDock Vina. We calculated the binding free energy (ΔG) of BPA docked to the full three-dimensional structures of the enriched RBPs and compared it against the binding energies of the consensus RNA motif ribonucleoside for each respective RBP, as shown in Fig. 5. This approach uses the first ribonucleoside of each experimentally validated consensus motif as a simplified RNA proxy, while acknowledging that full oligonucleotide docking is not supported by AutoDock Vina. The molecular docking analysis predicted favorable BPA–RBP interactions across the majority of the 42 shared RBPs examined, with 39 of 42 RBPs (93%) displaying more favorable BPA binding than their respective consensus RNA motif ribonucleoside (ΔΔG < 0; Fig. 5, Additional file 7). Among the four key RBPs identified from the motif enrichment analysis of *Ccar1* exon 16 and *Jmjd1c* exon 5, the largest difference in predicted docking score between BPA and the RNA nucleoside proxy was observed for CPEB1, which displayed a binding free energy of − 8.14 ± 0.52 kcal/mol with BPA, substantially more favorable than its interaction with its consensus RNA motif ribonucleoside (Uridine of UUUUAU; ΔG =  − 6.78 ± 0.43 kcal/mol; Fig. 5C). Similarly, HNRNPDL showed a stronger predicted affinity for BPA (ΔG =  − 7.15 ± 0.06 kcal/mol) than for its consensus motif ribonucleoside (UUAGUU; ΔG =  − 5.14 ± 0.07 kcal/mol; Fig. 5E), RALYL (BPA: − 7.31 ± 0.01 kcal/mol vs. UGCAUG motif: − 6.05 ± 0.25 kcal/mol) and ACO1 (BPA: − 7.12 ± 0.02 kcal/mol vs. CAGUGC motif: − 6.29 ± 0.12 kcal/mol; Fig. 5D and B). Notably, docking analyses also predicted favorable BPA docking scores for several RBPs previously implicated in ASD-related biology, including members of the ELAVL/HuR, MBNL, and PCBP families (Supplementary Fig. 3). Collectively, these docking results suggest that BPA may engage candidate RNA-interacting surfaces on selected RBPs under a simplified single-ribonucleoside proxy model of key splicing regulatory proteins, providing a plausible molecular mechanism by which prenatal BPA exposure could disrupt the alternative splicing of *Ccar1* and *Jmjd1c* in the developing cerebellum of neonatal rats though direct competition with full RNA motifs remains to be validated using nucleic acid-compatible docking methods or experimental binding assays.

### Sex-dependent effects of direct BPA exposure on cerebellar neuron viability

**Fig. 6:**
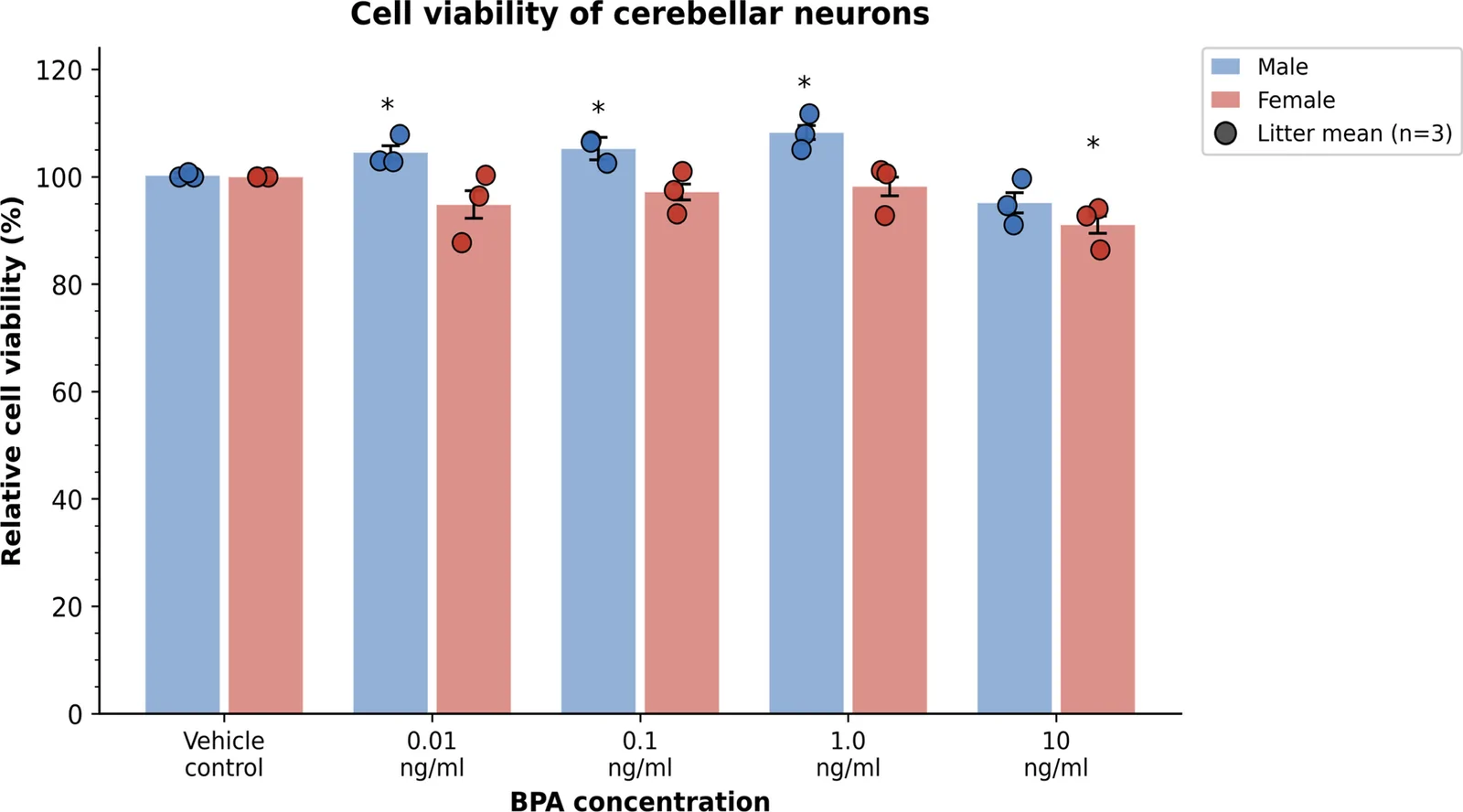
Sex-dependent effects of direct BPA exposure on the viability of primary cerebellar neurons. Relative cell viability (%) of primary cerebellar neurons isolated from male (blue) and female (red) neonatal rat pups and treated with BPA at concentrations of 0.01, 0.1, 1.0, or 10 ng/ml, or vehicle control, for 48 h, assessed by MTS assay. Viability is normalized to the vehicle control within each sex. Bars represent mean ± SEM. Large filled circles indicate litter means (n = 3 independent litters per sex). Asterisks denote significant differences from the within-sex vehicle control (*p < 0.05, Student’s t-test)

Given that *Ccar1* and *Jmjd1c* encode proteins involved in chromatin-related regulation and cell survival, we next investigated whether direct BPA exposure affects the viability of primary cerebellar neurons. The primary cerebellar neurons were isolated from the cerebellar tissues of male and female neonatal rats unexposed to BPA. These neurons were treated with BPA at concentrations of 0.01, 0.1, 1.0, or 10 ng/ml, or with a vehicle control, for 48 h (male: n = 3 litters; female: n = 3 litters). An MTS assay was conducted to assess cell viability, as shown in Fig. 6. The assay revealed different male and female neuronal responses, with BPA exposure significantly increasing the MTS viability signal in male neonatal rats compared with controls. In females, the MTS viability signal did not change significantly at low BPA concentrations relative to the vehicle control. Interestingly, we found that the MTS viability activity signal increased in the male group and modestly increased in a dose-dependent manner when exposed to low concentrations of BPA, ranging from 0.01 ng/ml to 1.0 ng/ml. Meanwhile, in females, viability was significantly lower than in controls when directly exposed to high doses of BPA (P-value < 0.05). This finding suggests that male and female cerebellar neurons may respond differently to direct BPA exposure under these assay conditions.

### Prenatal BPA exposure altered synaptogenesis of primary cerebellar neurons in a sex-dependent manner

**Fig. 7:**
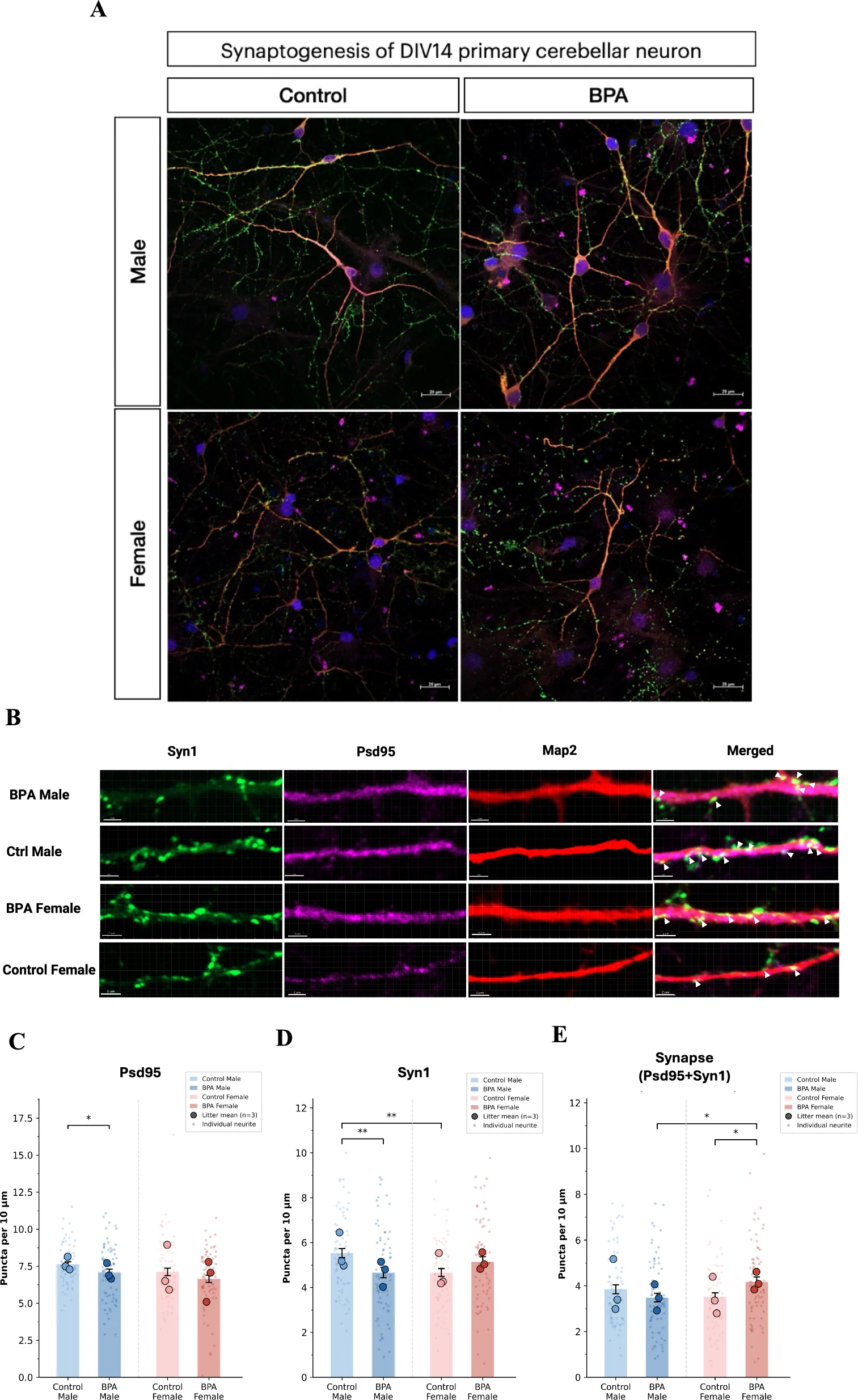
Effect of prenatal BPA exposure on the synaptogenesis of primary cerebellar neurons. (**A**) Representative confocal fluorescence micrographs of DIV14 primary cerebellar neurons from BPA-exposed and vehicle control male and female rat pups. Neurons were immunostained for Map2 (red; neuronal marker), Syn1 (green; presynaptic), and Psd95 (magenta; postsynaptic). Scale bars = 20 µm. (**B**) High-magnification images of representative neurites from each experimental group (BPA Male, Control Male, BPA Female, Control Female), showing individual fluorescence channels (Syn1, Psd95, Map2) and the merged image. Arrowheads indicate sites of Syn1/Psd95 colocalization. Scale bars = 5 µm. (**C**) Psd95 puncta density (puncta per 10 µm neurite length) in male and female groups. BPA-exposed males showed significantly reduced Psd95 density compared to male controls (*p-value= 0.043, Cohen’s d = 0.34). (**D**) Syn1 puncta density. BPA-exposed males showed significantly reduced Syn1 density compared to male controls (**p-value = 0.005, Cohen’s d = 0.48). A significant sex difference in Syn1 density was also observed between Control Male and Control Female neurons (**p-value = 0.001). (**E**) Synaptic puncta density (Syn1/Psd95 colocalization per 10 µm). BPA-exposed females showed significantly increased synaptic puncta density relative to female controls (*p-value = 0.016, Cohen’s d = 0.41). Synapse density was significantly lower in BPA-exposed males than in BPA-exposed females (*p-value = 0.015). For panels **C**–**E**: bars represent group means ± SEM; large filled circles indicate litter means (n = 3 independent litters per sex per condition); small dots indicate individual neurite measurements (n = 72 neurites per condition; 24 neurites per litter). Statistical comparisons by Student’s t-test

To determine whether prenatal BPA exposure could lead to cellular function deficit in cerebellar neurons of the neonatal rat, we assessed synaptogenesis in primary cerebellar neurons derived from prenatally BPA-exposed pups. Synaptogenesis assay of the primary cerebellar neuron was performed by isolation of the cerebellar tissues from independent litters of male and female neonatal rats prenatally exposed to BPA or vehicle control (male = 3 litters and female = 3 litters) and cell culture of the primary cells on poly-L-lysine-coated coverslips for 14 days in vitro (DIV), with each litter serving as one independent biological replicate (n = 3 independent litters per sex per condition). Primary cerebellar neurons were later immunostained against the neuronal mature marker Map2, the presynaptic protein Syn1, and the postsynaptic protein Psd95. Confocal fluorescence microscopy and image analysis were performed to examine colocalization between the presynaptic and postsynaptic puncta, as shown in Fig. 7A. High-magnification images of representative neurites from each condition, with individual fluorescence channels and arrowheads indicating Syn1/Psd95 colocalization points, are shown in Fig. 7B. Syn1 density (Fig. 7C), Psd95 density (Fig. 7D), and Synaptic puncta density (Fig. 7E) per 10 µm neurite length were quantified in male and female groups (n = 72 neurites per condition; 24 neurites per litter; 3 independent litters). The colocalization density of Syn1 and Psd95 puncta (Synapse) was significantly increased in BPA-exposed female neurons relative to female controls (p-value = 0.016, Cohen’s d = 0.41), whereas Synapse density remained unchanged in the male group (p-value = 0.181). BPA-exposed male neurons exhibited significantly reduced Psd95 puncta density (p-value = 0.043, Cohen’s d = 0.34) and Syn1 puncta density (p-value = 0.005, Cohen’s d = 0.48) compared to male controls. Furthermore, the sex difference in Syn1 puncta density was observed between Control Male and Control Female neurons (p-value = 0.001), and Synapse density was significantly lower in BPA-exposed males than in BPA-exposed females (p-value = 0.015), indicating that prenatal BPA exposure is associated with distinct patterns of synaptic puncta density and colocalization in male and female offspring-derived cerebellar neurons.

### Prenatal BPA exposure enriches cerebellar DAS genes for ASD-associated neurodevelopmental pathways and comorbidities

**Fig. 8:**
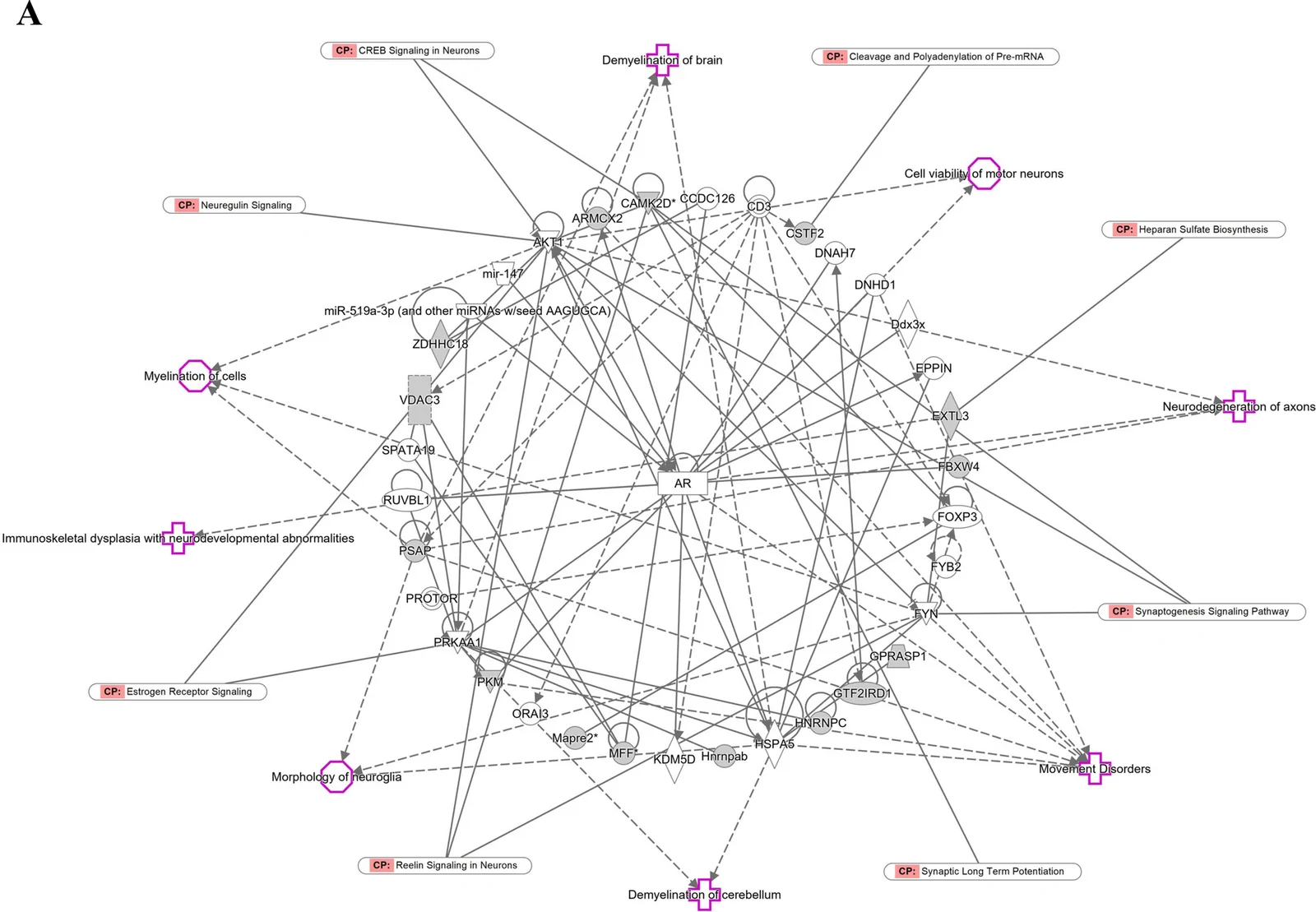

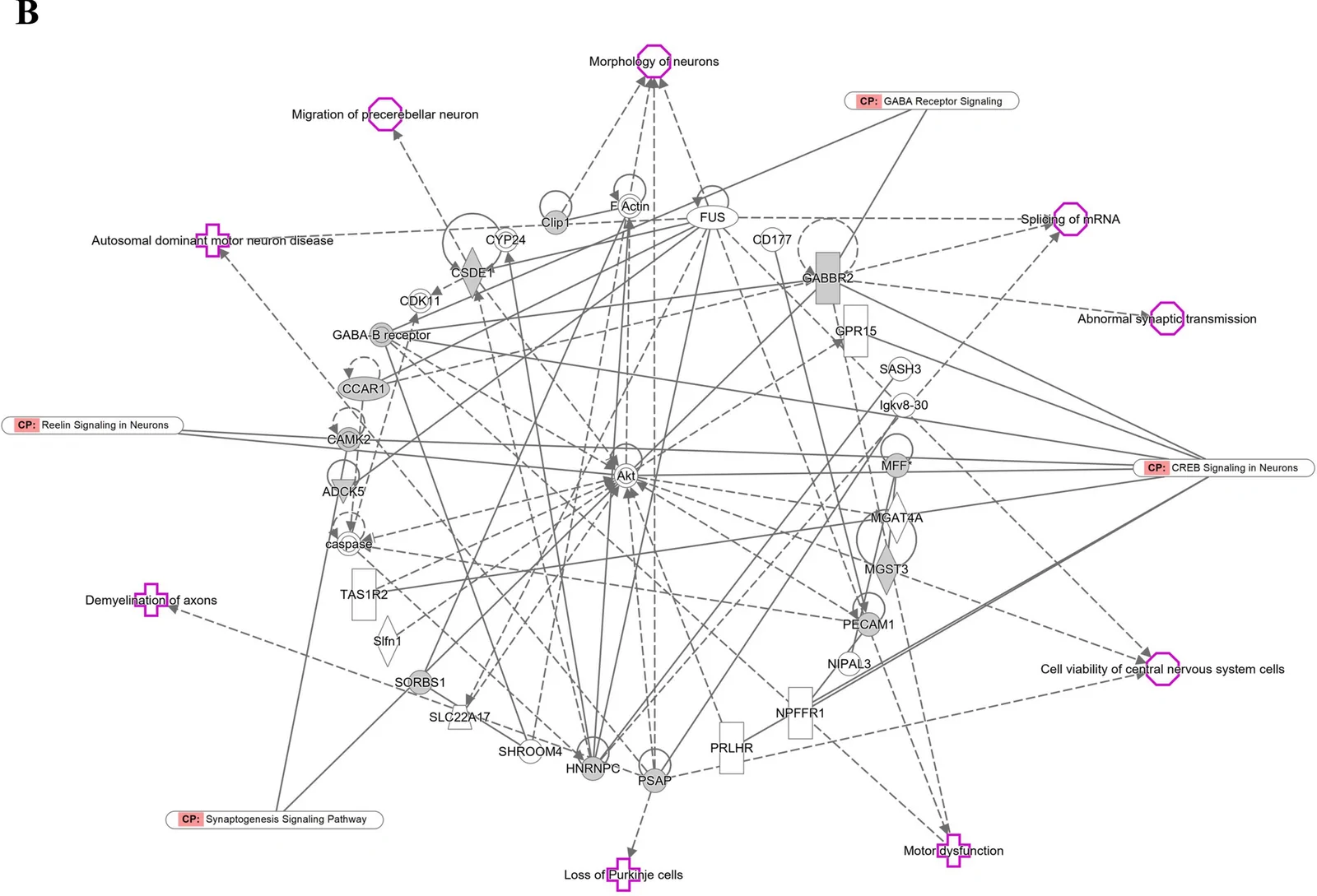

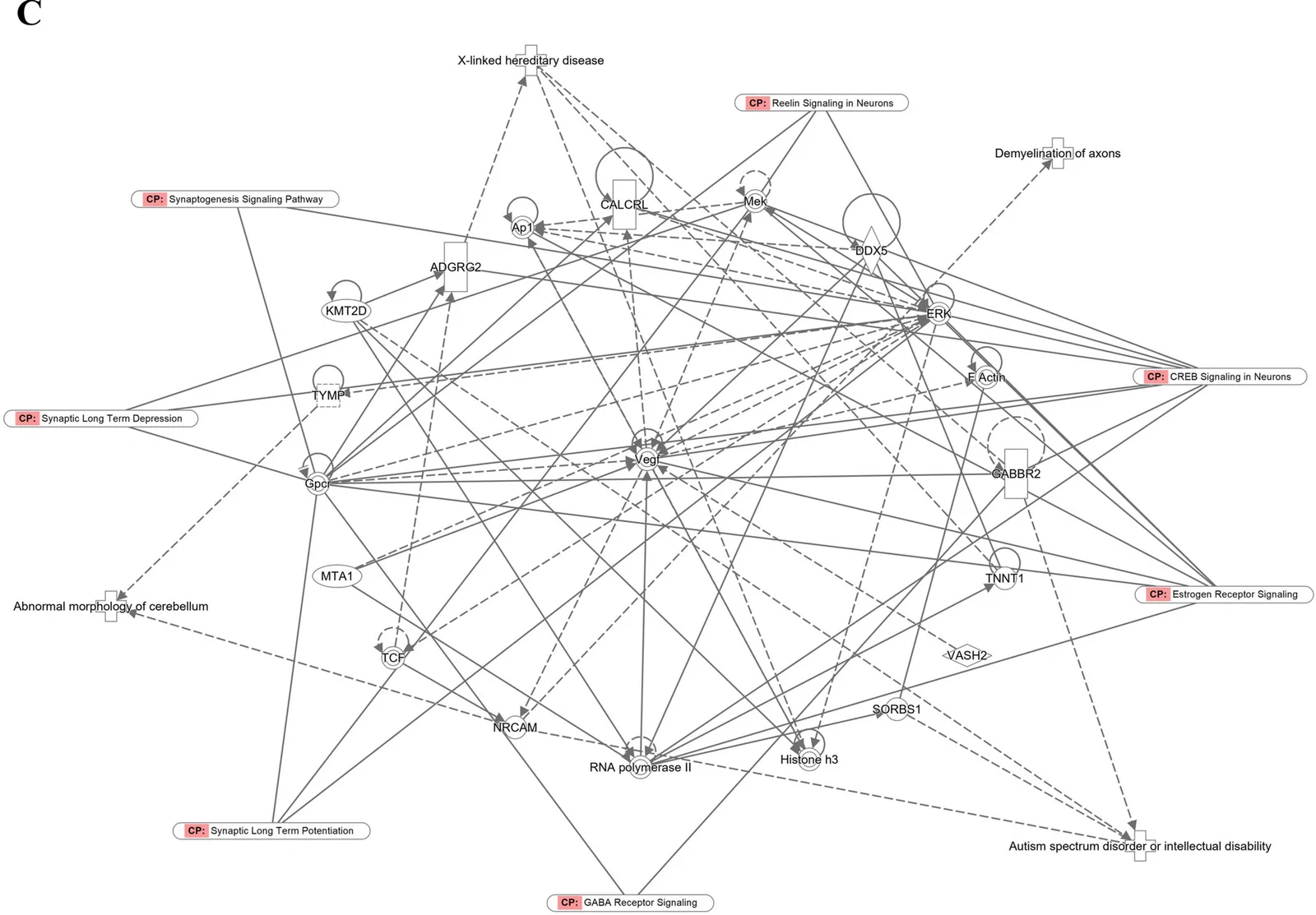
Interactome analysis of the BPA-responsive DAS genes in the neonatal rat cerebellum. The interactome was predicted by IPA software. (**A**) both sexes, (**B**) males, and (**C**) females; Solid lines represent direct interactions (physical binding or enzymatic relationships); dashed lines represent indirect interactions (functional or regulatory relationships inferred from the literature). Node shapes indicate molecule type: circles = genes/proteins; squares = complexes; triangles = kinases; diamonds = enzymes; hexagons = transcription regulators. Pink/magenta-colored nodes and borders indicate molecules identified as differentially alternatively spliced in the current study. Grey nodes represent molecules from the IPA knowledge base connected to the identified DAS genes. Canonical pathways are shown in red-bordered boxes labeled "CP." The color intensity of node borders reflects the degree of connectivity within the network

To predict the biological functions, developmental disorders, and canonical pathways associated with ASD, the identified DAS gene lists were analyzed using Ingenuity Pathway Analysis (IPA) software, and the resulting interactome networks are presented in Fig. 8.

In the combined-sexes analysis, cerebellar DAS genes from prenatally BPA-exposed offspring were significantly associated with multiple neurodevelopmental disorders frequently observed as ASD comorbidities (Table 2; Fig. 8A). Enrichments supported by two or more DAS genes included pervasive developmental disorder (*Gabbr2*, *Gls2*, *Jmjd1c*, *Mtr*, *Nrcam*, *Ntng2*, *Sorbs1*, *Trim68*; p-value = 8.6E-06), epilepsy or neurodevelopmental disorder (p-value = 2.98E-03), pediatric-onset neurological disease (p-value = 8.01E-04), progressive motor neuropathy (p-value= 6.67E-03), and congenital neurological disorder (p-value = 3.81E-03). When analyzed by sex, male cerebellar DAS genes were significantly enriched for neurologically relevant functions involving multiple genes, including fear-conditioning behavior (*Gtf2ird1*, *Vdac3*; p-value = 1.63E-03), abnormal morphology of muscle (*Camk2d*, *Gtf2ird1*, *Psap*, *Vdac3*; p-value = 1.17E-04), and neuromuscular disease (*Armcx2*, *Mff*, *Pkm*, *Psap*; p-value = 0.020; Fig. 8B). Notably, AR emerged as a central hub in the male-specific interactome network (Fig. 8B), connecting multiple BPA-responsive DAS genes, including *Gtf2ird1*, *Psap*, *Hnrnpc*, and *Fbxw4*, suggesting that androgen receptor–related pathways may be relevant to the male-biased splicing patterns of the DAS events observed. Female cerebellar DAS genes showed the most significant enrichments for hyperactive behavior (*Gabbr2*, *Gtf2ird1*, *Psap*, *Vamp2*; p-value = 3.26E-05), pervasive developmental disorder (*Gabbr2*, *Pecam1*, *Sorbs1*, *Vamp2*; n = 4; p-value = 3.22E-04), cerebral palsy (*Gabbr2*, *Vamp2*; p-value = 1.54E-04), anxiety (*Gabbr2*, *Gtf2ird1*, *Vamp2*; p-value = 2.43E-03), and splicing of mRNA (*Ccar1*, *Hnrnpc*; p-value = 0.026; Fig. 8C).

**Table 2:** Neurological disease and function annotations enriched in cerebellar DAS genes from prenatally BPA-exposed rat offspring

Disease or function annotation	p-value	No of genes	DAS genes
**Both Sexes (Combined-sexes Analysis)**			
Pervasive developmental disorder	8.61 × 10⁻⁶	8	*Gabbr2, Gls2, Jmjd1c, Mtr, Nrcam, Ntng2, Sorbs1, Trim68*
Pediatric-onset neurological disease	8.01 × 10⁻^4^	5	*Csf3r, Gabbr2, Snip1, Strada, Tymp*
Early-onset encephalopathy	1.05 × 10⁻^3^	5	*Csf3r, Gabbr2, Jmjd1c, Snip1, Strada*
Early-onset epilepsy	1.38 × 10⁻^3^	4	*Gabbr2, Jmjd1c, Snip1, Strada*
Epilepsy or neurodevelopmental disorder	2.98 × 10⁻^3^	9	*Arhgef2, Calcrl, Gabbr2, Ikbkg, Jmjd1c, Kmt2d, Ntng2, Snip1, Strada*
Childhood encephalopathy	3.07 × 10⁻^3^	4	*Csf3r, Gabbr2, Snip1, Strada*
Demyelination of nervous tissue	3.15 × 10⁻^3^	2	*Ikbkg, Nrcam*
Congenital neurological disorder	3.81 × 10⁻^3^	10	*Arhgef2, Gabbr2, Gsn, Kmt2d, Mtr, Ntng2, Sestd1, Smarca2, Snip1, Tymp*
Progressive motor neuropathy	6.67 × 10⁻^3^	8	*Cd36, Csf3r, Cxcl12, Dab1, Gabbr2, Gsn, Mtr, Nrcam*
Abnormal morphology of granule cell layer	7.12 × 10⁻^3^	2	*Cxcl12, Dab1*
**Male-Specific DAS Gene Enrichment**			
Abnormal morphology of muscle	1.17 × 10⁻^4^	4	*Camk2d, Gtf2ird1, Psap, Vdac3*
Cued conditioning	9.50 × 10⁻^4^	2	*Gtf2ird1, Vdac3*
Fear	1.63 × 10⁻^3^	2	*Gtf2ird1, Vdac3*
Function of neurons	2.88 × 10⁻^3^	2	*Gtf2ird1, Psap*
Quantity of brain cells	2.92 × 10⁻^3^	2	*Camk2d, Psap*
Hyperactive behavior	7.69 × 10⁻^3^	2	*Gtf2ird1, Psap*
Leukoencephalopathy	9.60 × 10⁻^3^	2	*Mff, Psap*
Splicing of mRNA	1.34 × 10⁻^2^	2	*Cstf2, Hnrnpc*
Neuromuscular disease	1.99 × 10⁻^2^	4	*Armcx2, Mff, Pkm, Psap*
Long-term potentiation	2.10 × 10⁻^2^	2	*Psap, Vdac3*
Autosomal recessive neurological disorder	3.44 × 10⁻^2^	3	*Extl3, Mff, Psap*
**Female-Specific DAS Gene Enrichment**			
Hyperactive behavior	3.26 × 10⁻^5^	4	*Gabbr2, Gtf2ird1, Psap, Vamp2*
Cerebral palsy	1.54 × 10⁻^4^	2	*Gabbr2, Vamp2*
Pervasive developmental disorder	3.22 × 10⁻^4^	4	*Gabbr2, Pecam1, Sorbs1, Vamp2*
Muscle spasticity	9.95 × 10⁻^4^	2	*Gabbr2, Vamp2*
Excitatory postsynaptic current	2.36 × 10⁻^3^	2	*Gabbr2, Vamp2*
Anxiety	2.43 × 10⁻^3^	3	*Gabbr2, Gtf2ird1, Vamp2*
Function of neurons	5.64 × 10⁻^3^	2	*Gtf2ird1, Psap*
Action potential of neurons	1.03 × 10⁻^2^	2	*Gabbr2, Vamp2*
Generalized seizures	1.52 × 10⁻^2^	2	*Gabbr2, Psap*
Progressive neuromuscular disease	1.75 × 10⁻^2^	3	*Gabbr2, Psap, Vamp2*
Neuromuscular disease with neuropathy	1.82 × 10⁻^2^	3	*Gabbr2, Psap, Vamp2*
Leukoencephalopathy	1.85 × 10⁻^2^	2	*Mff, Psap*
Quantity of neurons	2.12 × 10⁻^2^	3	*Gabbr2, Psap, Vamp2*
Splicing of mRNA	2.55 × 10⁻^2^	2	*Ccar1, Hnrnpc*
Seizures	3.11 × 10⁻^2^	3	*Gabbr2, Psap, Vamp2*
Long-term potentiation	3.97 × 10⁻^2^	2	*Psap, Vamp2*
Progressive encephalopathy	4.55 × 10⁻^2^	4	*Gabbr2, Pecam1, Psap, Vamp2*
Early-onset neurological disorder	4.69 × 10⁻^2^	2	*Gabbr2, Gtf2ird1*
Syndromic neurodevelopmental disorder	4.93 × 10⁻^2^	2	*Gabbr2, Vamp2*

The table presents a comparison of developmental/neurological disorders between the combined-sexes analysis and males and females, respectively

p-value is calculated using Fisher’s exact test, with a p-value < 0.05 considered statistically significant: DAS: differentially alternatively spliced, IPA: Ingenuity Pathway Analysis

Only enrichments supported by ≥ 2 overlapping DAS genes are shown. Enrichments with a single-gene overlap are provided in additional file 7

To further contextualize these findings, interactome networks were generated by integrating disease annotations and canonical pathways (p < 0.05; Fig. 8). The combined-sexes interactome showed significant associations with autism spectrum disorder or intellectual disability, abnormal morphology of cerebellum, and synaptic long-term potentiation (Fig. 8A). Upon sex stratification, the male cerebellum network was predominantly linked to immunoskeletal dysplasia with neurodevelopmental abnormalities, movement disorders, and demyelination of the cerebellum (Fig. 8B), whereas the female network highlighted abnormal synaptic transmission, motor dysfunction, and loss of Purkinje cells (Fig. 8C), which are consistently reported as ASD comorbidities. Together, these findings indicate that BPA-associated cerebellar DAS genes are enriched in pathways and disease annotations relevant to ASD-related neurodevelopmental, motor, and behavioral phenotypes, as well as their frequently comorbid motor and muscular deficits, with notable sex-divergent enrichment patterns reflecting the sex-specific pattern of the observed splicing alterations.

## Discussion

Bisphenol A (BPA) is an endocrine-disrupting chemical increasingly studied for its potential relevance to neurodevelopmental disorders. The No-Observed-Adverse-Effect Level (NOAEL) for BPA was established at 5,000 µg/kg maternal weight/day [43]. However, growing evidence indicates that BPA at or below this threshold can still lead to adverse effects [44, 80, 81]. These regulatory concerns highlight the need to investigate the precise molecular mechanisms by which BPA exposure perturbs neurodevelopment.

**Fig. 9:**
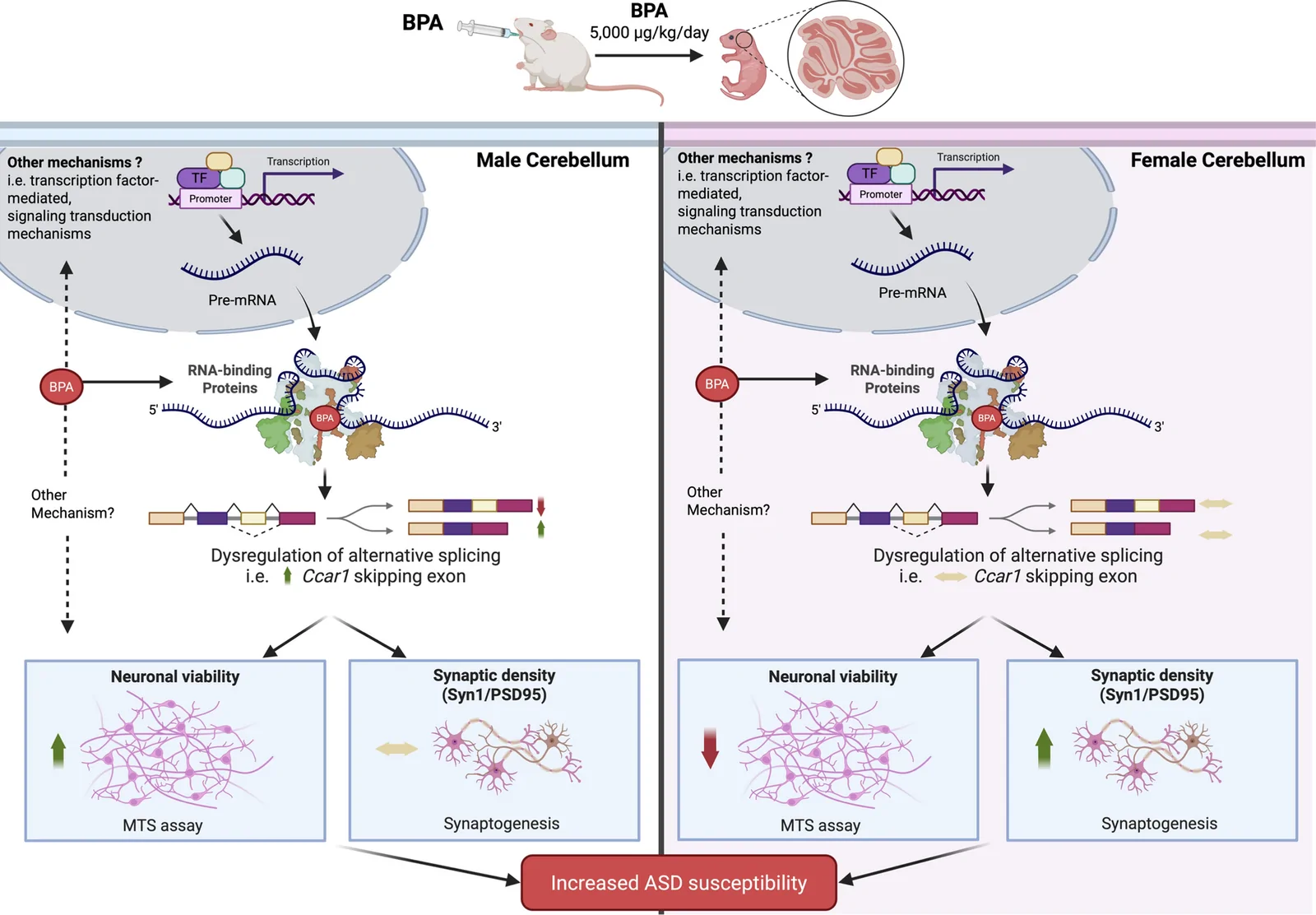
Schematic representation of a hypothetical mechanistic framework proposed based on the current findings. In males, prenatal BPA exposure is proposed to interact with candidate RNA-binding proteins (e.g., Cpeb1, Ralyl, Hnrnpdl, and Aco1), leading to dysregulated alternative splicing of target genes such as *Ccar1,* thereby altering synaptogenesis and neuronal viability outcomes. In females, the molecular mechanisms upstream of the observed phenotypes remain to be determined; the right-hand panel therefore depicts an unknown regulatory pathway that warrants investigation in future studies. Figure created in https://BioRender.com

Our previous works demonstrated that prenatal BPA exposure profoundly alters not only overall transcriptomic profiles but also specific alternative splicing patterns in the prefrontal cortex and hippocampus of rat offspring in a sex-dependent manner that are associated with ASD-related neurological diseases and function and behavioral outcomes [47, 48, 61, 62]. Herein, we propose a hypothetical mechanistic framework (Fig. 9) that connects prenatal BPA exposure to alterations in alternative splicing and subsequent ASD-associated neuronal outcomes via interactions between BPA and candidate RNA-binding proteins (RBPs) in the offspring cerebellum. Using RNA-seq, we examined DAS events in the cerebellum of rat offspring prenatally exposed to BPA. In particular, Our findings suggest that prenatal BPA exposure is associated with altered alternative splicing patterns in the cerebellum, with different patterns observed in males and females. Importantly, scatter plot analyses of ΔPSI and − log₁₀(FDR) values confirmed that genes classified as sex-specific DAS events show genuinely low effect sizes and statistical signal in the non-designated sex, rather than equivalent trends that fell short of the significance threshold due to limited sample size, supporting the interpretation that the sex-stratified classifications are not explained solely by differences in statistical power. We further identified that some of the DAS genes that were significantly associated with ASD candidate genes were also associated with ASD-related neurodevelopmental disorders and neuronal outcomes. This convergence of BPA-associated splicing alterations with ASD-relevant networks supports alternative splicing as a candidate molecular pathway linking prenatal BPA exposure to neurodevelopmental processes relevant to ASD.

Gene ontology and pathway analyses indicated that BPA-associated DAS genes in the rat offspring cerebellum are significantly associated with biological functions and canonical pathways related to neurodevelopmental disorders, particularly ASD (Fig. 8). In the combined-sexes analysis, the most robustly enriched disease annotations, those supported by multiple overlapping DAS genes, included pervasive developmental disorder (8 genes; p-value = 8.60E-06), epilepsy or neurodevelopmental disorder (9 genes; p-value = 2.98E-03), pediatric-onset neurological disease (5 genes; p-value = 8.01E-04) progressive motor neuropathy (8 genes; p-value = 6.67E-03), and congenital neurological disorder (10 genes; p-value = 3.81E-03). These enrichments collectively suggest that prenatal BPA exposure may be relevant to ASD-related cerebellar biology through dysregulation of alternative splicing in genes broadly implicated in neurodevelopmental and motor-related pathways, which are frequently observed as clinical comorbidities of ASD [82–85]. Complementary EnrichR analysis of SE-type DAS genes at FDR < 0.10 confirmed male-specific enrichment in dendritic spine morphogenesis (GO:0061001; *Camk2b*, *Reln*, *Lrp8*, *Dbn1*) and transcriptomic overlap with schizophrenia and Huntington’s disease GEO datasets, while female DAS genes were enriched in synapse organization (GO:0050808; *Ntng1*, *Ptprd*, *Lrfn5*, *Nrcam*) and autism spectrum disorder transcriptomic signatures, consistent with and extending the primary IPA findings. Threshold-free fGSEA of SE-type splicing events further confirmed that synaptic signaling (GOBP SYNAPTIC SIGNALING: male NES =  + 1.97, padj < 0.001; female NES =  + 1.98, padj < 0.001), glutamatergic synapse components (GOCC GLUTAMATERGIC SYNAPSE: NES >  + 2.1 in combined-sexes analyses), and chromatin organization (GOBP CHROMATIN ORGANIZATION: NES >  + 2.0 in combined-sexes analyses) were significantly enriched with concordant direction in combined-sexes analyses regardless of ΔPSI threshold, confirming these as shared molecular consequences of prenatal BPA exposure. Sex-divergent fGSEA signals included female-specific enrichment of parallel fiber-to-Purkinje cell synapse components (GOCC PARALLEL FIBER TO PURKINJE CELL SYNAPSE: NES =  + 1.76, padj < 0.01), consistent with the observed female-specific increase in synaptic puncta density, and male-specific enrichment of schizophrenia-related phenotypic pathways (HP SCHIZOPHRENIA: NES =  + 1.72, padj < 0.01). Unsigned fGSEA analysis of SE-type events confirmed that alternative mRNA splicing regulation (GOBP ALTERNATIVE MRNA SPLICING VIA SPLICEOSOME) was significantly enriched in both sexes regardless of splicing direction (male NES =  + 1.29, female NES =  + 1.32; both padj < 0.001), supporting dysregulated RNA processing as a sex-shared core mechanism.

Sex-stratified IPA analyses further revealed distinct enrichment profiles in male and female DAS gene sets. In males, significant multi-gene enrichments included fear-conditioning behavior (*Gtf2ird1*, *Vdac3*; p-value = 1.63E-03), abnormal morphology of muscle (*Camk2d*, *Gtf2ird1*, *Psap*, *Vdac3*; p-value = 1.17E-04), and neuromuscular disease (*Armcx2*, *Mff*, *Pkm*, *Psap*; p-value = 0.020), suggesting a male-biased pattern that may be relevant to BPA-associated motor and neuromuscular dysfunction. Notably, androgen receptor (AR) emerged as a central hub in the male interactome network (Fig. 8B), connecting multiple BPA-responsive DAS genes, including *Gtf2ird1*, *Psap*, *Hnrnpc*, and *Fbxw4*. This is consistent with the well-established role of AR as a transcriptional regulator in the developing brain [48, 49] and with BPA’s documented capacity to antagonize androgen receptor signaling [86], suggesting that disrupted AR-mediated transcriptional networks may contribute to the sex-specificity of the observed splicing alterations. In females, the most significant multi-gene enrichments were hyperactive behavior (*Gabbr2*, *Gtf2ird1*, *Psap*, *Vamp2*; p-value = 3.26E-05), pervasive developmental disorder (*Gabbr2*, *Pecam1*, *Sorbs1*, *Vamp2*; p-value = 3.22E-04), cerebral palsy (*Gabbr2*, *Vamp2*; p-value = 1.54E-04), anxiety (*Gabbr2*, *Gtf2ird1*, *Vamp2*; p-value = 2.43E-03), early-onset neurological disorder (*Gabbr2*, *Gtf2ird1*; p-value = 0.047), and splicing of mRNA (*Ccar1*, *Hnrnpc*; p-value = 0.026), the latter directly reinforcing the dysregulated RNA processing theme central to this study. These female-biased enrichments are consistent with a distinct molecular response pattern in which BPA-responsive DAS genes converge on synaptic transmission, early neurological onset, and behavioral regulation.

At the pathway level, Synaptogenesis Signaling and GABA Receptor Signaling were enriched in the female cerebellum, while Cleavage and Polyadenylation of Pre-mRNA and Splicing of mRNA pathways were enriched in males, together suggesting that BPA-responsive DAS genes disrupt complementary aspects of RNA processing and synaptic organization in a sex-dependent manner. These robust, multi-gene pathway enrichments converge to support the interpretation that cerebellar DAS genes induced by prenatal BPA exposure are associated with ASD-related neurodevelopmental disorders and their frequently comorbid motor and behavioral deficits, with sex-divergent patterns reflecting the underlying sex-specificity of the splicing response. Threshold-free fGSEA and threshold-relaxed EnrichR analyses of SE-type splicing events confirmed this interpretation without dependence on stringent DAS calling cutoffs: synaptic signaling, chromatin organization, ASD phenotypic pathways, and RNA splicing regulation showed concordant enrichment in combined-sexes analysis across all analytical approaches, while female-specific Purkinje cell synapse enrichment and male-specific schizophrenia pathway enrichment provided additional sex-divergent biological signals consistent with the cellular and molecular phenotypes reported here.

A key finding of this study is the identification of 105 transcripts with abnormal alternative splicing patterns in the cerebellum of prenatally BPA-exposed rat offspring. To validate these splicing changes, we used HRM qRT-PCR on candidate genes, confirming a significant increase in exon-skipping variants in *Ccar1* exclusively in male offspring, while females remained unaffected. This sex-dependent splicing pattern aligns with broader literature suggesting that the developing male brain is particularly vulnerable to environmentally induced transcriptomic disruption. For instance, our previous studies have shown that prenatal BPA exposure disrupts the alternative splicing pattern of genes associated with ASD in the prefrontal cortex and hippocampus, driving male-specific deficits in learning and memory [61, 62]. Our data reveal that male vulnerability extends to the cerebellum, specifically at the level of post-transcriptional alternative splicing. Among the specific targets of BPA-induced splicing dysregulation, *Ccar1* (Cell division cycle and apoptosis regulator 1) emerged as a significantly altered candidate. CCAR1 is a critical chromatin-remodeling protein that regulates gene expression and apoptotic pathways during neurodevelopment [87–89]. Notably, our data indicate that prenatal BPA exposure is associated with increased skipping of the exon encoding the SAP domain of *Ccar1* in the male rat cerebellum. The SAP domain is a crucial nucleic acid-binding motif required for anchoring the protein to chromatin and acting as a transcriptional co-activator [79]. Exon skipping in *Ccar1* may alter a region encoding the SAP domain and could affect CCAR1 function; however, the protein-level consequences were not directly tested here. In both rodent models and human cohorts, mutations or epigenetic dysregulation of *Ccar1* have been shown to cause profound transcriptional deficits, impairing neuronal communication and behavior [89, 90]. Moreover, the widespread dysregulation of chromatin remodeling is a well-established mechanism implicated in ASD and other neurodevelopmental disorders [91, 92]. Sex-dependent disruption of *Ccar1* splicing at the exon encoding the SAP domain may represent a plausible molecular contributor to ASD-relevant neurodevelopmental pathways; however, further functional analyses are required in future studies to fully elucidate how this domain interacts within the developing cerebellum.

In addition to *Ccar1* splicing aberrant, we observed a trending, albeit not strictly significant, alteration in the splicing pattern of *Jmjd1c* (Jumonji Domain Containing 1C) following BPA exposure. *Jmjd1c* is a strong ASD candidate gene (SFARI score 2) [78] encoding a histone demethylase that specifically targets H3K9 and H3K4 methylation [77, 78]. It plays a pivotal role in transcriptional regulation by influencing chromatin structure and the expression of downstream genes involved in synaptic signaling, such as SCN2A and CACNA1H, which are critical for neuronal communication and plasticity [92, 93]. Although the precise functional consequence of the *Jmjd1c* exon-skipping event we observed remains to be fully characterized, the exclusion of this exon may alter JMJD1C protein structure or function, though protein-level consequences were not directly examined here. Such an impairment would restrict the enzyme’s ability to maintain the open chromatin state required for normal neurodevelopment. Clinically, mutations in human JMJD1C have been directly associated with neurodevelopmental deficits, including social impairments and repetitive behaviors—the hallmark symptoms of ASD [93–95]. Notably, the absence of significant changes in the total expression levels of *Ccar1* and *Jmjd1c*, despite their significant DAS, is particularly noteworthy. This indicates that differential expression (DE) analyses alone would have missed the subtle but critical molecular disruptions caused by BPA. Our findings thus underscore the importance of evaluating the alternative splicing landscape to fully capture the scope of environmental endocrine disruption in the developing cerebellum. These results suggest that BPA exposure is associated with aberrant splicing of *Ccar1* and, to a lesser extent, *Jmjd1c*, and that upstream regulators of these target exons warrant further investigation.

We next explored potential upstream regulators that might contribute to the observed exon-skipping events, specifically those observed in *Ccar1* and *Jmjd1c.* Splicing events are regulated by RNA-binding proteins (RBPs), and our in-silico network predictions identified a shared subset of RBPs that regulate both the *Ccar1* and *Jmjd1c* target exons. Notably, using molecular docking, we found that these analyses predicted favorable interactions between BPA and the candidate RBPs CPEB1, RALYL, HNRNPDL, and ACO1. Additionally, docking analyses predicted favorable BPA interactions for several RBPs previously implicated in ASD-related biology, including ELAVL2 [96], ELAVL3, HNRNPD, and PUF60 [97] (Supplementary Fig. 3). Crucially, BPA demonstrated more favorable predicted binding affinities than the single representative ribonucleoside of each RBP’s consensus motif. These findings support a model in which BPA may perturb RBP-associated regulation of *Ccar1* and *Jmjd1c* pre-mRNA splicing, thereby contributing to the observed exon-skipping patterns. These analyses indicate a plausible mechanism for the interactions between BPA and key RBP regulators in aberrant splicing events in the cerebellum. Furthermore, within-gene permutation testing demonstrated that predicted binding site densities for CPEB1 and RALYL at *Ccar1* exon 16 were significantly enriched relative to null exon windows across all sex-stratified analyses (p < 0.001), providing statistical support for the selectivity of these regulatory predictions beyond simple motif presence, direct experimental validation via CLIP-seq in the developing rat cerebellum would be required to confirm binding specificity and establish functional relevance in future studies.

These molecular splicing changes were accompanied by cellular phenotypes in primary cerebellar neurons, as demonstrated by our primary cerebellar neuron assays. Specifically, MTS assays showed an increased viability-related signal in male neurons following BPA treatment. SAP domain of CCAR1 canonically functions as a pro-apoptotic transcriptional co-activator for proteins such as p53 [88]; the aberrant SAP isoform could potentially alter CCAR1-associated transcriptional functions relevant to cell survival, although this was not directly tested. One possible interpretation is that altered CCAR1 function may contribute to the increased viability-related signal observed in male neurons; however, apoptosis was not directly measured in this study. Beyond apoptotic activity, CCAR1 regulates the expression of genes involved in neuronal function and synaptic signaling [87, 98]. While the synaptogenesis assays were conducted in vitro, the increase in viability-related signal without a concurrent increase in synaptic puncta density (Syn1/Psd95 density) could reflect a disruption of the intrinsic apoptotic mechanisms normally required for network refinement, which is implicated in male-biased neurodevelopmental disorders. Conversely, female neurons exhibited a fundamentally distinct response trajectory characterized by aberrant synaptogenesis and decreased viability. BPA can act as a potent xenoestrogen [86]. One possible interpretation is that female neurons respond differently to BPA exposure through sex-dependent signaling pathways; however, the specific mechanisms linking increased synaptic puncta to reduced viability remain to be determined. These neuronal assays suggest sex-dependent cellular responses to BPA exposure, with males showing increased viability-related signal and females showing increased synaptic puncta colocalization, together with reduced viability. A sex-dependent pattern was observed in the synaptogenesis assay, with 72 neurites quantified per condition from 3 independent litters; however, statistical inference was performed at the neurite level and should be interpreted accordingly, in which BPA-exposed male neurons showed significant reductions in both Psd95 and Syn1 puncta densities, similar to the synaptogenesis assay in Thongkorn et al. [47], whereas BPA-exposed female neurons showed a significant increase in colocalized Synapse puncta density. The directional consistency of these effects across all three litters, together with small-to-medium effect sizes calculated at the neurite level, supports the biological relevance of the observed differences, while still warranting cautious interpretation given the limited number of litters. (Cohen’s d = 0.34 for Psd95 and 0.48 for Syn1 in males; d = 0.41 for Synapse in females), supports the biological relevance of the observed differences despite the limited number of biological replicates. We acknowledge that statistical inference was performed at the neurite level (n = 72 per condition) and that the study is limited by n = 3 litters per condition and future studies with a greater number of independent litters will be required to fully establish effect magnitude at the litter level.

While a direct statistical link between the splicing data and the cellular phenotype data was not pursued in the present study, given that the RNA-seq animals received continuous prenatal BPA exposure, whereas the primary neuron cultures were derived from postnatal day 1 pups and maintained in vitro under different conditions, the convergence of male-biased splicing changes and male-biased cellular phenotypes is consistent with a possible link, although these assays were performed under different exposure paradigms and do not establish a direct mechanistic connection. is nonetheless consistent with a model in which BPA-induced alterations in *Ccar1* gene splicing contribute to sex-divergent neurodevelopmental trajectories. Future studies employing matched treatment paradigms, such as isoform-specific knockdown or overexpression in neurons under equivalent BPA exposure conditions, will be required to establish a direct mechanistic link between the observed splicing events and cellular outcomes.

To our knowledge, this is the first study to show that prenatal BPA exposure is associated with disruption of the alternative splicing landscape in the neonatal rat cerebellum, along with predicted interactions between BPA and candidate RNA-binding proteins (RBPs). Through RNA-seq and pathway enrichment analyses, we identified a broad network of differentially alternative spliced (DAS) genes implicated in critical neurodevelopmental functions and canonical pathways associated with ASD. Notably, these alternative splicing changes differed between males and females and were accompanied by distinct molecular and cellular phenotypes.

Together, these findings support a model in which prenatal BPA exposure may influence neurodevelopment through mechanisms involving RBP-associated RNA processing in the neonatal rat cerebellum. In this dataset, males showed stronger associations between BPA exposure, *Ccar1* splicing changes, and viability-related readouts, while females exhibited neuronal functions consistent with dysregulated synaptic connectivity and decreased viability. While most previous toxicological studies have focused on the hippocampus or prefrontal cortex, our study highlights the developing cerebellum as another highly vulnerable target of BPA. Ultimately, this study highlights alternative splicing as a candidate mechanism relevant to prenatal BPA-associated cerebellar alterations, even at a dose corresponding to a historical NOAEL used in earlier regulatory contexts. 

## Conclusion

This study provides new evidence that prenatal BPA exposure is associated with sex-dependent molecular and cellular alterations in cerebellar development in rat offspring. Taken together, motif analysis, docking predictions, and transcript-level data support a hypothetical model in which BPA may perturb RBP-associated splicing regulation of genes such as *Ccar1* (e.g., through candidate RBPs, including CPEB1, RALYL, ACO1, and HNRNPDL) in the male cerebellum, while the upstream mechanisms driving the female-specific phenotype remain to be determined. These transcriptomic alterations were accompanied by distinct cellular outcomes, including increased viability-related signals in males and, in females, increased synaptic puncta colocalization alongside reduced viability. Together, these findings highlight the developing cerebellum as a sensitive and relatively underexplored target of prenatal BPA exposure. This study supports the possibility that environmental factors may contribute to neurodevelopmental disorders through post-transcriptional dysregulation, underscoring the need for future behavioral studies to link these sex-dependent cerebellar alterations to in vivo neurodevelopmental outcomes.

## Supplementary Information

Additional file 1.

Additional file 2.

Additional file 3.

Additional file 4.

Additional file 5.

Additional file 6.

Additional file 7.

Additional file 8.

Additional file 9.

Additional file 10.

Additional file 11.

Additional file 12.

Additional file 13.

## Data Availability

The RNA-seq data generated in this study have been submitted to the NCBI Gene Expression Omnibus (GEO). The accession number will be provided upon acceptance or once assigned. During peer review, private reviewer access can be made available upon request.
