# Supplementary material for: Prenatal BPA exposure perturbs RNA-binding protein-mediated splicing regulation and synaptogenesis in the developing cerebellum in a sex-dependent manner
Source: Biol Sex Differ. 2026 Jun 22;17:149. doi: 10.1186/s13293-026-00941-6 (PMC13540958; doi:10.1186/s13293-026-00941-6)
Supplement: Supplementary file 1 [file 13293_2026_941_MOESM11_ESM.pdf]

A

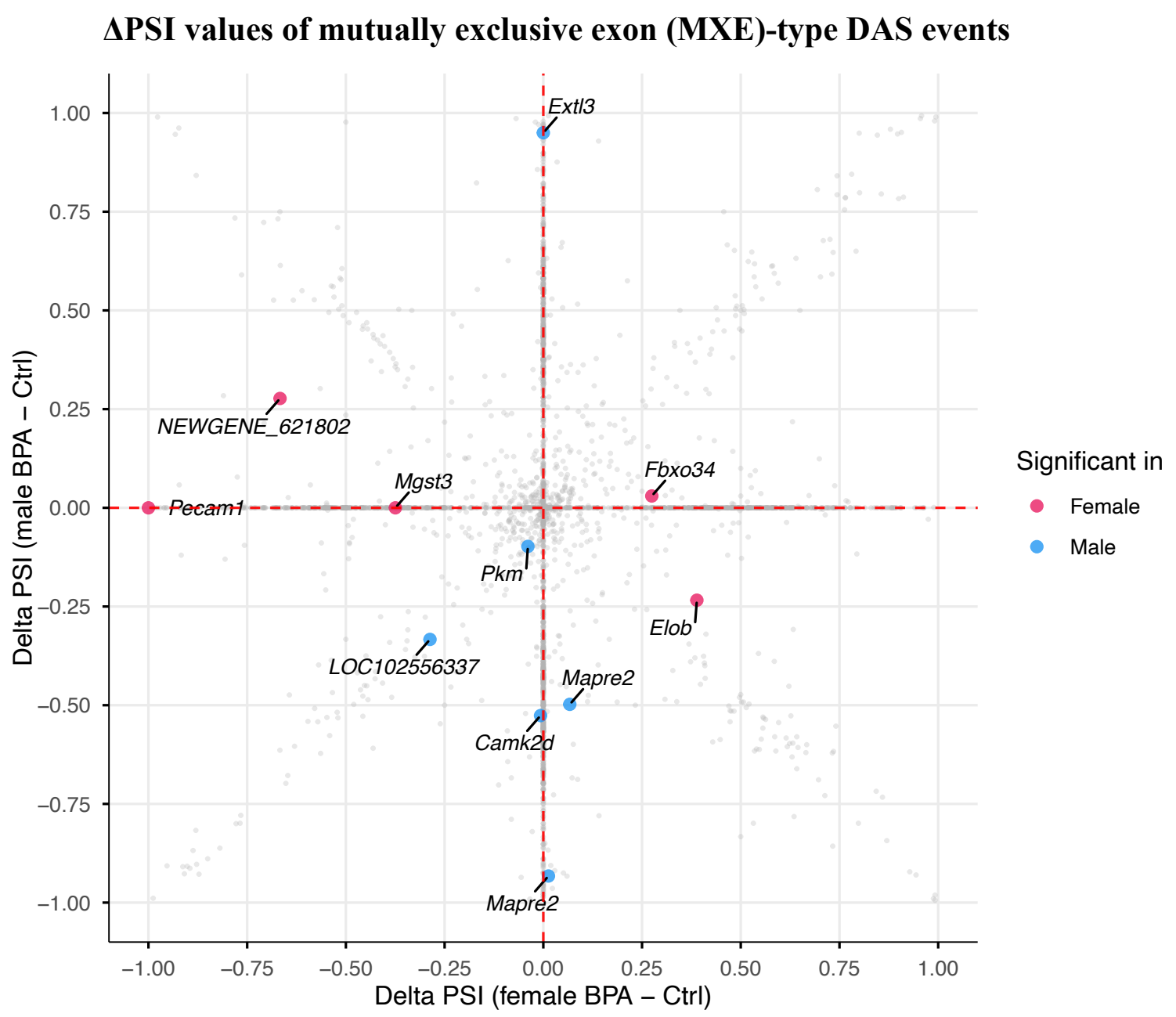
ΔPSI values of mutually exclusive exon (MXE)-type DAS events
Delta PSI (male BPA – Ctrl)
Delta PSI (female BPA – Ctrl)
Extl3
NEWGENE_621802
Pecam1
Mgst3
Fbxo34
Pkm
Elob
LOC102556337
Mapre2
Camk2d
Mapre2
Significant in
Female
Male

B

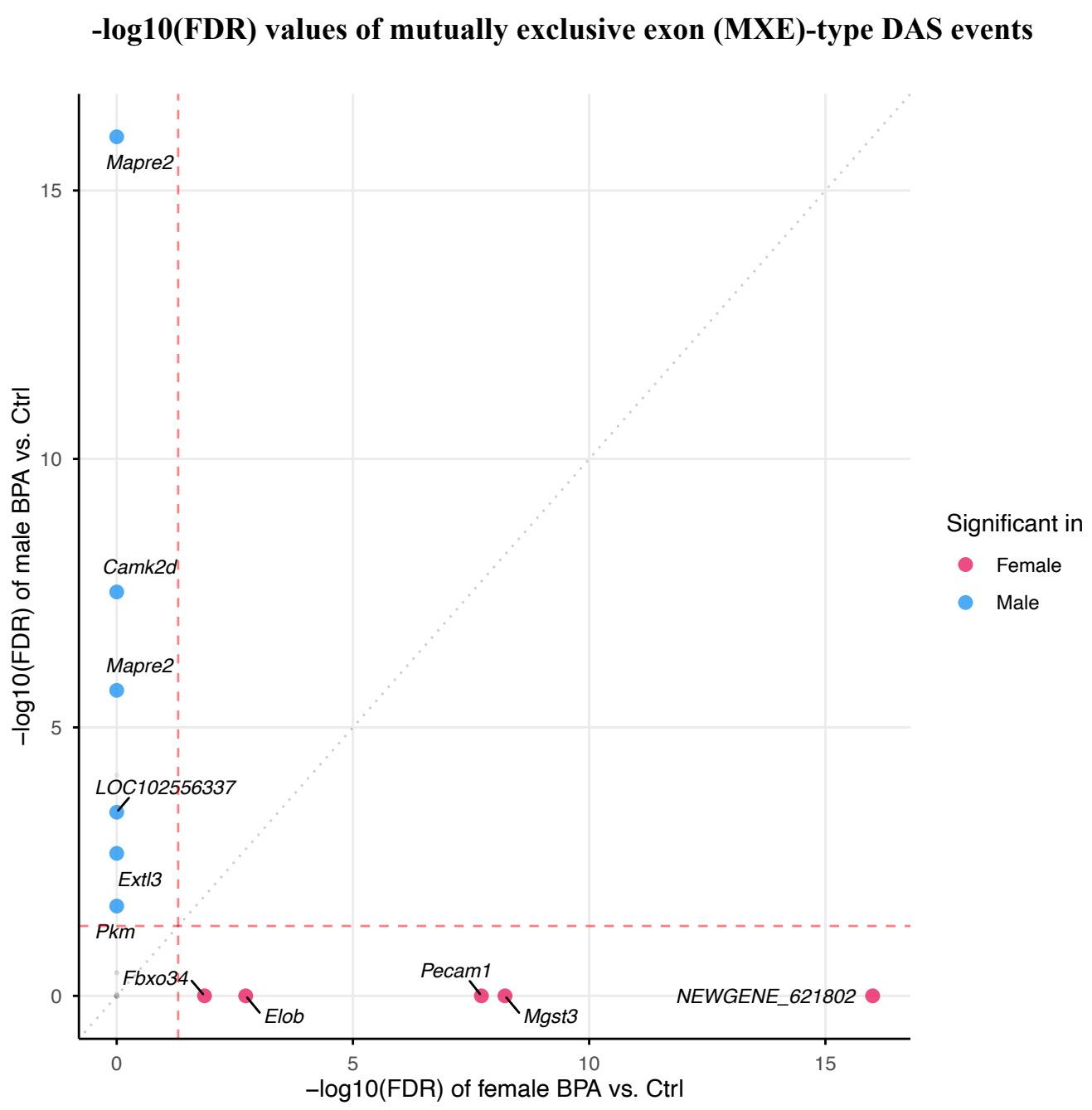
-log10(FDR) values of mutually exclusive exon (MXE)-type DAS events
−log10(FDR) of male BPA vs. Ctrl
−log10(FDR) of female BPA vs. Ctrl
Mapre2
Camk2d
Mapre2
LOC102556337
Extl3
Pkm
Fbxo34
Elob
Pecam1
Mgst3
NEWGENE_621802
Significant in
Female
Male

C

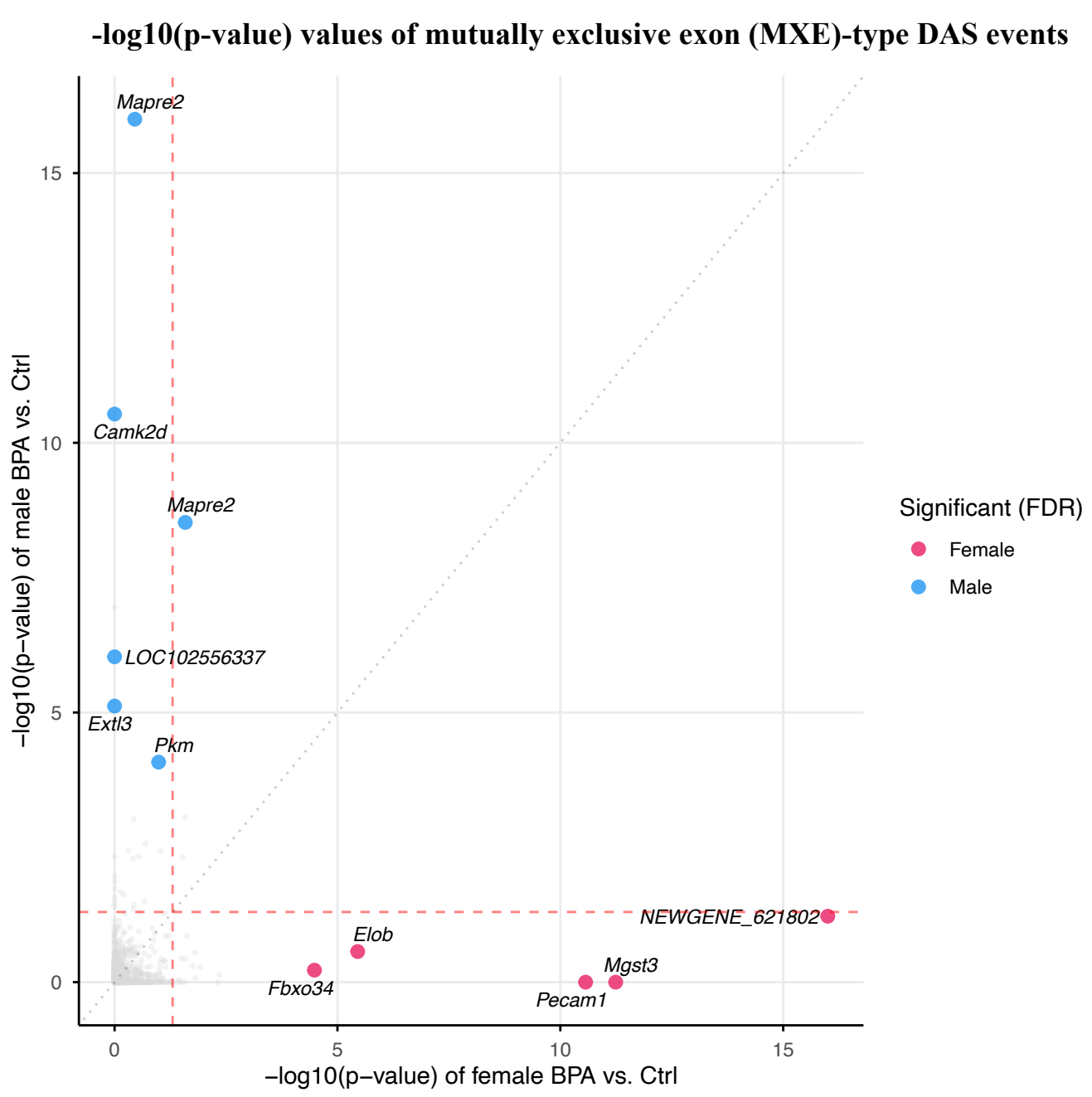
-log10(p-value) values of mutually exclusive exon (MXE)-type DAS events
−log10(p−value) of male BPA vs. Ctrl
−log10(p−value) of female BPA vs. Ctrl
Mapre2
Camk2d
Mapre2
LOC102556337
Extl3
Pkm
NEWGENE_621802
Elob
Mgst3
Fbxo34
Pecam1
Significant (FDR)
Female
Male

D

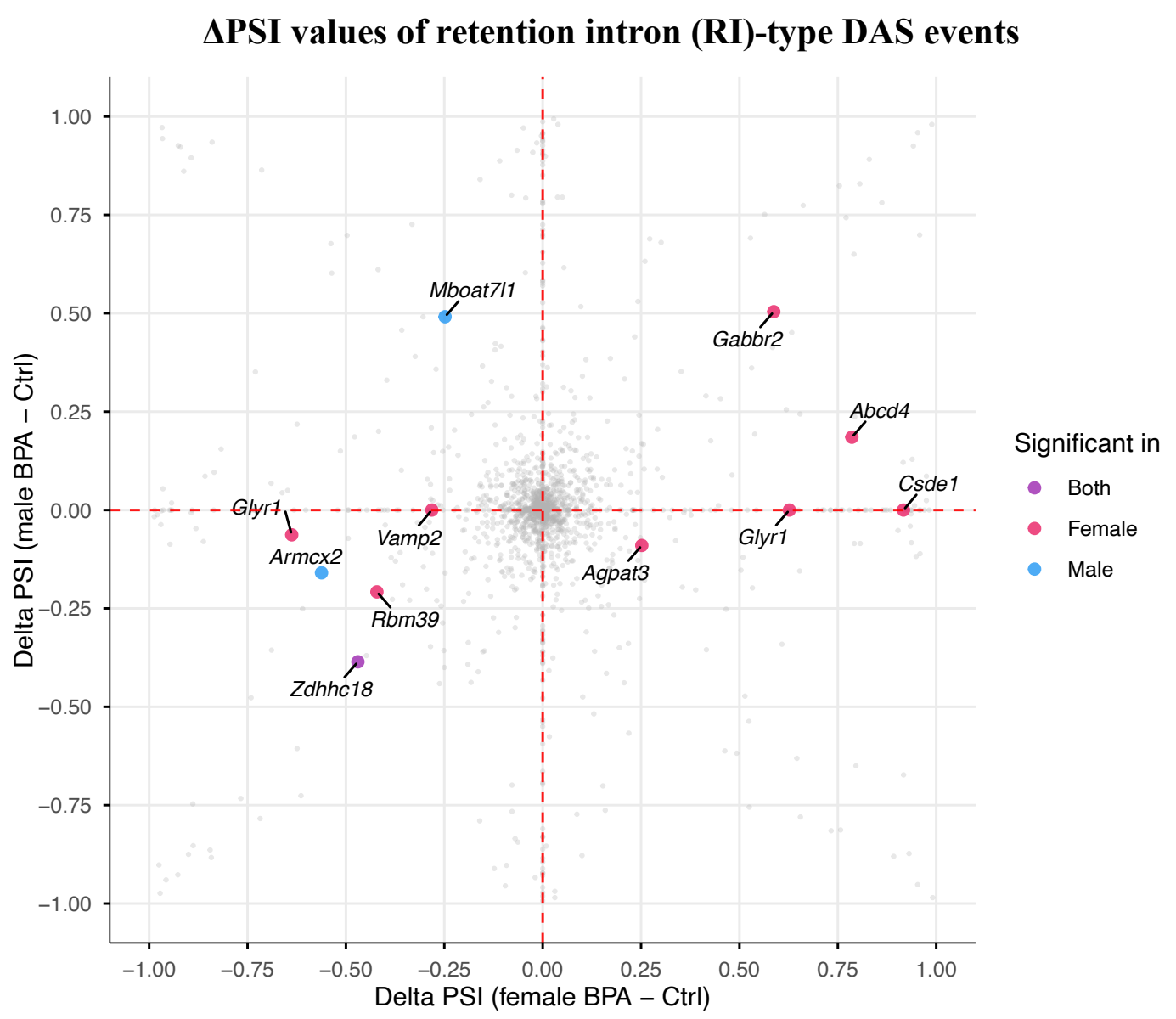
ΔPSI values of retention intron (RI)-type DAS events
Delta PSI (male BPA − Ctrl)
Delta PSI (female BPA − Ctrl)
Mboat7l1
Gabbr2
Abcd4
Csde1
Glyr1
Armcx2
Vamp2
Glyr1
Agpat3
Rbm39
Zdhhc18
Significant in
Both
Female
Male

E

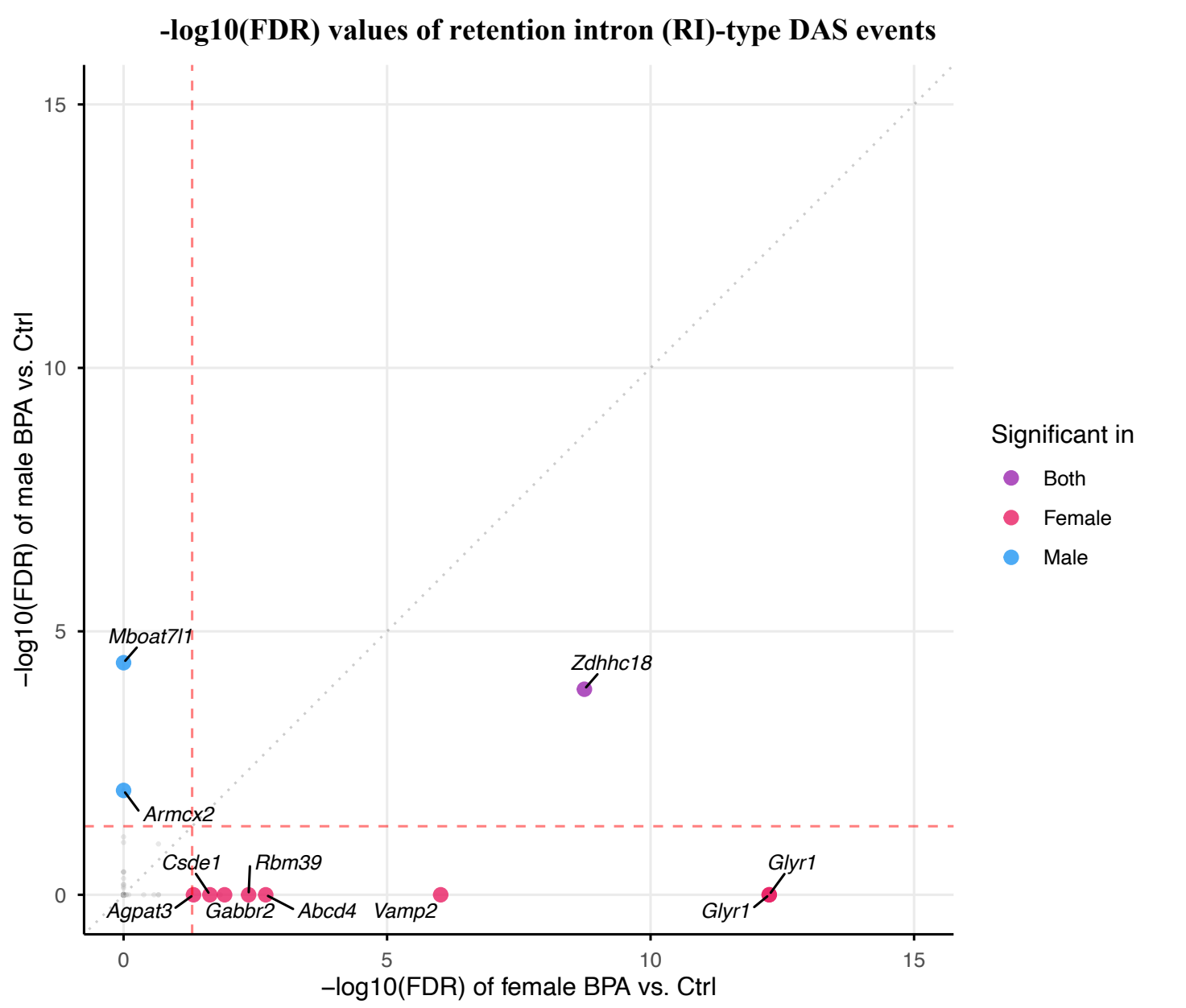
-log10(FDR) values of retention intron (RI)-type DAS events
−log10(FDR) of male BPA vs. Ctrl
−log10(FDR) of female BPA vs. Ctrl
Mboat7l1
Zdhhc18
Armcx2
Csde1
Rbm39
Glyr1
Agpat3
Gabbr2
Abcd4
Vamp2
Glyr1
Significant in
Both
Female
Male

F

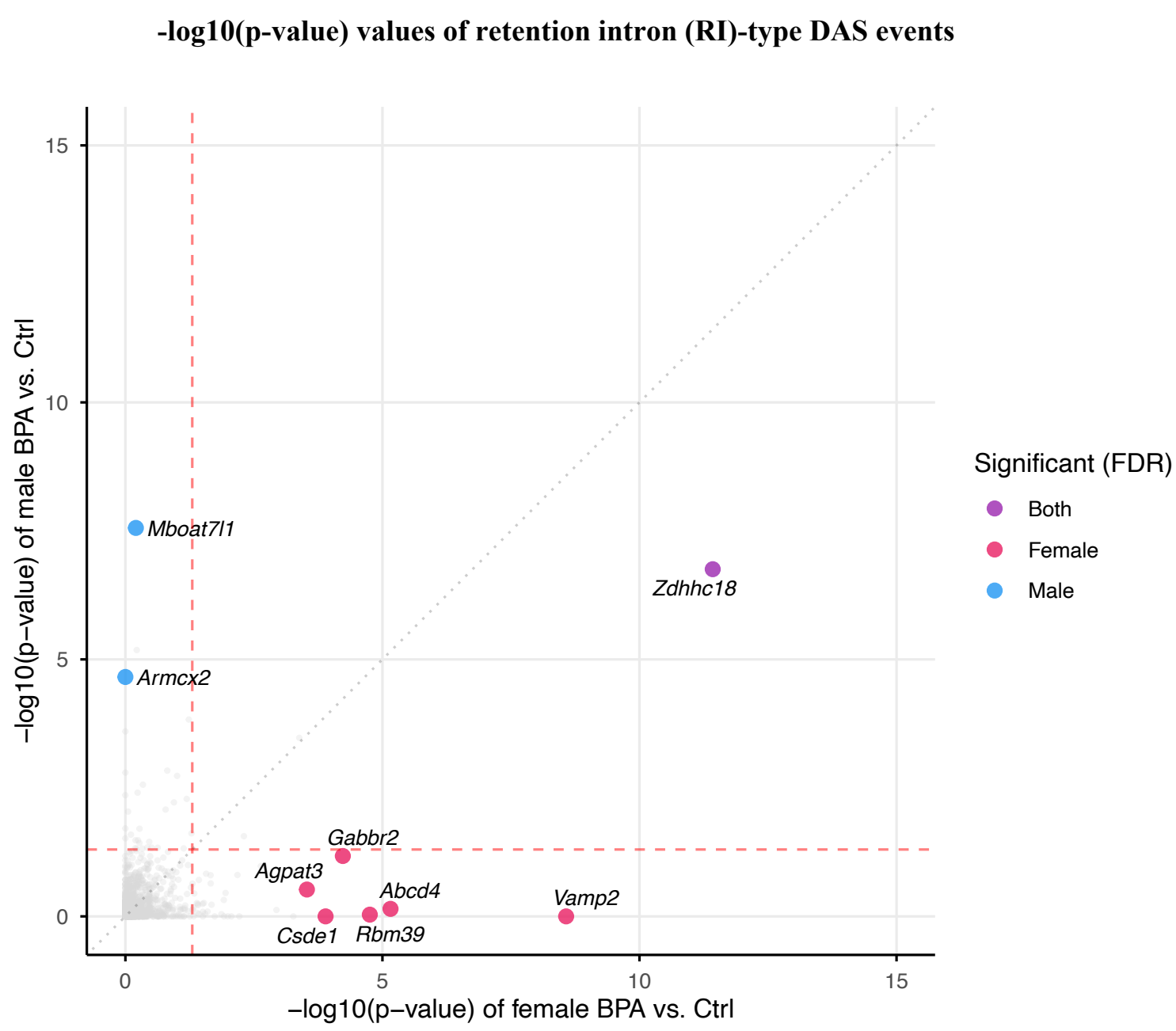
-log10(p-value) values of retention intron (RI)-type DAS events
−log10(p−value) of male BPA vs. Ctrl
−log10(p−value) of female BPA vs. Ctrl
Mboat7l1
Zdhhc18
Armcx2
Gabbr2
Agpat3
Abcd4
Vamp2
Csde1
Rbm39
Significant (FDR)
Both
Female
Male

Supplementary Figure 1. Sex-specific differential alternative splicing patterns of MXE and RI events in the cerebellum of rat pups prenatally exposed to BPA.

(A) Scatter plot of ΔPSI values (BPA − Control) for mutually exclusive exon (MXE)-type DAS events, comparing male (y-axis) versus female (x-axis) offspring. Colored points indicate genes significant in males only (blue) or females only (pink). Gray points represent non-significant events. Labeled genes represent sex-specific MXE-DAS candidates. **(B)** Scatter plot of $-\log_{10}$(FDR) values for MXE-type events, comparing male versus female analyses. Color coding as in panel A. **(C)** Scatter plot of $-\log_{10}$(raw p-value) for MXE-type events, male versus female, as a complementary visualization to panel B. Color coding as in panels A–B. **(D)** Scatter plot of ΔPSI values for intron retention (RI)-type DAS events, comparing male versus female offspring. Colored points indicate genes significant in males only (blue), females only (pink), or both sexes (purple). Labeled genes represent sex-specific RI-DAS candidates. **(E)** Scatter plot of $-\log_{10}$(FDR) values for RI-type events, comparing male versus female analyses. Color coding as in panel D. **(F)** Scatter plot of $-\log_{10}$(raw p-value) for RI-type events, male versus female. Color coding as in panels D–E. In all panels, the divergence of significant events from the axes of symmetry confirms that sex-specific DAS findings reflect biological divergence rather than statistical power artifacts. FDR threshold < 0.05 with $|\Delta PSI| \geq 0.1$.
