# Supplementary material for: Prenatal BPA exposure perturbs RNA-binding protein-mediated splicing regulation and synaptogenesis in the developing cerebellum in a sex-dependent manner
Source: Biol Sex Differ. 2026 Jun 22;17:149. doi: 10.1186/s13293-026-00941-6 (PMC13540958; doi:10.1186/s13293-026-00941-6)
Supplement: Supplementary file 2 [file 13293_2026_941_MOESM12_ESM.pdf]

A

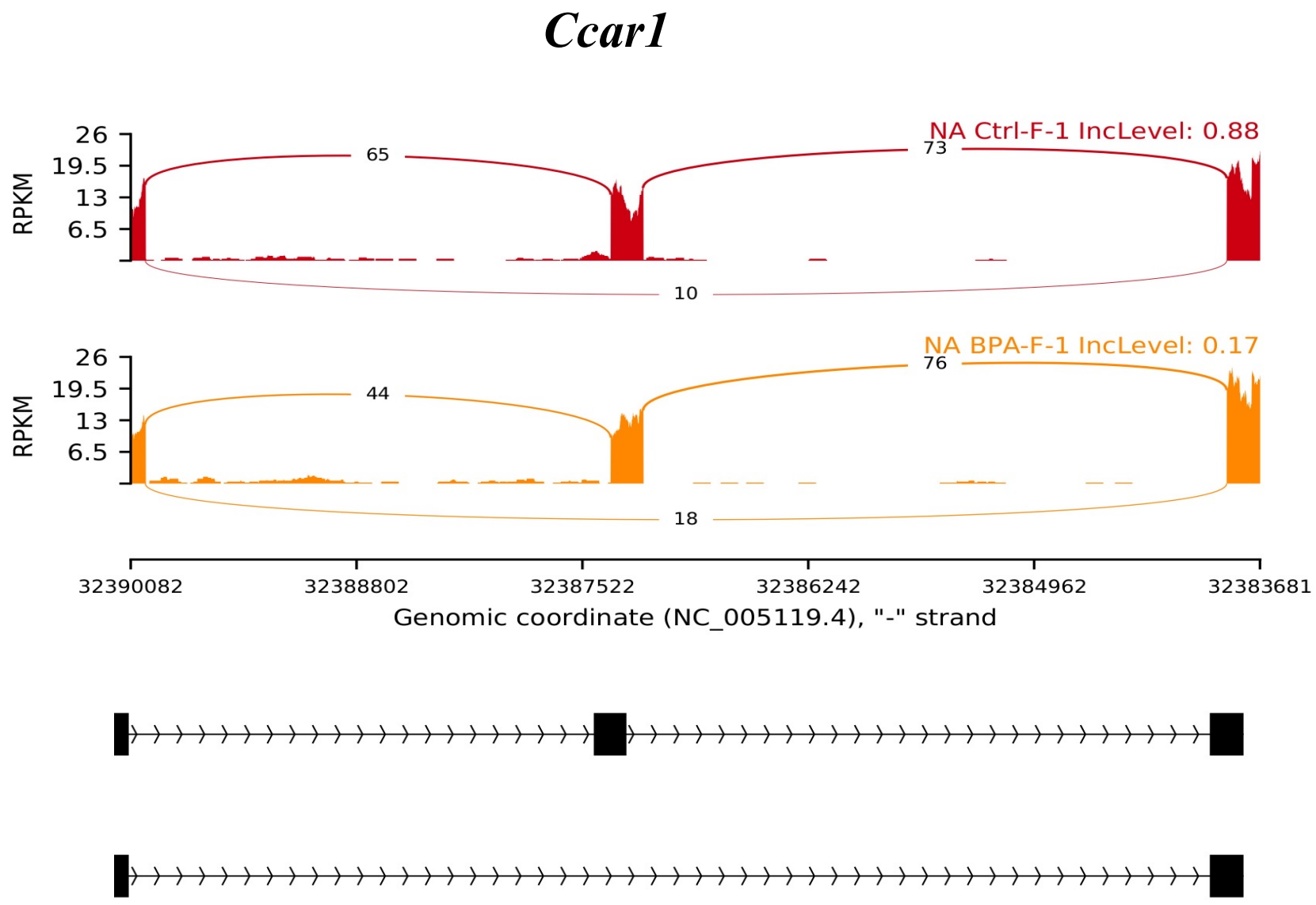
Ccar1
NA Ctrl-F-1 IncLevel: 0.88
RPKM
26
19.5
13
6.5
65
73
10
NA BPA-F-1 IncLevel: 0.17
RPKM
26
19.5
13
6.5
44
76
18
32390082
32388802
32387522
32386242
32384962
32383681
Genomic coordinate (NC_005119.4), "-" strand

B

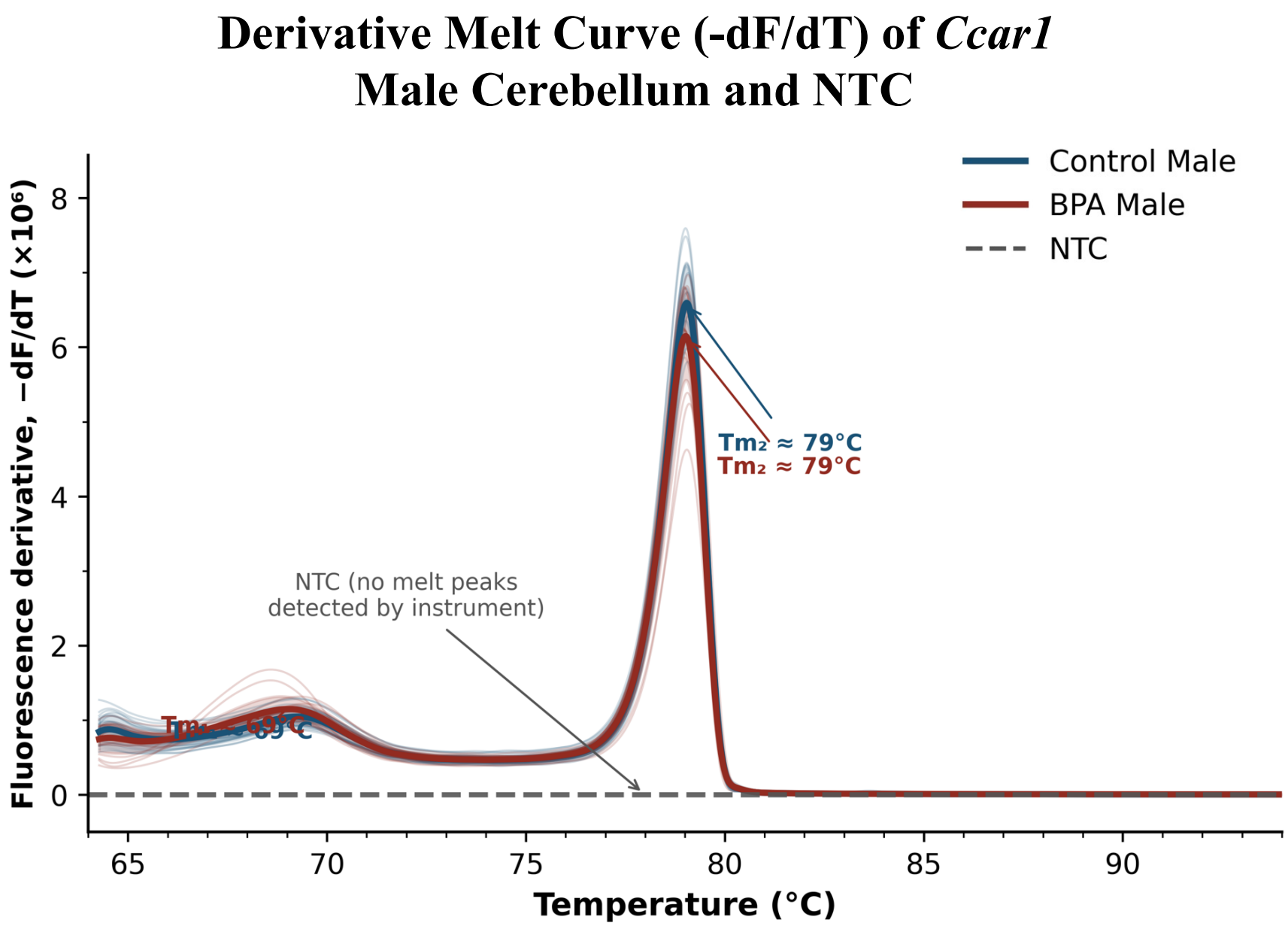
Derivative Melt Curve (-dF/dT) of Ccar1
Male Cerebellum and NTC
Control Male
BPA Male
NTC
Fluorescence derivative, −dF/dT (×10⁶)
Tm₂ ≈ 79°C
Tm₂ ≈ 79°C
NTC (no melt peaks
detected by instrument)
Temperature (°C)

C

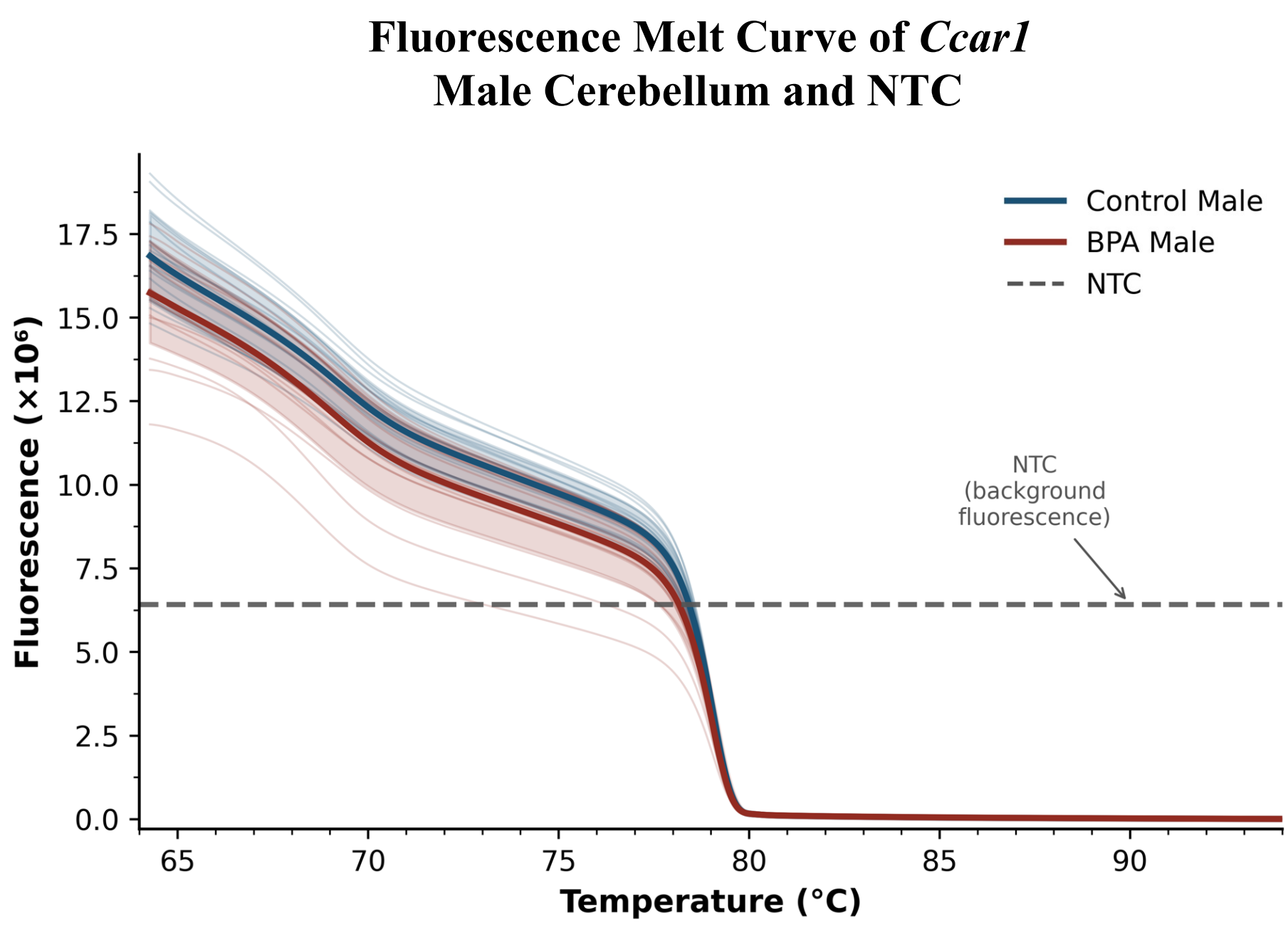
Fluorescence Melt Curve of Ccar1
Male Cerebellum and NTC
Control Male
BPA Male
NTC
Fluorescence (×10⁶)
NTC
(background
fluorescence)
Temperature (°C)

D

Ccar1 inclusion isoform

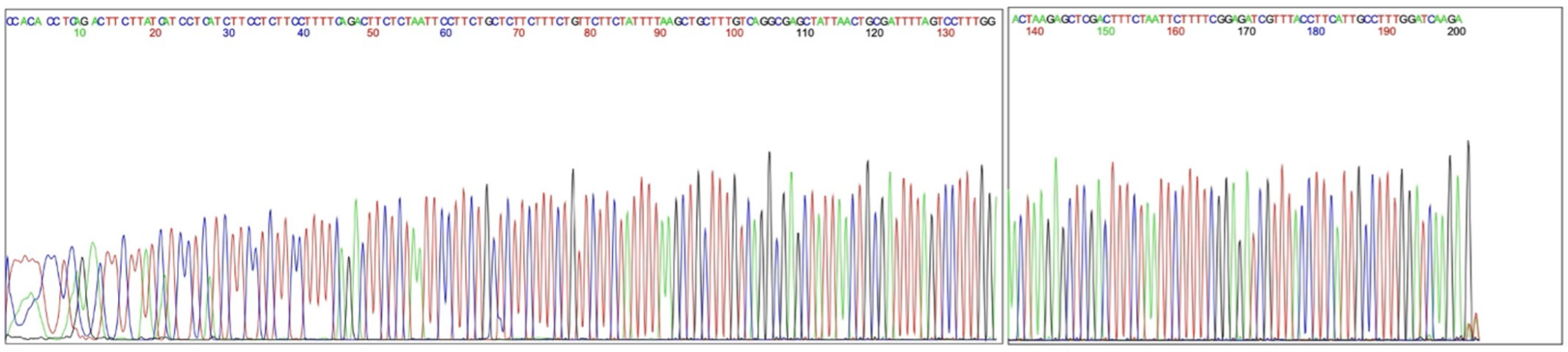

```
Length=201

                                                                  Score     E
Sequences producing significant alignments:                      (Bits)  Value

XM_032889281.1 PREDICTED: Rattus rattus cell division cycle and a...  342     7e-90
XM_008772897.3 PREDICTED: Rattus norvegicus cell division cycle a...  342     7e-90
XM_039098883.1 PREDICTED: Rattus norvegicus cell division cycle a...  342     7e-90
XM_008772894.2 PREDICTED: Rattus norvegicus cell division cycle a...  342     7e-90
XM_006256463.3 PREDICTED: Rattus norvegicus cell division cycle a...  342     7e-90
XM_006256462.3 PREDICTED: Rattus norvegicus cell division cycle a...  342     7e-90
FQ228218.1 Rattus norvegicus TL0AEA10YB06 mRNA sequence               342     7e-90
FQ227619.1 Rattus norvegicus TL0AEA12YJ22 mRNA sequence               342     7e-90
FQ233652.1 Rattus norvegicus TL0AEA61YA13 mRNA sequence               342     7e-90
NM_001108535.1 Rattus norvegicus cell division cycle and apoptosi...  342     7e-90


>XM_032889281.1 PREDICTED: Rattus rattus cell division cycle and apoptosis regulator
1 (Ccar1), mRNA
Length=4333
```

E

Ccar1 exclusion isoform

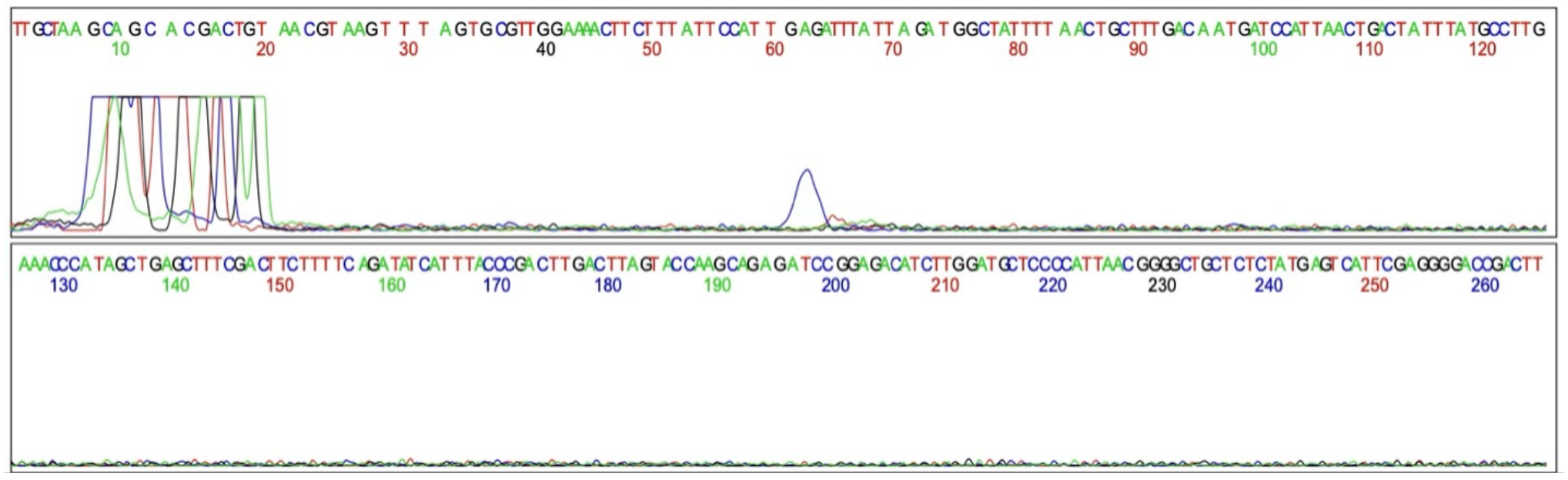

F

Jmjd1c

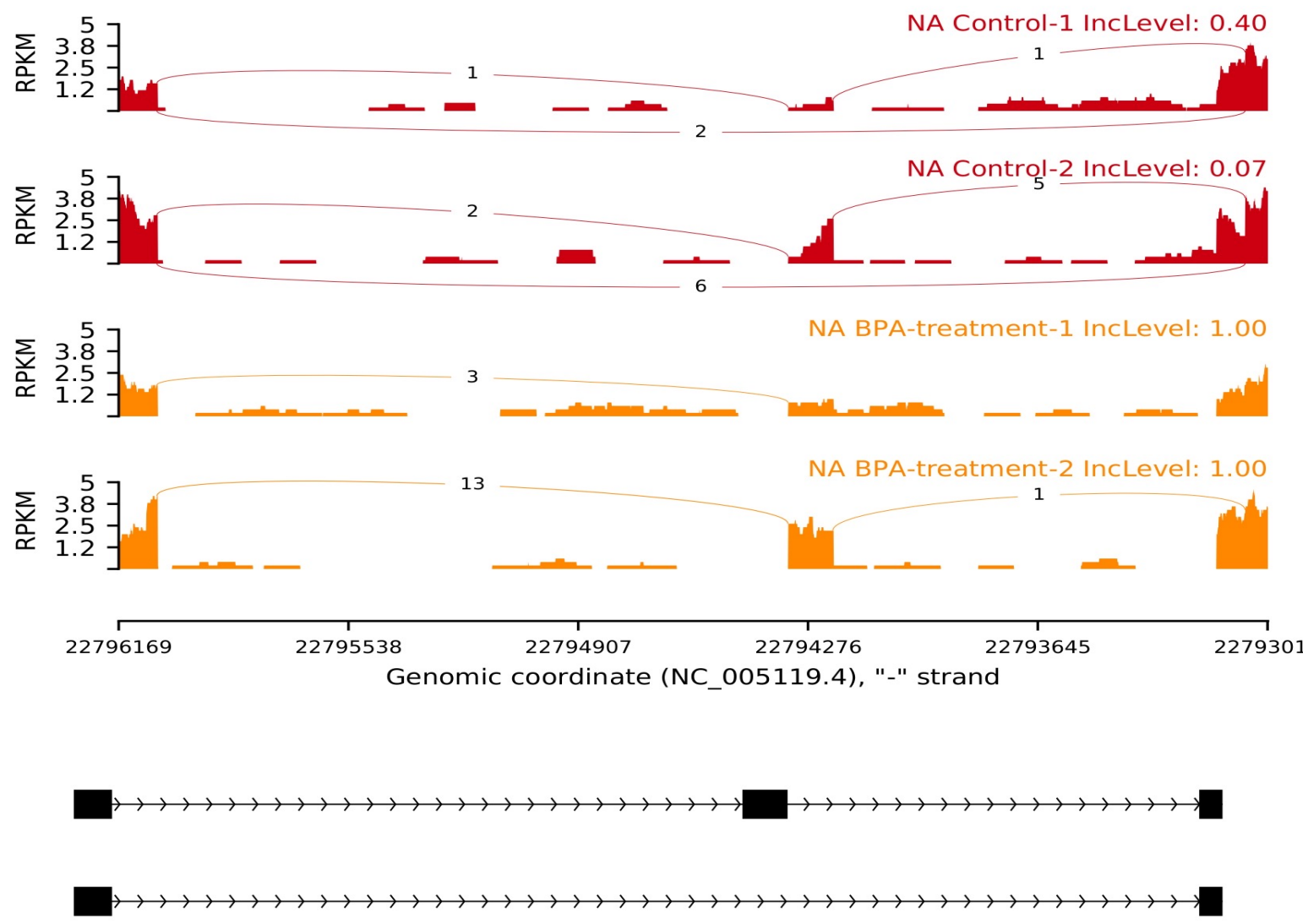

G

Derivative Melt Curve (-dF/dT) of *Jmjd1c* Male Cerebellum and NTC

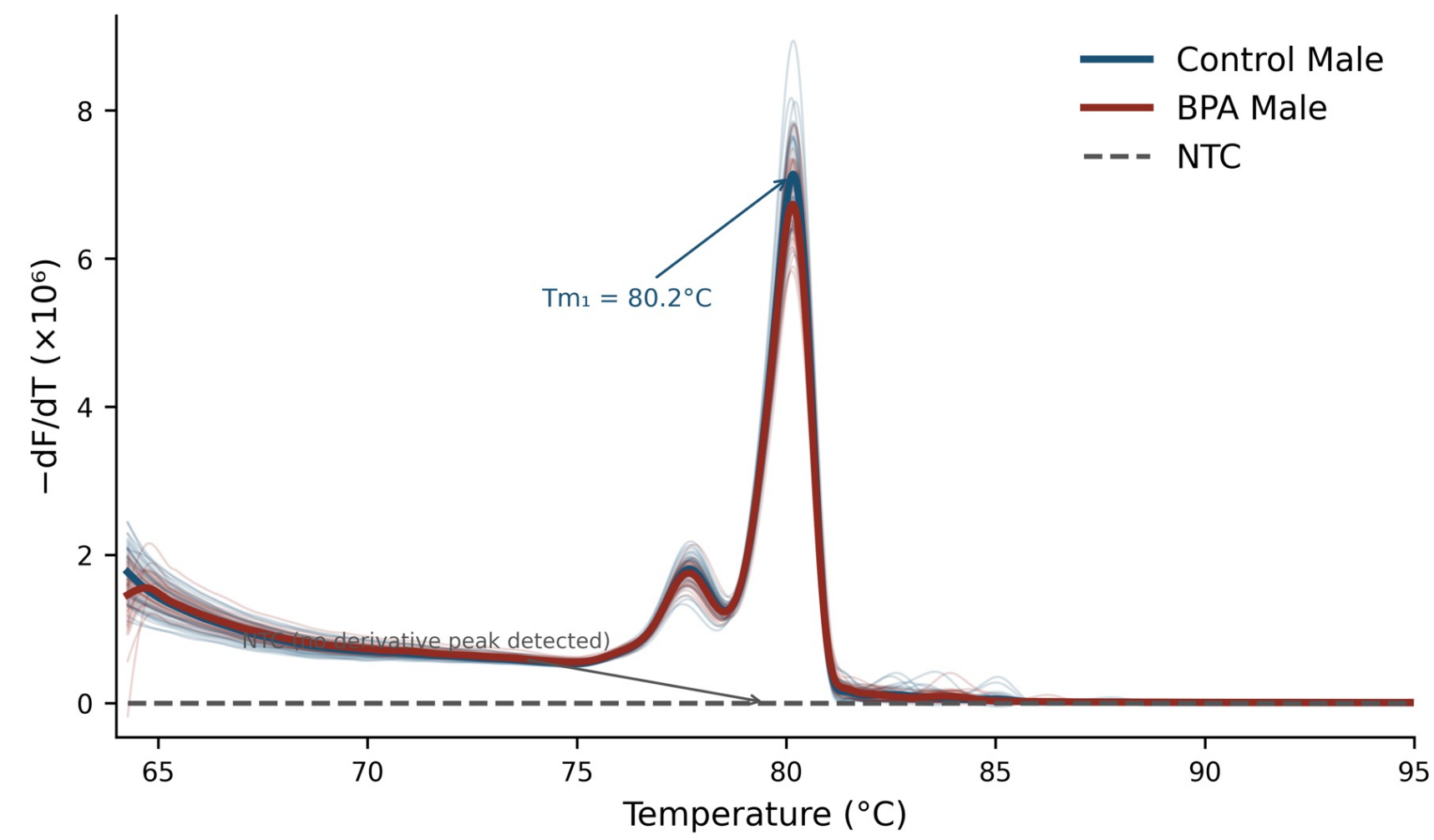

H

Fluorescence Melt Curve of *Jmjd1c* Male Cerebellum and NTC

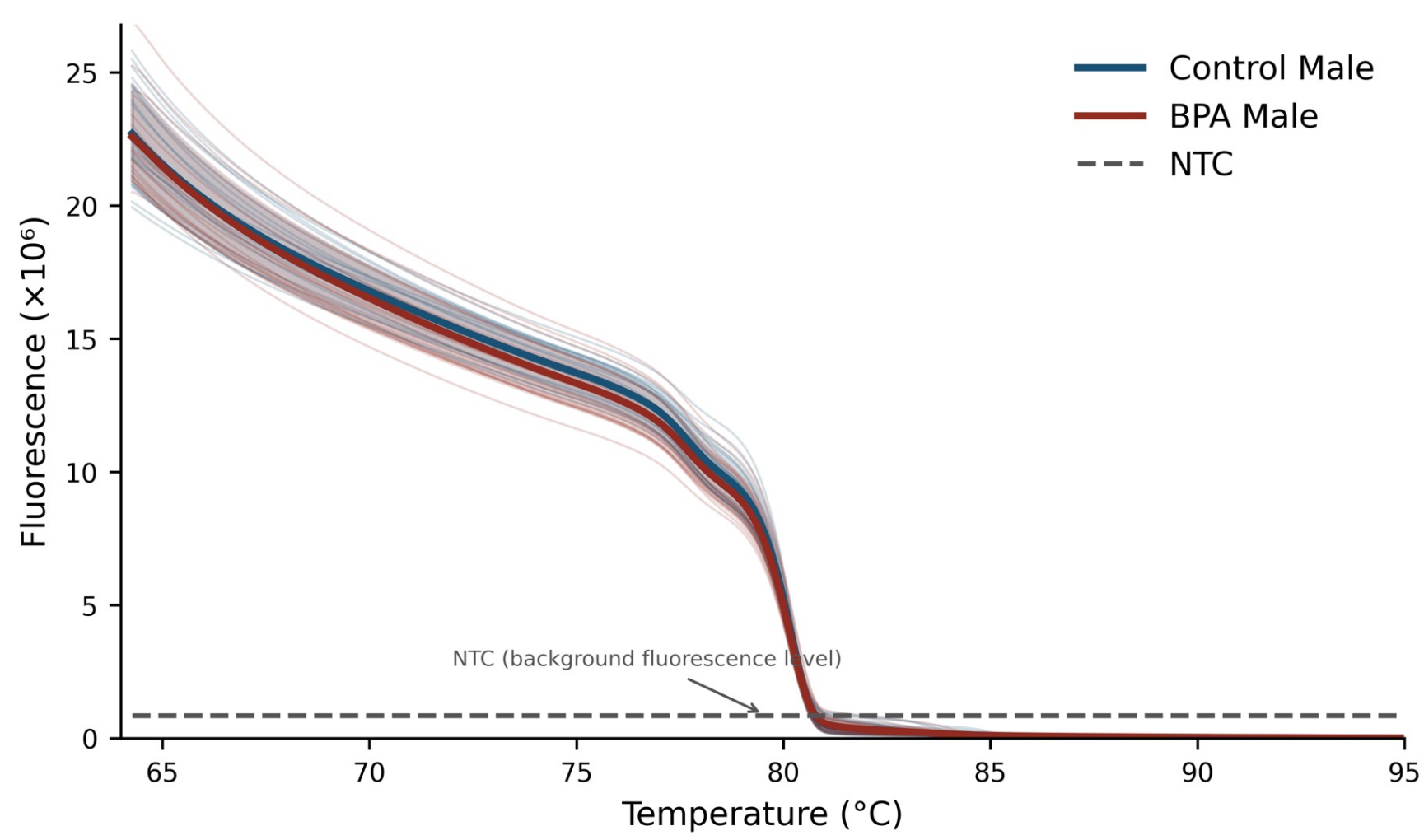

I

Jmjd1c inclusion isoform

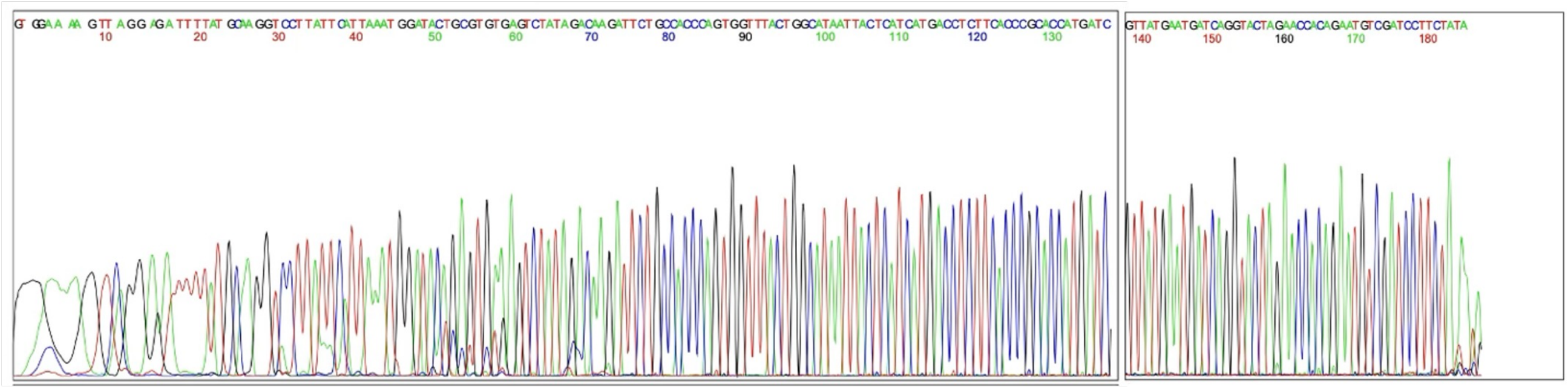

Length=184

Sequences producing significant alignments:	Score (Bits)	E Value
NM_001191719.3 Rattus norvegicus jumonji domain containing 1C (Jm...	300	4e-77
XR_005497167.1 PREDICTED: Rattus norvegicus jumonji domain contai...	300	4e-77
XM_006256364.2 PREDICTED: Rattus norvegicus jumonji domain contai...	300	4e-77
XM_039098406.1 PREDICTED: R... Show report for XR_005497167.1 ... domain contai...	300	4e-77
XM_039098405.1 PREDICTED: R... domain contai...	300	4e-77
XM_039098403.1 PREDICTED: Rattus norvegicus jumonji domain contai...	300	4e-77
XM_006256361.4 PREDICTED: Rattus norvegicus jumonji domain contai...	300	4e-77
XM_006256362.4 PREDICTED: Rattus norvegicus jumonji domain contai...	300	4e-77
XM_039098402.1 PREDICTED: Rattus norvegicus jumonji domain contai...	300	4e-77
XM_006256359.4 PREDICTED: Rattus norvegicus jumonji domain contai...	300	4e-77

>NM_001191719.3 Rattus norvegicus jumonji domain containing 1C (Jmjd1c), mRNA
Length=8363

J

Jmjd1c exclusion isoform

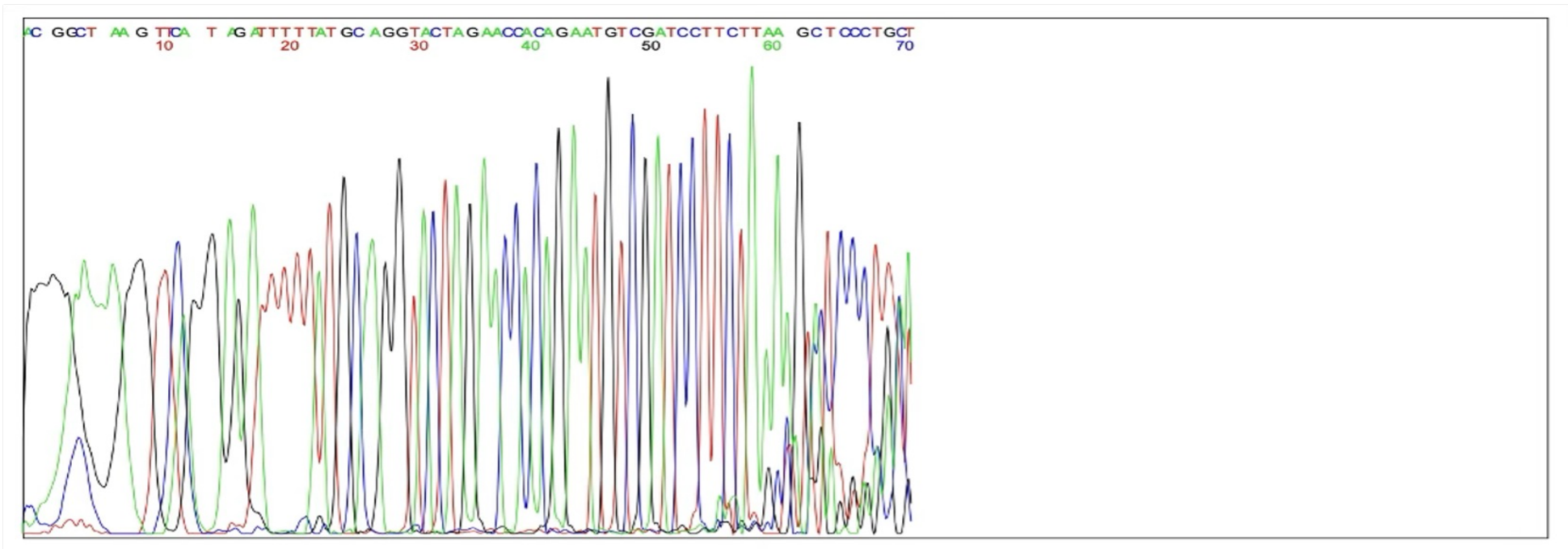

```
Length=68
                                                                    Score     E
Sequences producing significant alignments:                        (Bits)  Value

NM_001191719.3 Rattus norvegicus jumonji domain containing 1C (Jm...  78.7    5e-11
XR_005497167.1 PREDICTED: Rattus norvegicus jumonji domain contai...  78.7    5e-11
XM_006256365.3 PREDICTED: Rattus norvegicus jumonji domain contai...  78.7    5e-11
XM_006256364.2 PREDICTED: Rattus norvegicus jumonji domain contai...  78.7    5e-11
XM_039098406.1 PREDICTED: Rattus norvegicus jumonji domain contai...  78.7    5e-11
XM_039098405.1 PREDICTED: Rattus norvegicus jumonji domain contai...  78.7    5e-11
XM_039098403.1 PREDICTED: Rattus norvegicus jumonji domain contai...  78.7    5e-11
XM_006256361.4 PREDICTED: Rattus norvegicus jumonji domain contai...  78.7    5e-11
XM_006256362.4 PREDICTED: Rattus norvegicus jumonji domain contai...  78.7    5e-11
XM_006256360.4 PREDICTED: Rattus norvegicus jumonji domain contai...  78.7    5e-11

>NM_001191719.3 Rattus norvegicus jumonji domain containing 1C (Jmjd1c), mRNA
Length=8363
```

K

Ccar1 exon 16

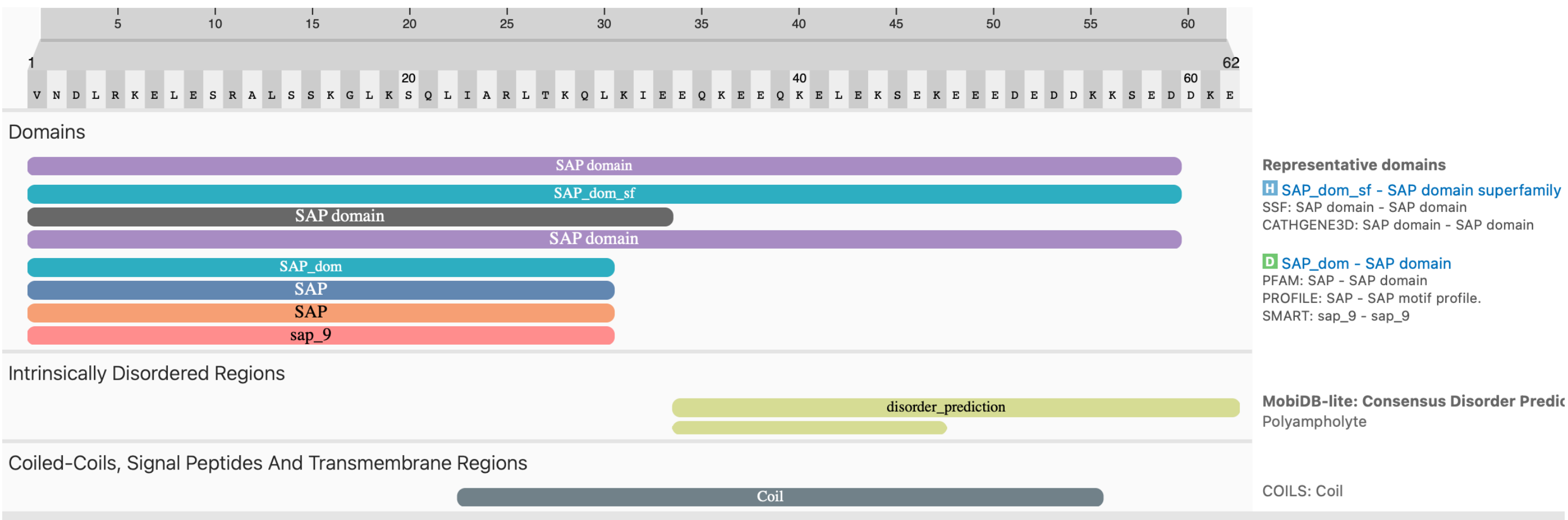

Supplementary Figure 2. HRM qRT-PCR melt curves and Sanger sequencing confirmation of *Ccar1* and *Jmjd1c* splicing isoforms. **(A)** Sashimi plot of *Ccar1* RNA-seq read coverage in representative Control Female (red) and BPA Female (orange) samples, showing inclusion (IncLevel: 0.88 vs 0.17, respectively) and exclusion of exon 16. Exon–intron structure of the inclusion (upper) and exclusion (lower) isoforms is shown below. **(B)** Derivative melt curve (−dF/dT) of Ccar1 HRM qRT-PCR products from male cerebellum samples and no-template control (NTC). Control Male (blue) and BPA Male (dark red) samples share a dominant melt peak at Tm ≈ 79°C corresponding to the inclusion isoform (IC; 230 bp), with a minor shoulder at Tm ≈ 69°C corresponding to the exclusion isoform (EC; 44 bp). No melt peaks were detected in NTC. **(C)** Fluorescence melt curve of *Ccar1* from the same samples as panel B. NTC showed only background fluorescence (dashed line), confirming the absence of non-specific amplification. **(D)** Sanger sequencing chromatogram and NCBI BLAST alignment confirming identity of the *Ccar1* inclusion isoform (IC; 230 bp amplicon; Length = 201 bp sequenced). Top BLAST hits align to Rattus rattus and Rattus norvegicus cell division cycle and apoptosis regulator 1 (*Ccar1*) mRNA (E-value: 7e−90). **(E)** Sanger sequencing chromatogram confirming identity of the *Ccar1* exclusion isoform (EC; 44 bp amplicon). **(F)** Sashimi plot of *Jmjd1c* RNA-seq read coverage in representative Control (red; IncLevel: 0.40 and 0.07) and BPA-treated (orange; IncLevel: 1.00) female samples, showing variable inclusion of exon 5. Exon–intron structure of the inclusion and exclusion isoforms is shown below. **(G)** Derivative melt curve (−dF/dT) of *Jmjd1c* HRM qRT-PCR products from male cerebellum and NTC. A single dominant melt peak at Tm = 80.2°C was observed in both Control Male (blue) and BPA Male (dark red) samples. No derivative peak was detected in NTC. **(H)** Fluorescence melt curve of *Jmjd1c* from the same samples as panel G. NTC showed background fluorescence only (dashed line). **(I)** Sanger sequencing chromatogram and NCBI BLAST alignment confirming identity of the *Jmjd1c* inclusion isoform (IC; 212 bp amplicon; Length = 184 bp sequenced). Top BLAST hits align to Rattus norvegicus jumonji domain containing 1C (Jmjd1c) mRNA (E-value: 4e−77). **(J)** Sanger sequencing chromatogram confirming identity of the *Jmjd1c* exclusion isoform (EC; 81 bp amplicon; Length = 68 bp sequenced). Top BLAST hits align to Rattus norvegicus Jmjd1c mRNA (E-value: 5e−11). **(K)** Protein domain architecture of the *Ccar1* exon 16-encoded peptide region, as predicted by InterPro/MobiDB-lite. The region encodes multiple SAP domain annotations (PFAM, PROFILE, SMART, CATHGene3D), an intrinsically disordered region (MobiDB-lite: polyampholyte), and a coiled-coil region (COILS), consistent with a role in chromatin organization and RNA processing.
