# Supplementary material for: Prenatal BPA exposure perturbs RNA-binding protein-mediated splicing regulation and synaptogenesis in the developing cerebellum in a sex-dependent manner
Source: Biol Sex Differ. 2026 Jun 22;17:149. doi: 10.1186/s13293-026-00941-6 (PMC13540958; doi:10.1186/s13293-026-00941-6)
Supplement: Supplementary file 3 [file 13293_2026_941_MOESM13_ESM.pdf]

# Molecular docking of BPA and common regulatory RBP of *Ccar1* (exon15) and *Jmjd1c* (exon6)

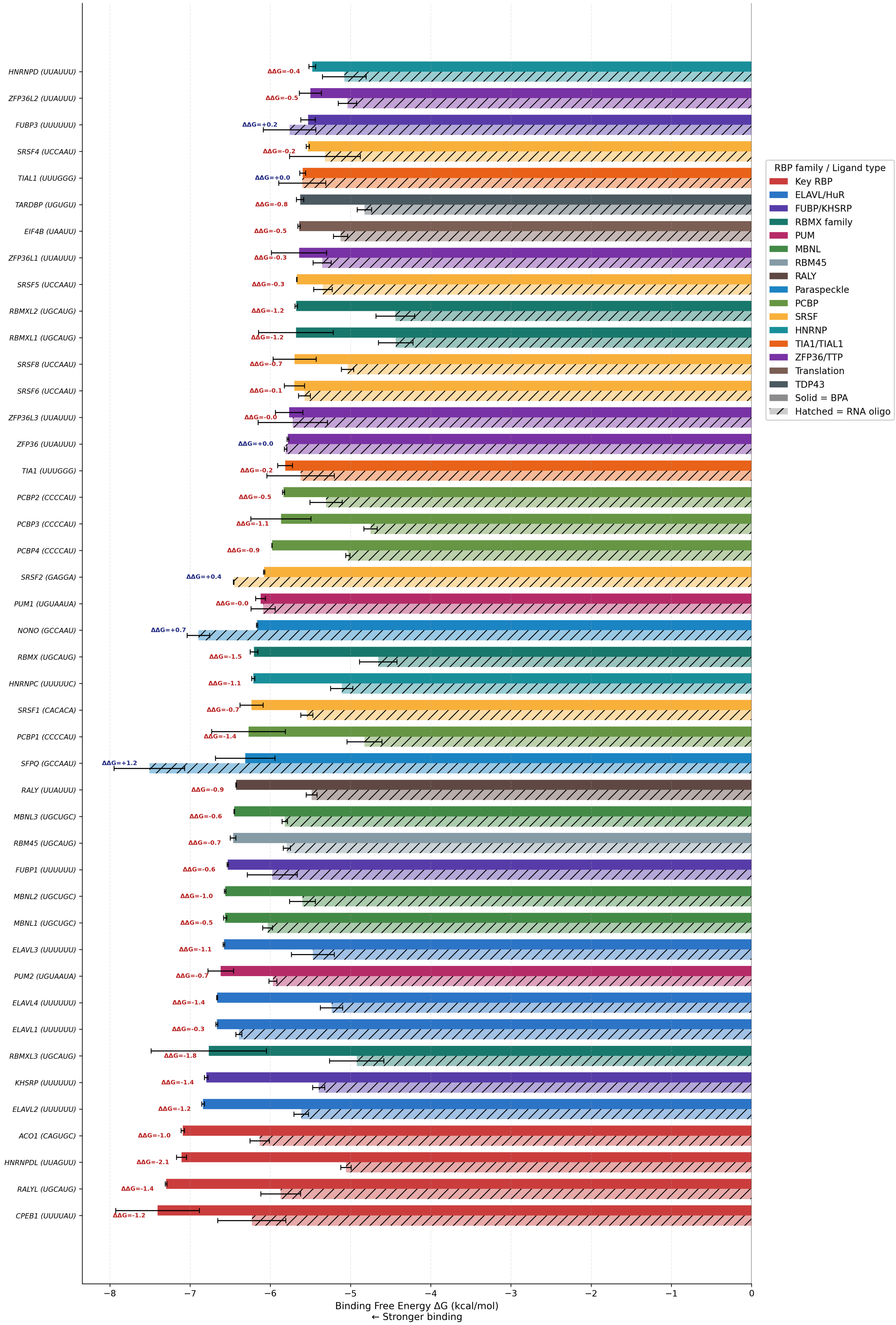

**Supplementary Figure 3. Molecular docking binding affinities of BPA and consensus RNA motif nucleoside against all common regulatory RBPs of *Ccar1* exon 16 and *Jmjd1c* exon 5**

Horizontal bar chart of mean predicted binding free energy (ΔG, kcal/mol ± SD; n = 3 docking replicates) for BPA (solid bars) and the first ribonucleoside of each RBP's experimentally validated consensus RNA motif (hatched bars; sourced from ATtRACT database) docked against all 44 shared RBPs. RBPs are ranked by ΔΔG (BPA ΔG − RNA ΔG), shown in red adjacent to each pair of bars; more negative ΔΔG values indicate greater predicted preference for BPA over the RNA nucleoside proxy. RBP families are color-coded as indicated in the legend. The four candidate RBPs highlighted in the main text (CPEB1, RALYL, HNRNPDL, ACO1) are labeled in red. Error bars represent SD across three independent docking runs. Note: The RNA ligand represents the first ribonucleoside of each RBP's consensus motif as a proxy for the native RNA substrate; full oligonucleotide docking is not supported by AutoDock Vina. ΔΔG values reflect a simplified competitive estimate and should not be interpreted as direct evidence of RNA displacement from RBP binding sites.
